# Supplementary material for: Syzygium aromaticum Phytoconstituents Target SARS-CoV-2: Integrating Molecular Docking, Dynamics, Pharmacokinetics, and miR-21 rs1292037 Genotyping
Source: Viruses. 2025 Jul 5;17(7):951. doi: 10.3390/v17070951 (PMC12300145; doi:10.3390/v17070951)

Supplementary S1

Molecular dynamics between methyl eugenol and target 7NIO protein of SARS-CoV-2.

RMSD

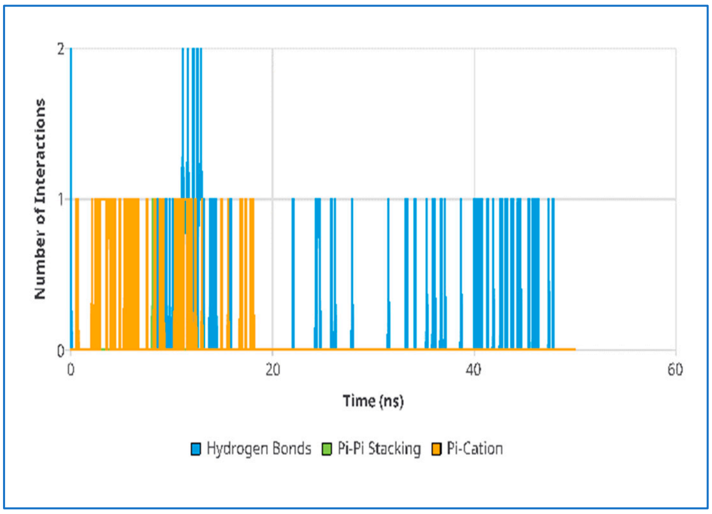

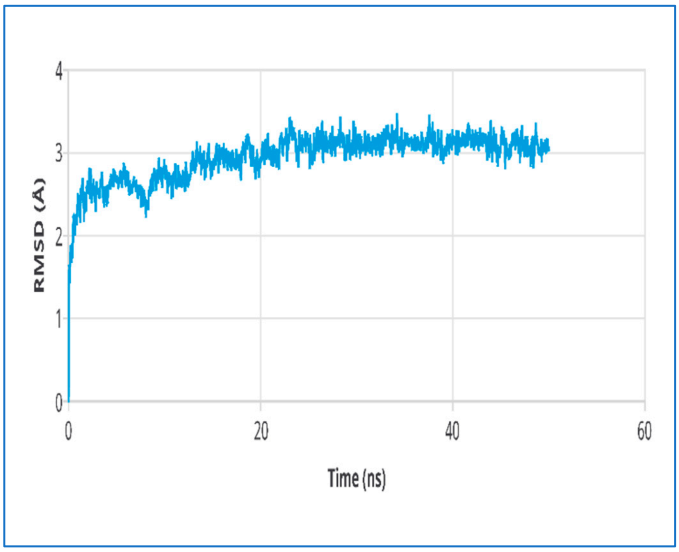

RMSF

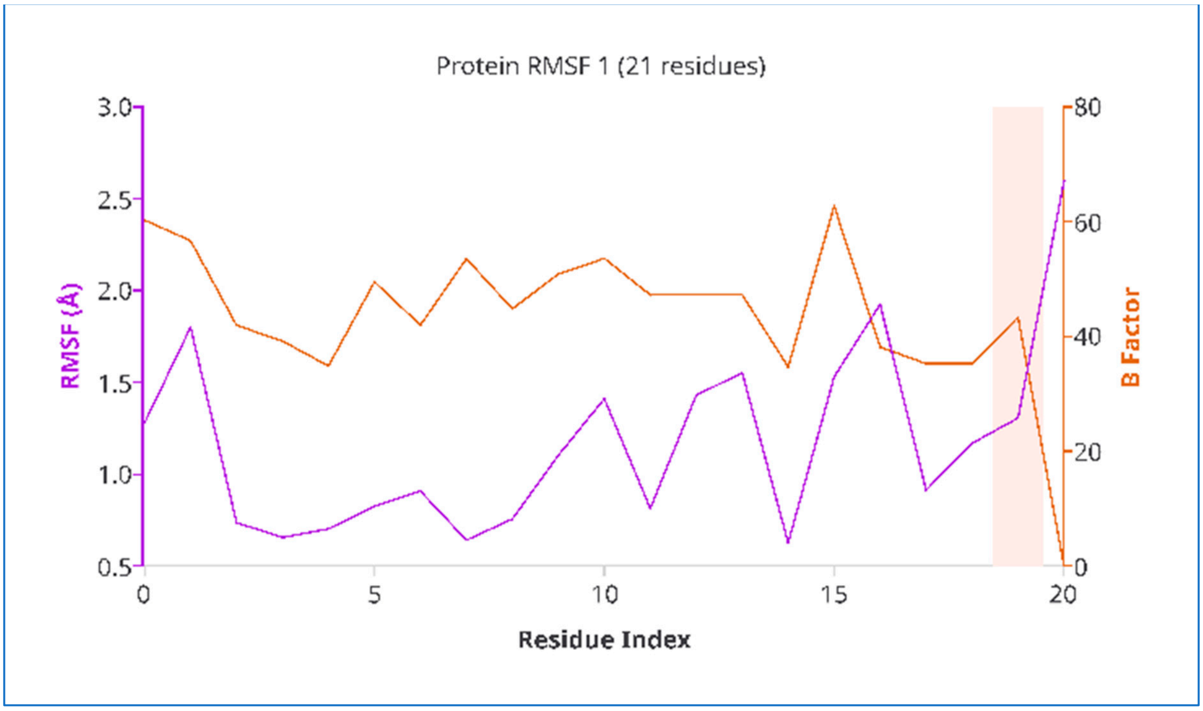

Radius of Gyration

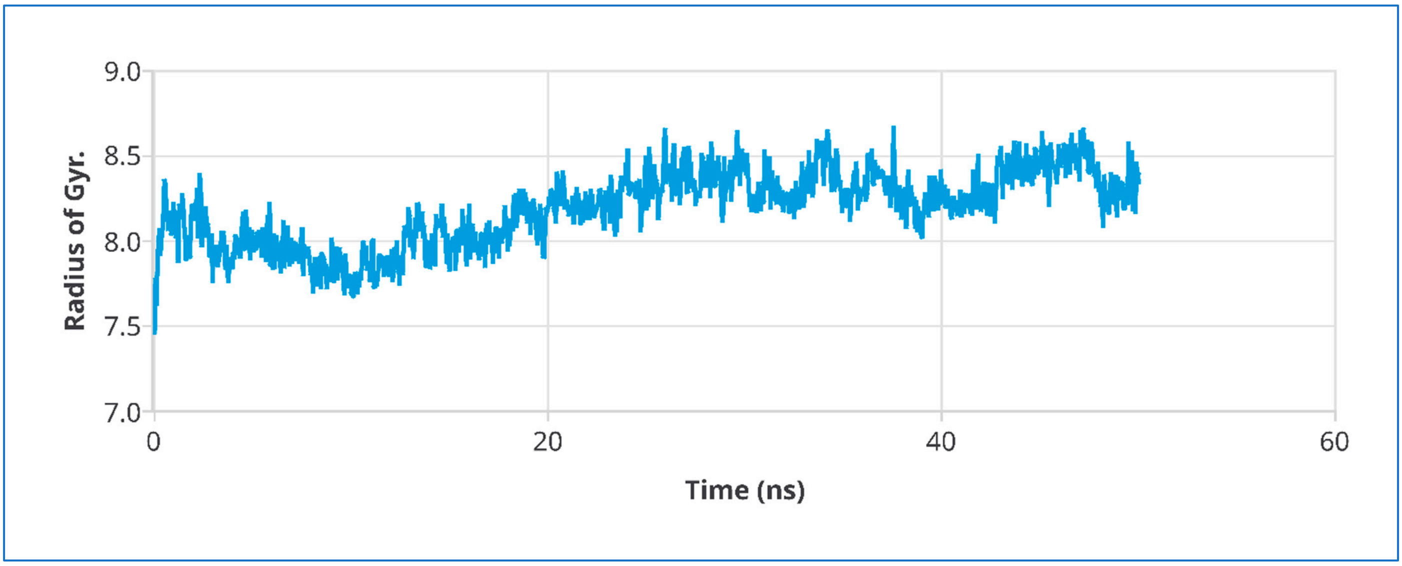

Molecular Surface Area

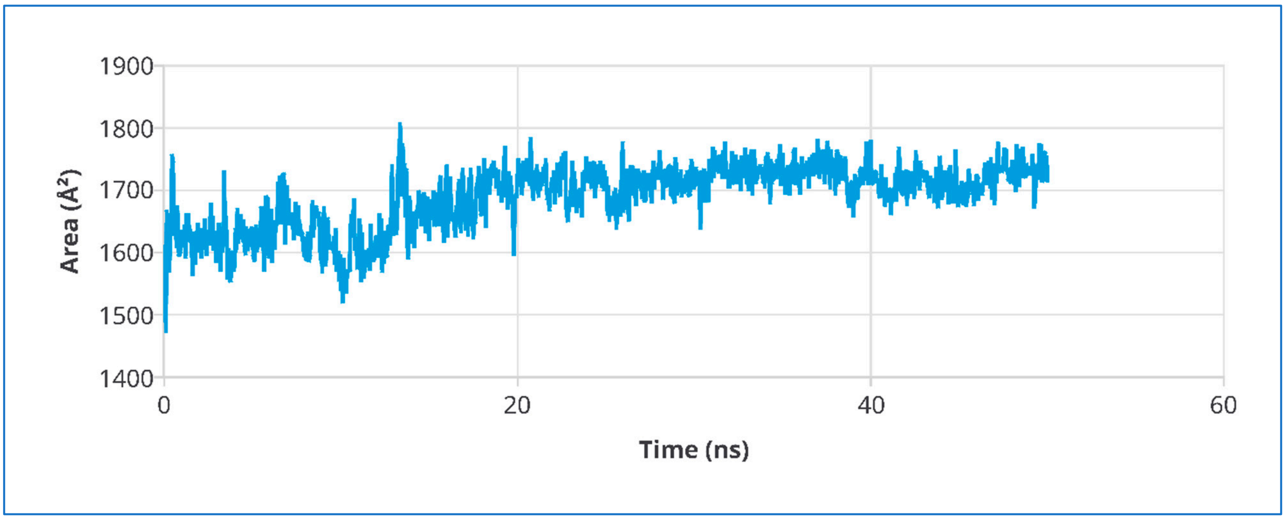

Polar Surface Area

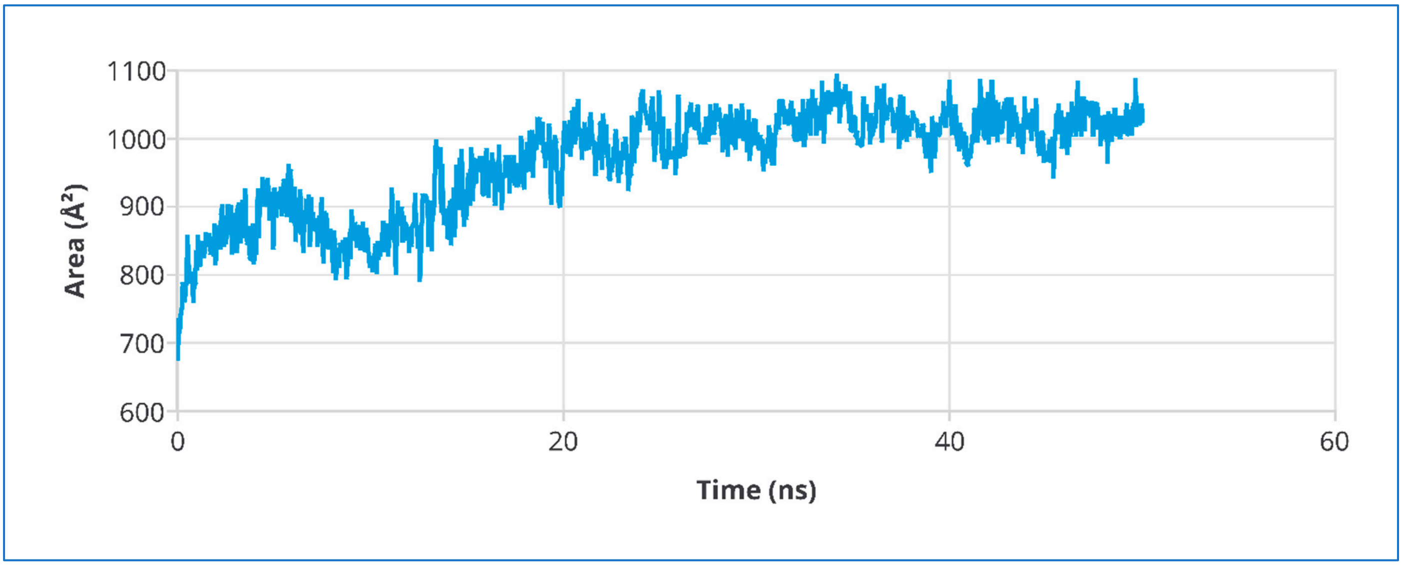

Radial Distribution Function (RDF)

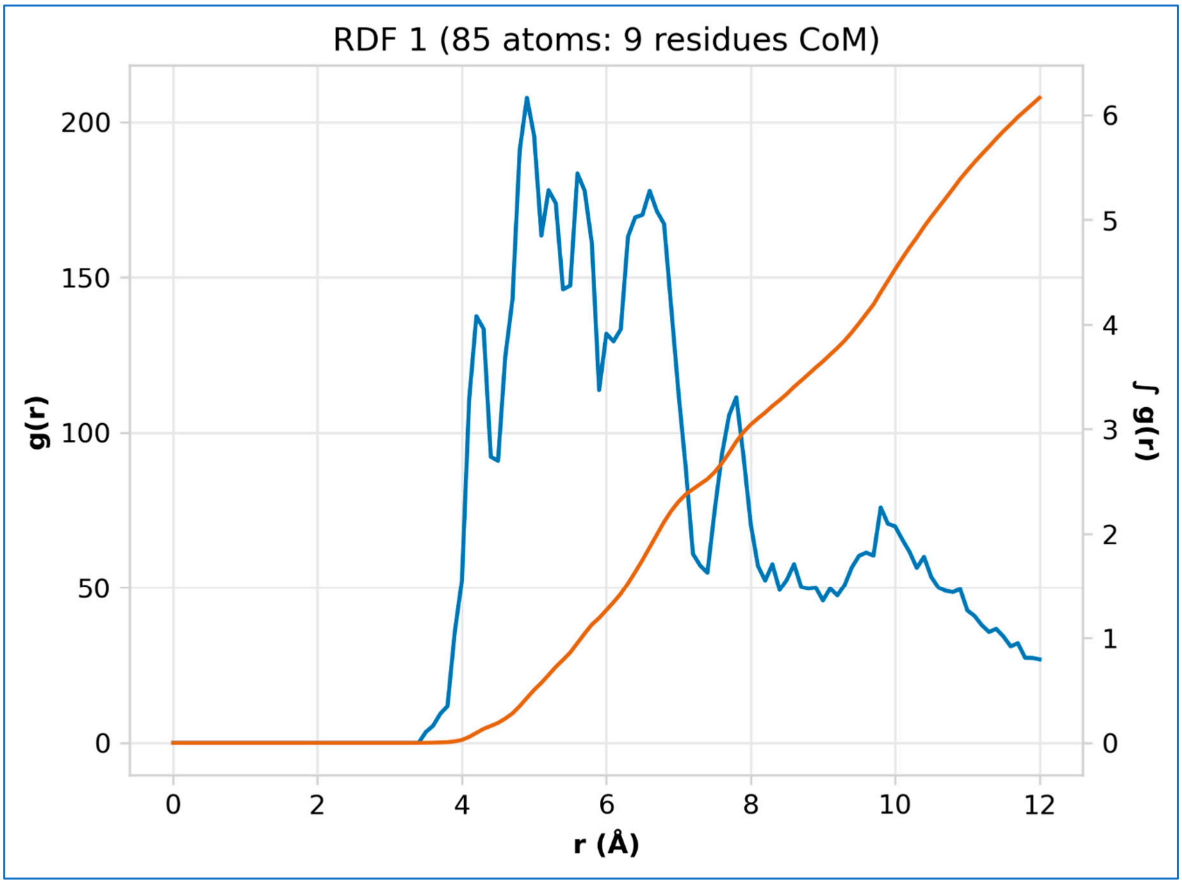

Solvent accessible surface area

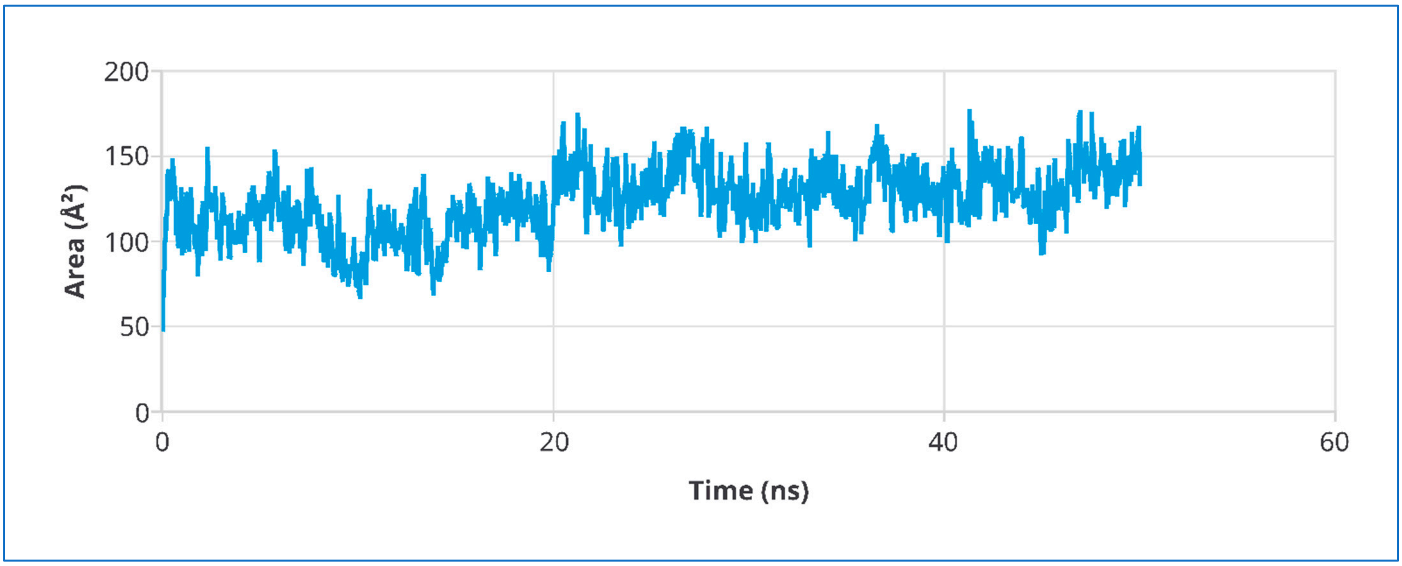

Molecular dynamics between methyl eugenol and target 5X29 protein of SARS-CoV-2.

Results

Interaction Counts

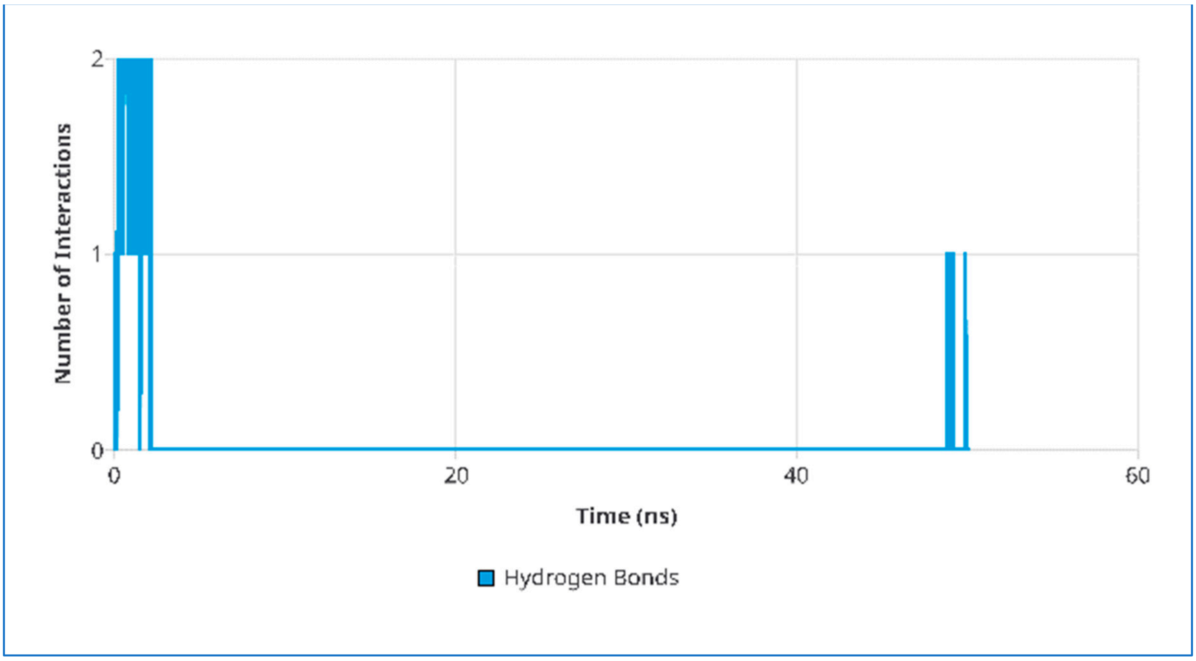

RMSD

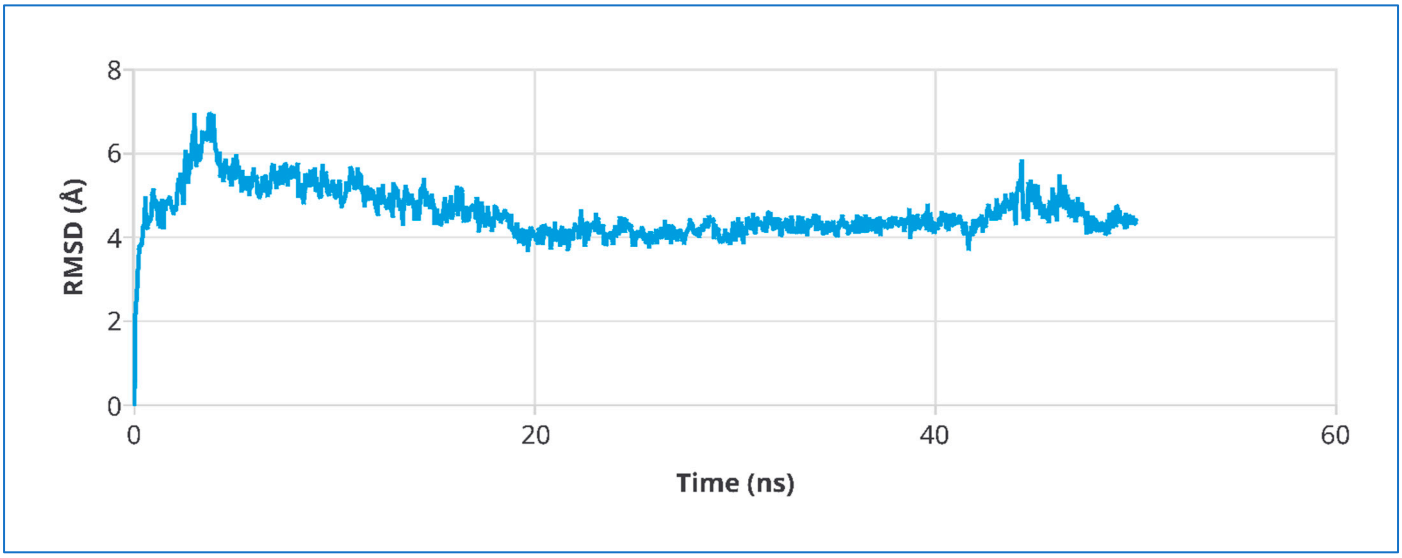

RMSF

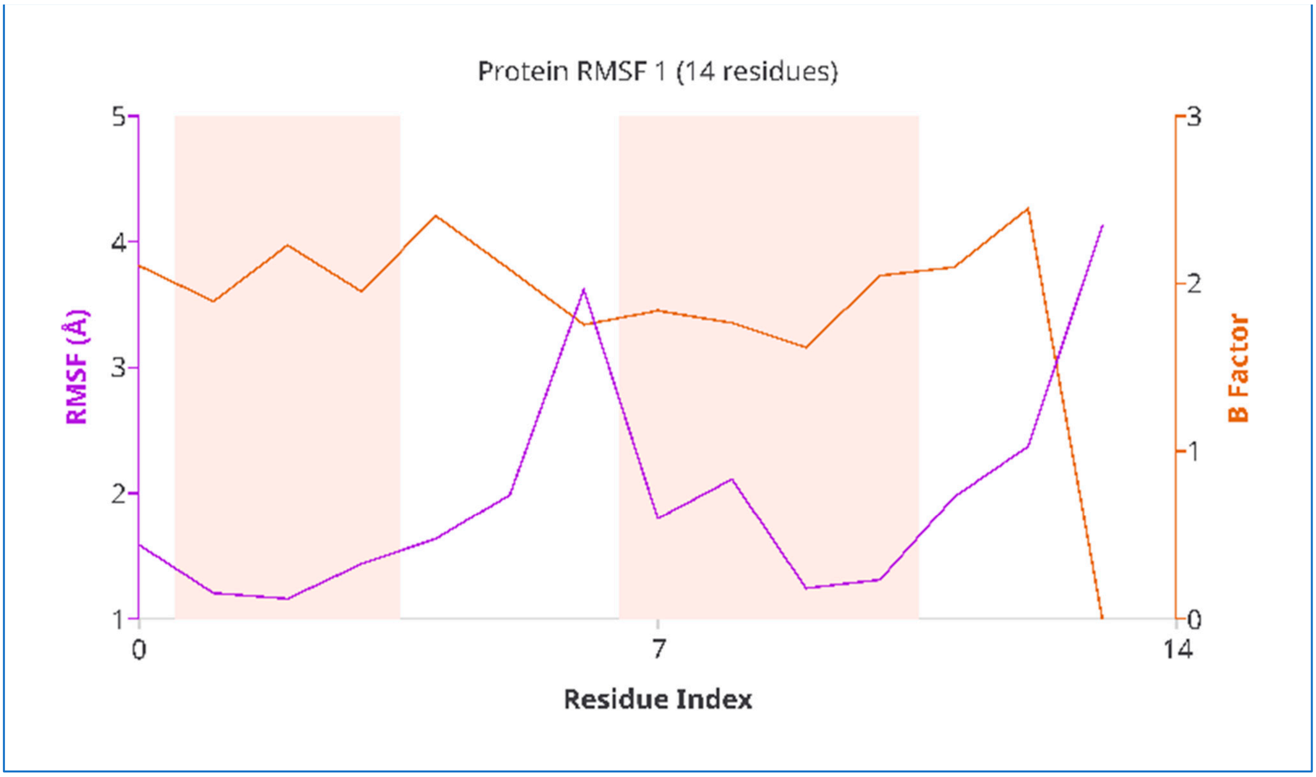

Radius of Gyration

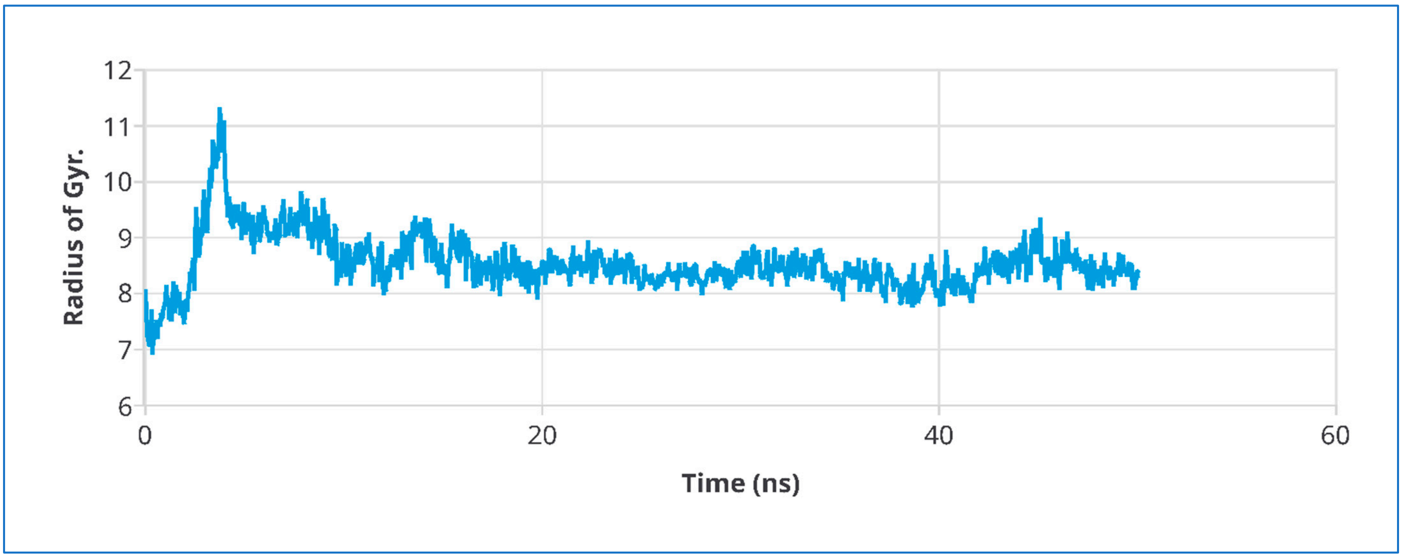

Molecular Surface Area

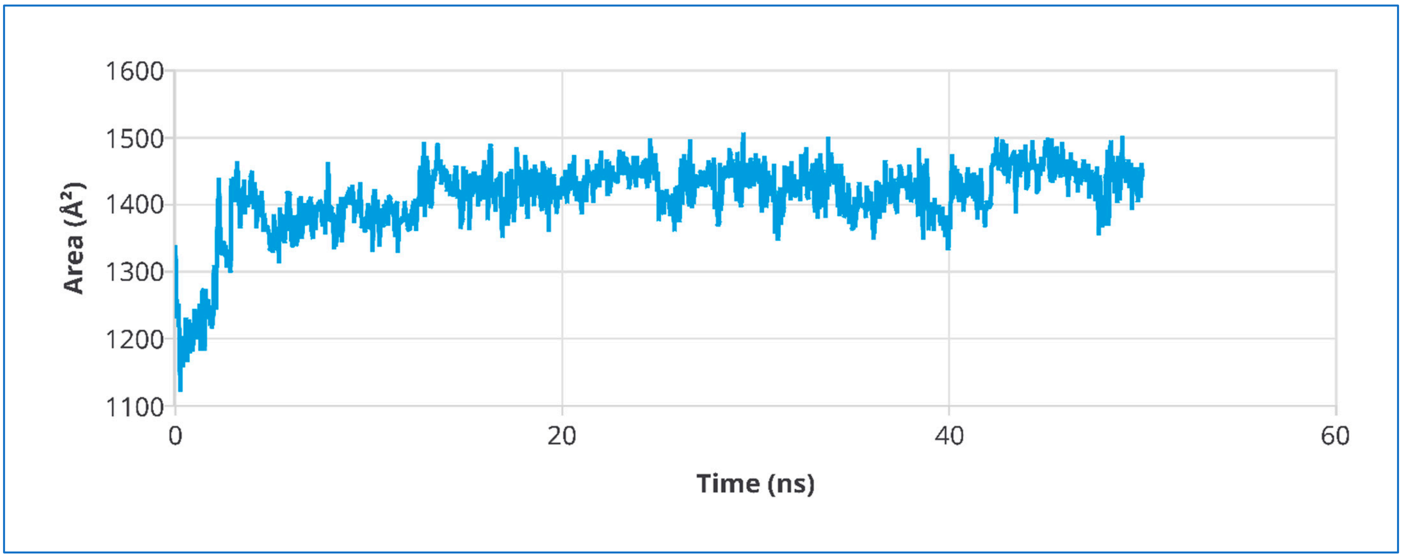

Polar Surface Area

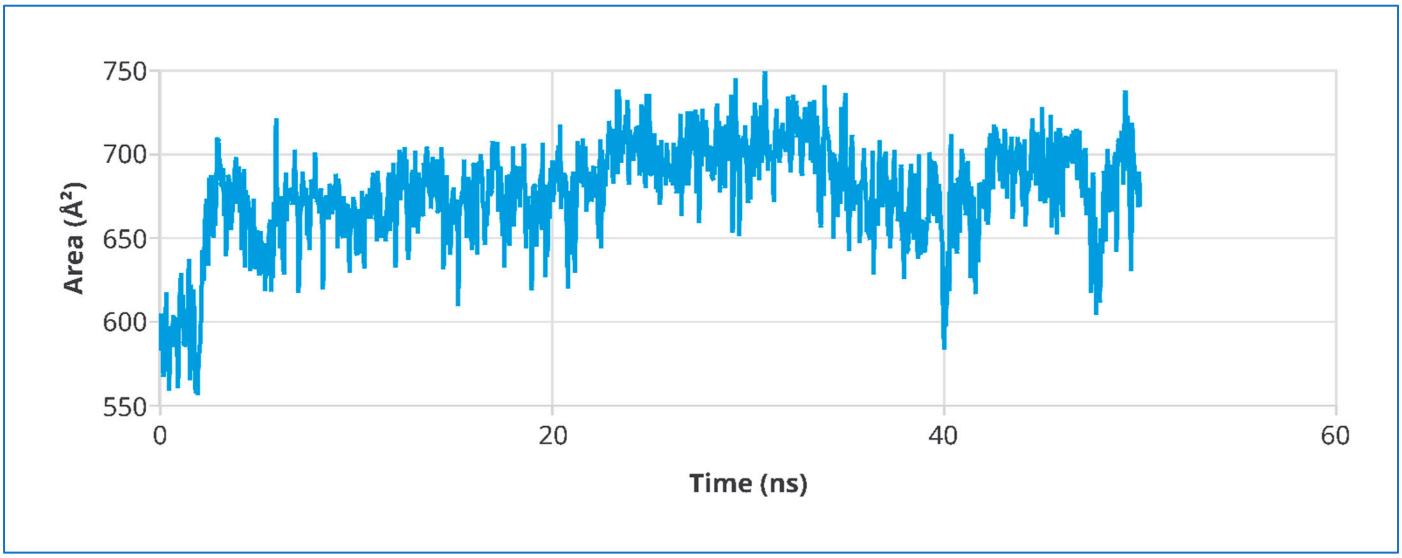

Radial Distribution Function (RDF)

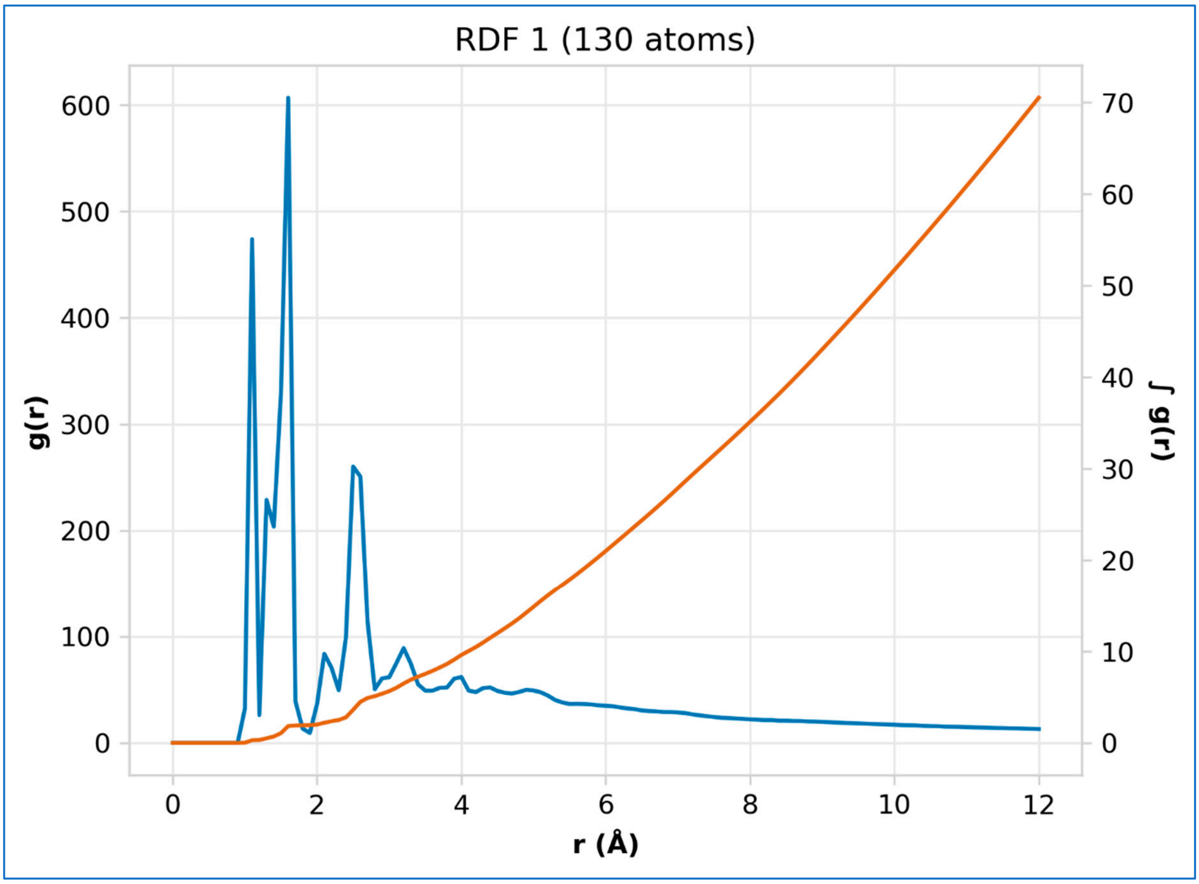

Solvent accessible surface area

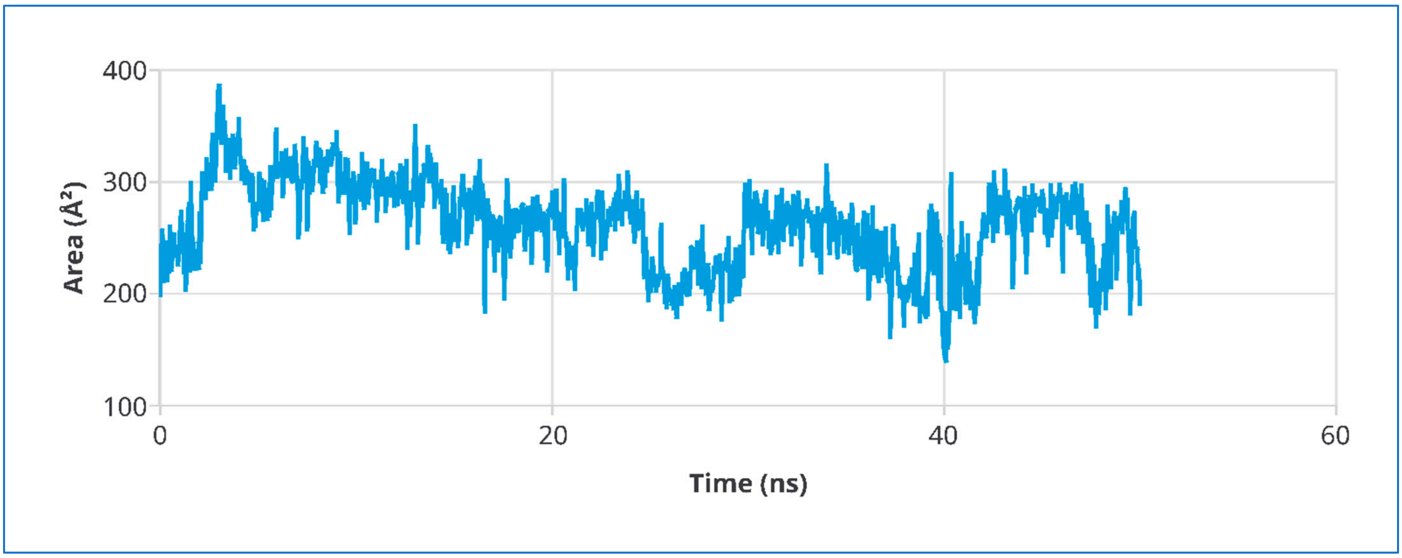

Results

Interaction Counts

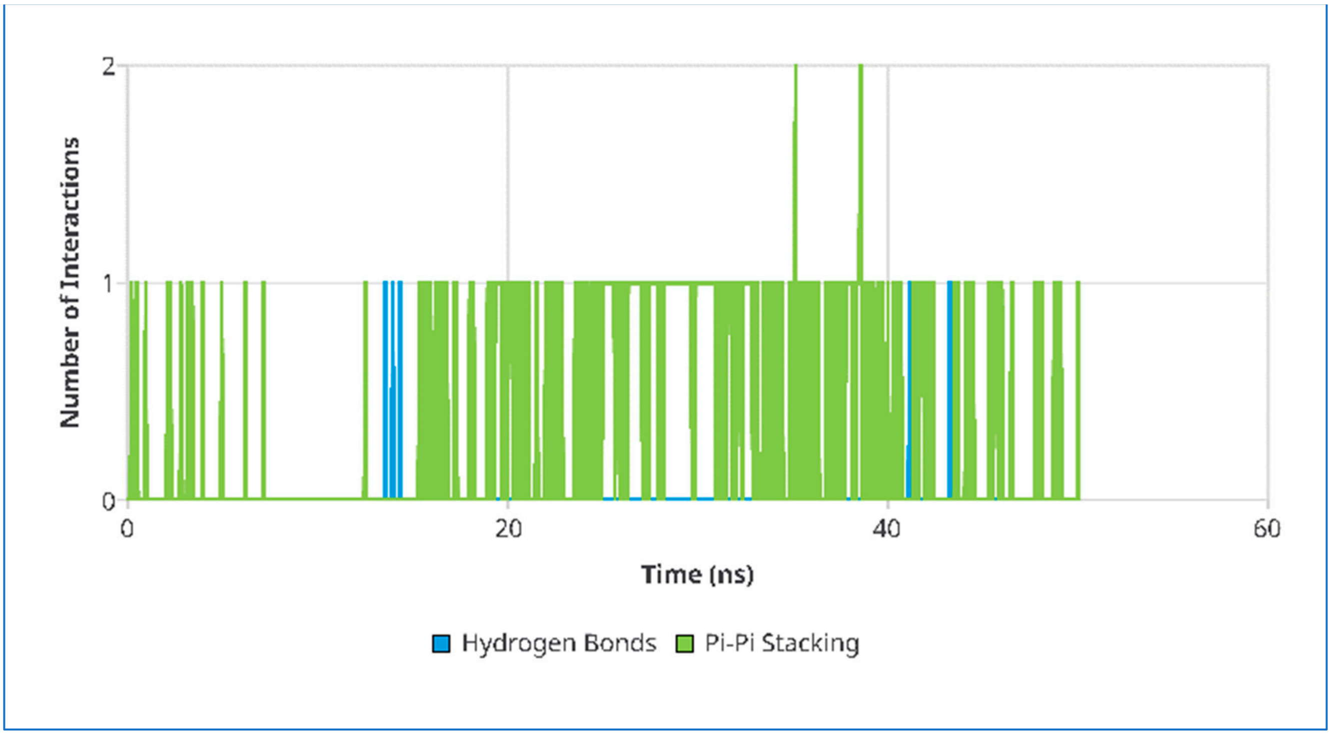

RMSD

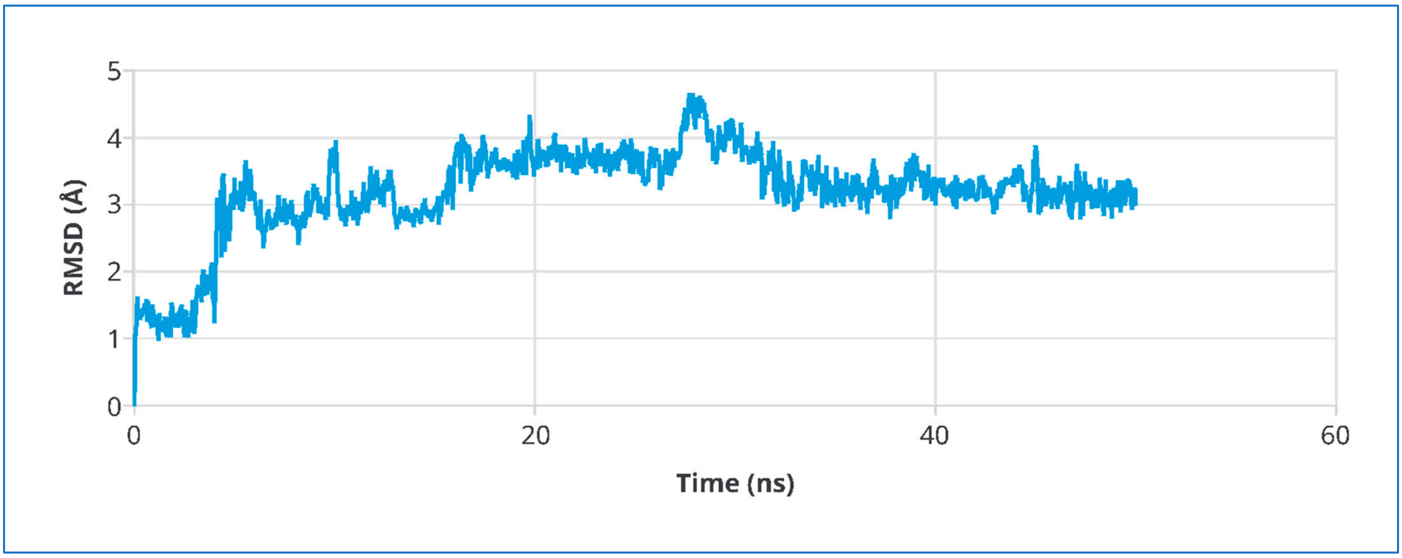

RMSF

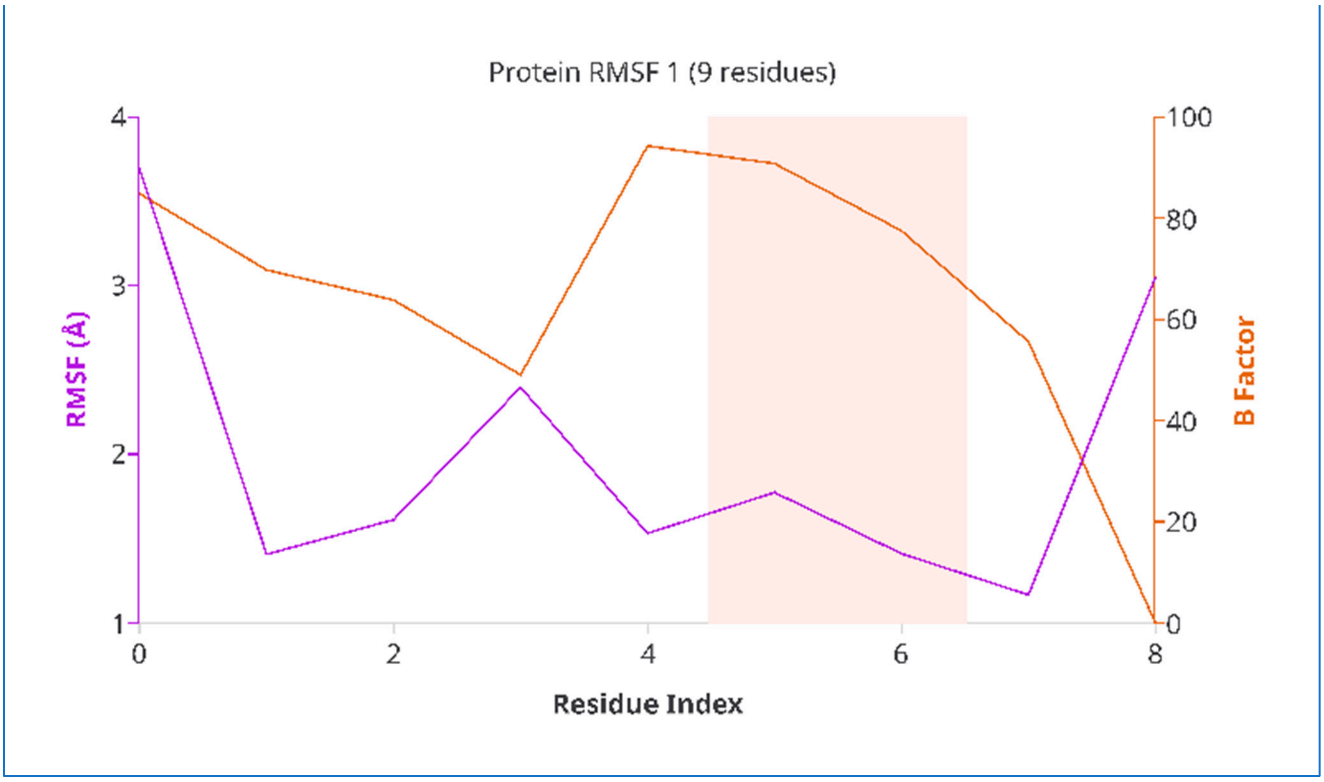

Radius of Gyration

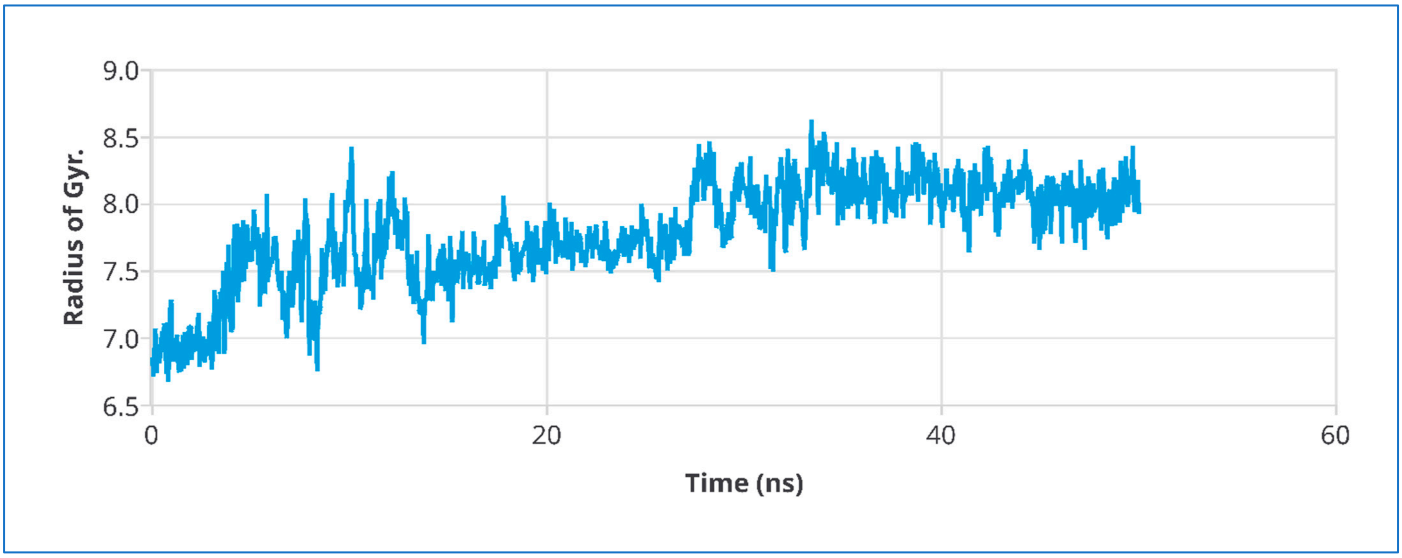

Molecular Surface Area

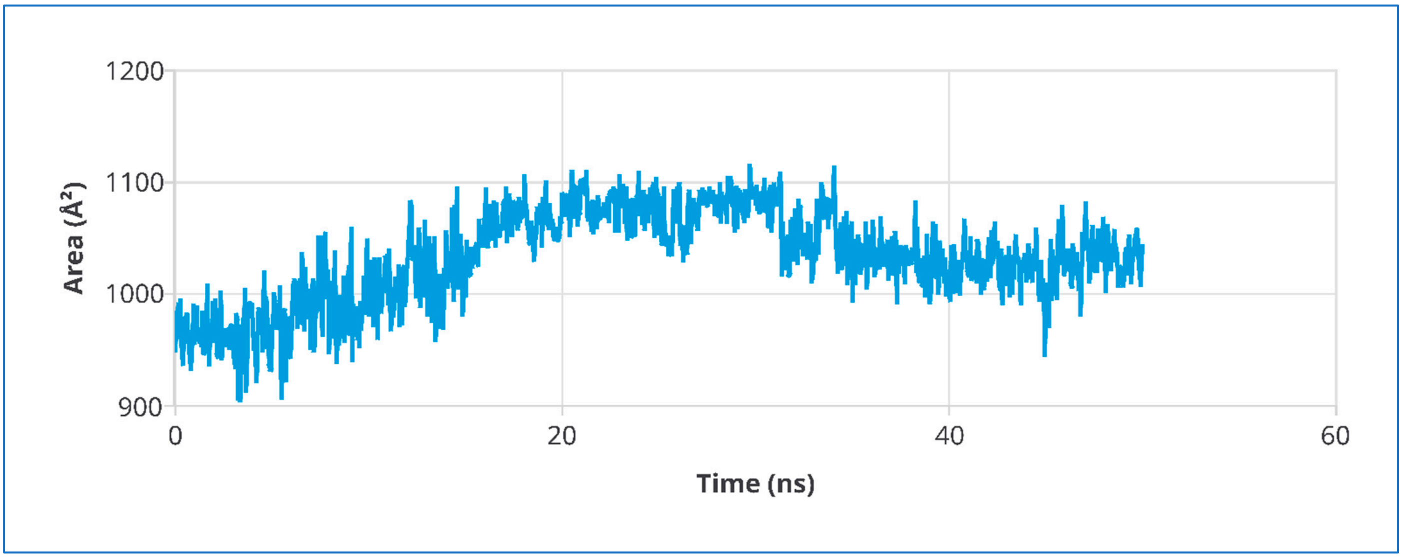

Polar Surface Area

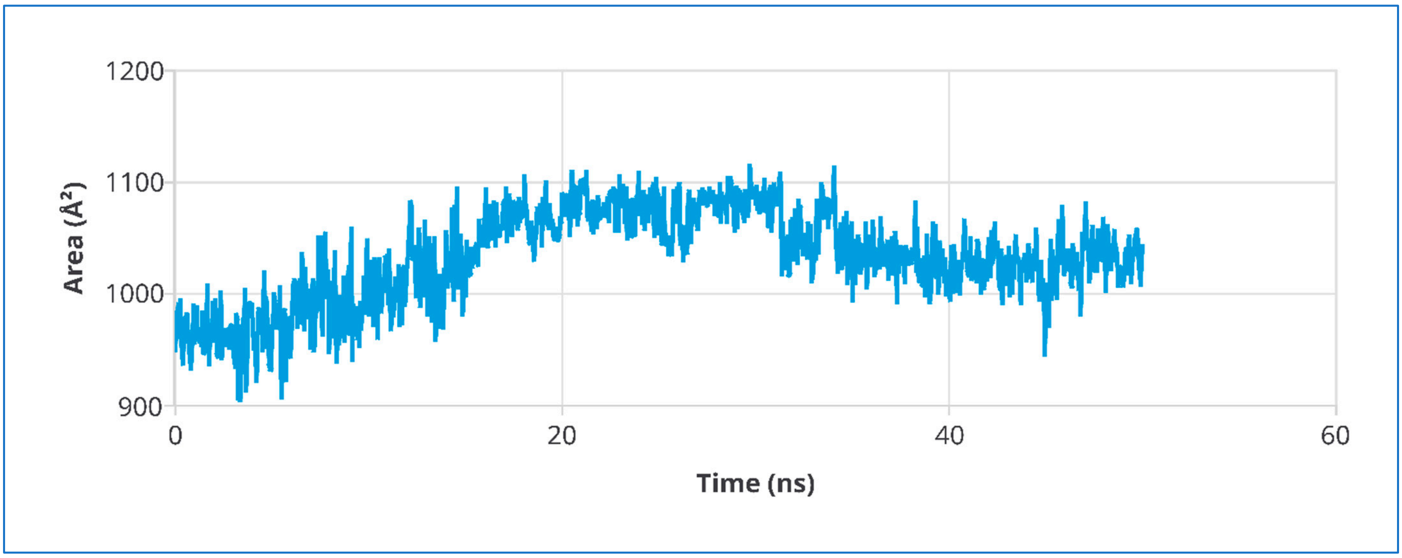

Radial Distribution Function (RDF)

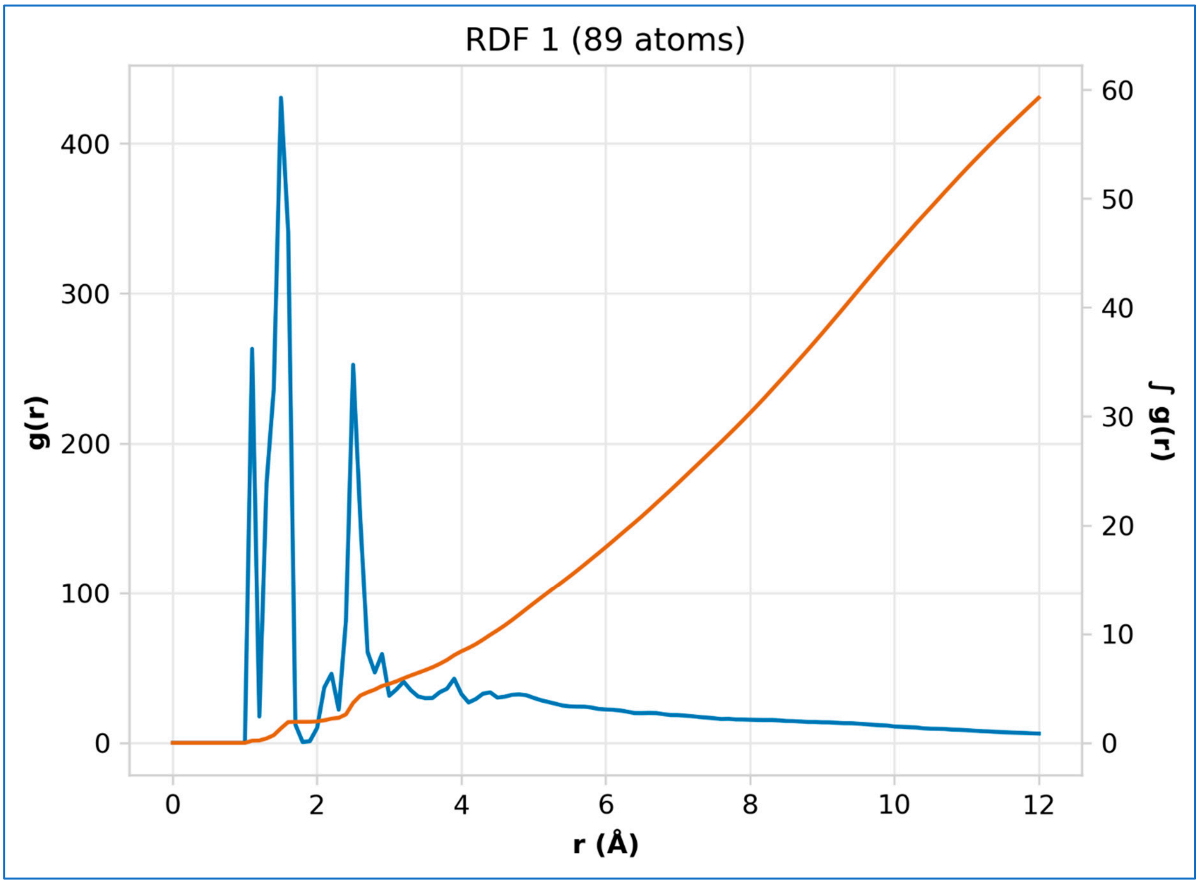

Solvent accessible surface area

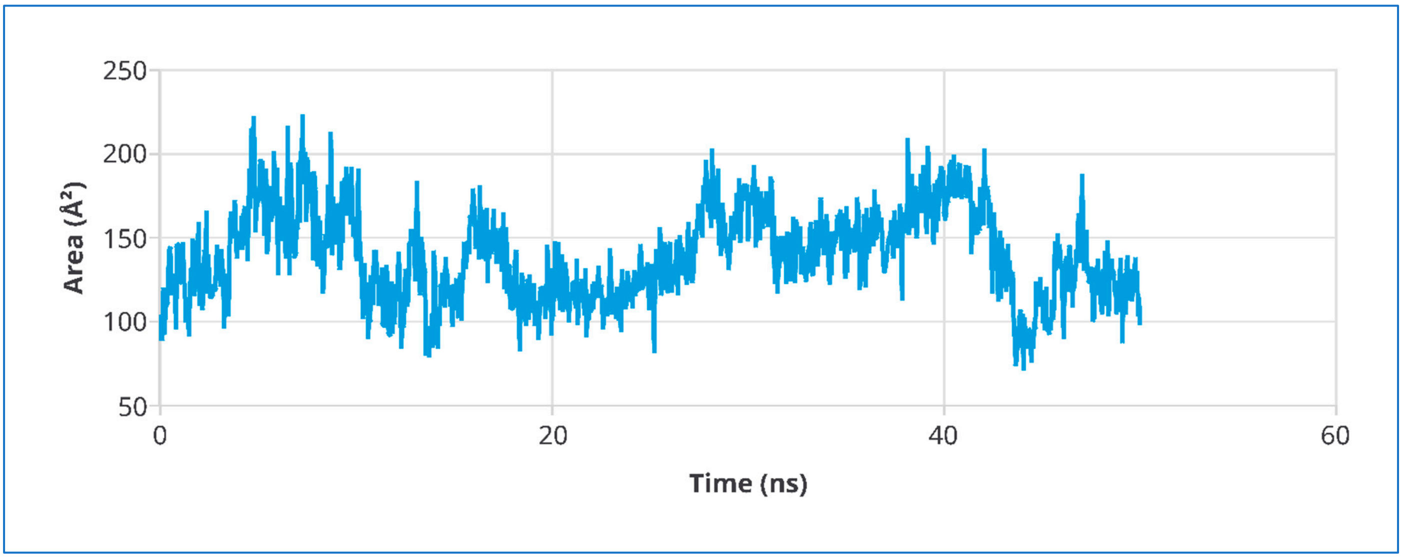

Results

Interaction Counts

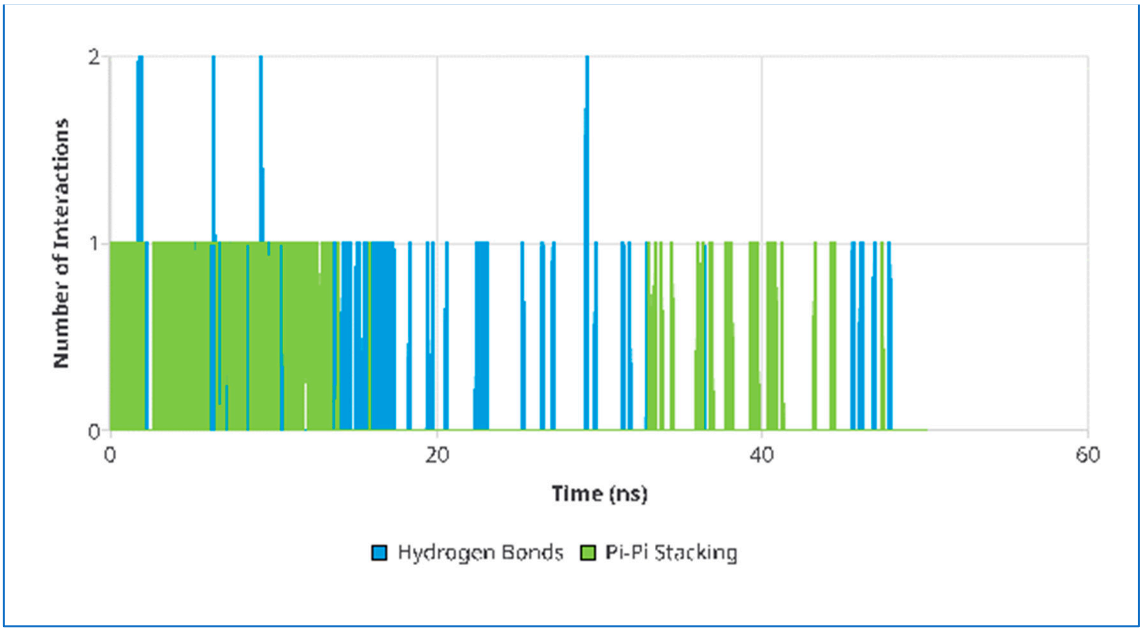

RMSD

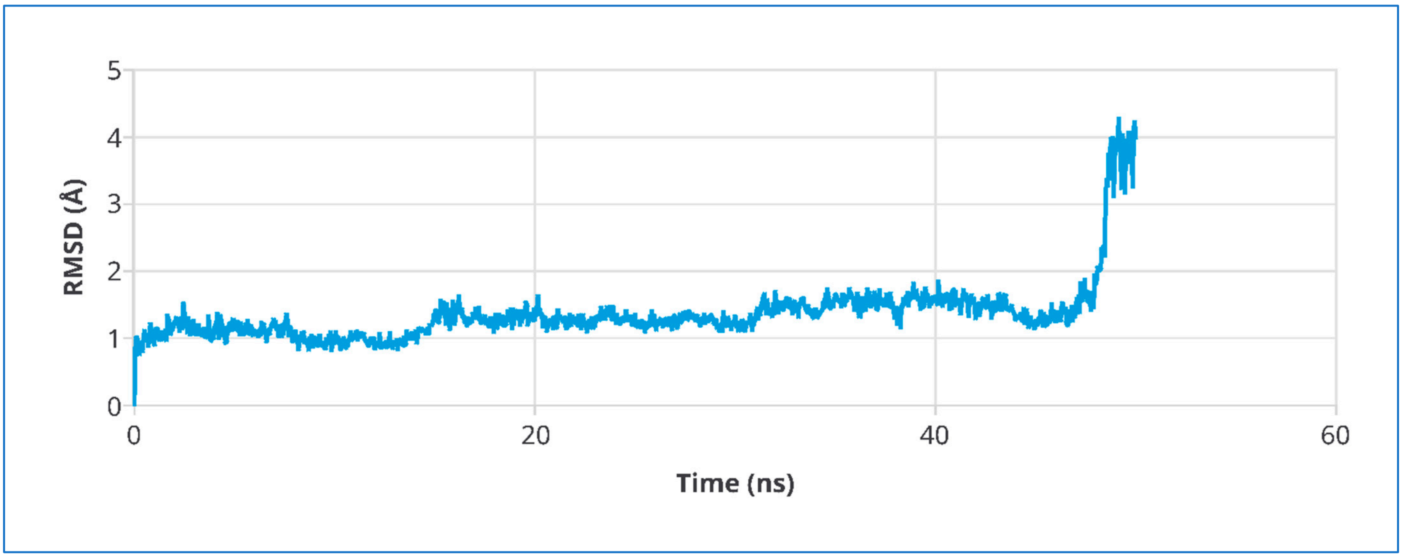

RMSF

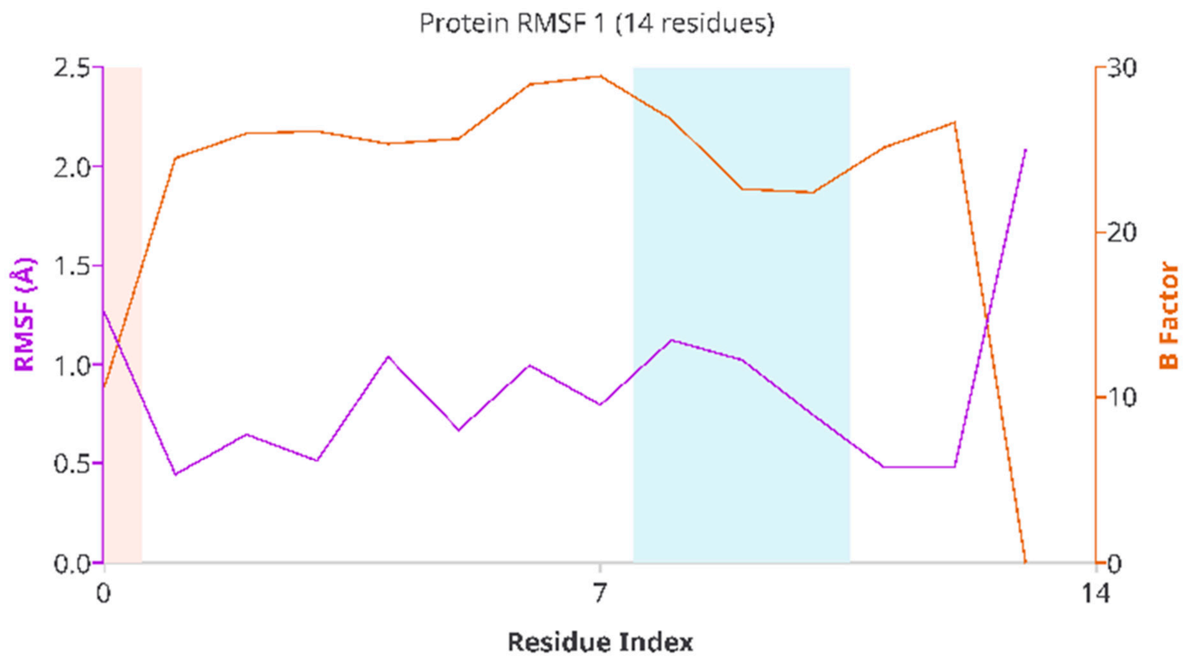

Radius of Gyration

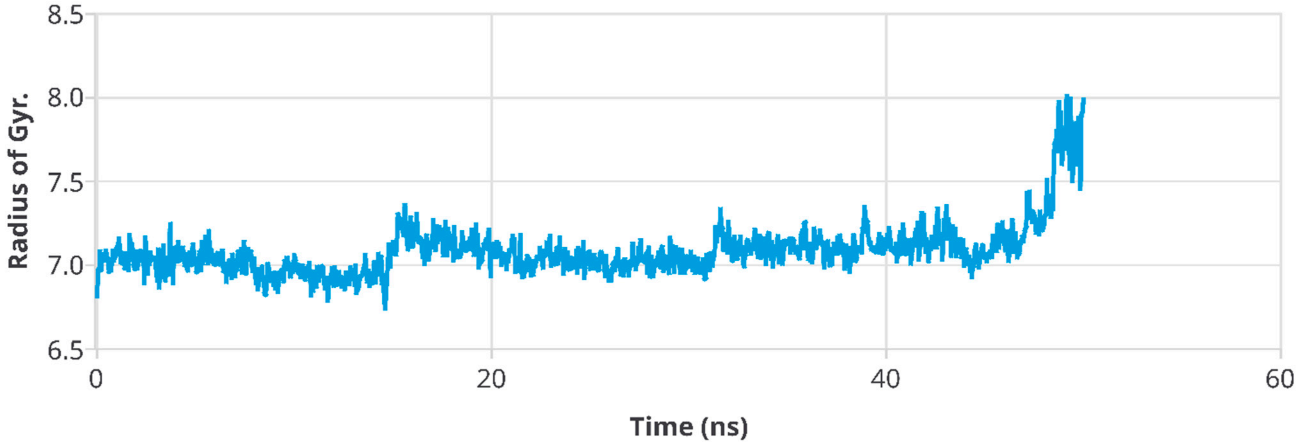

Molecular Surface Area

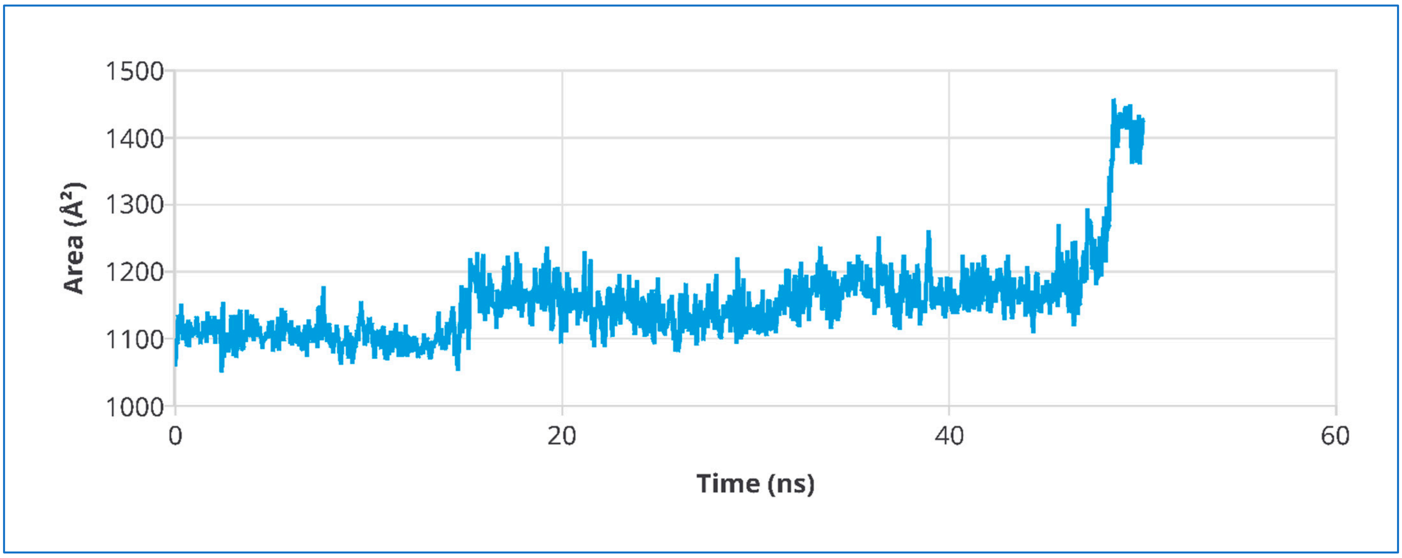

Polar Surface Area

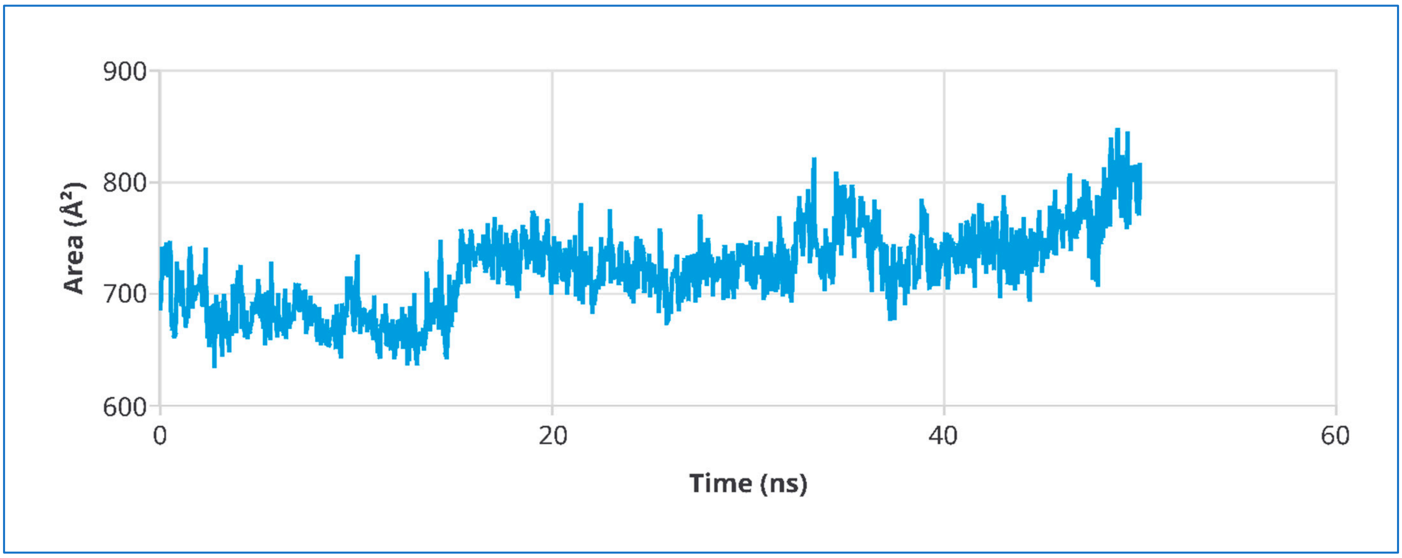

Radial Distribution Function (RDF)

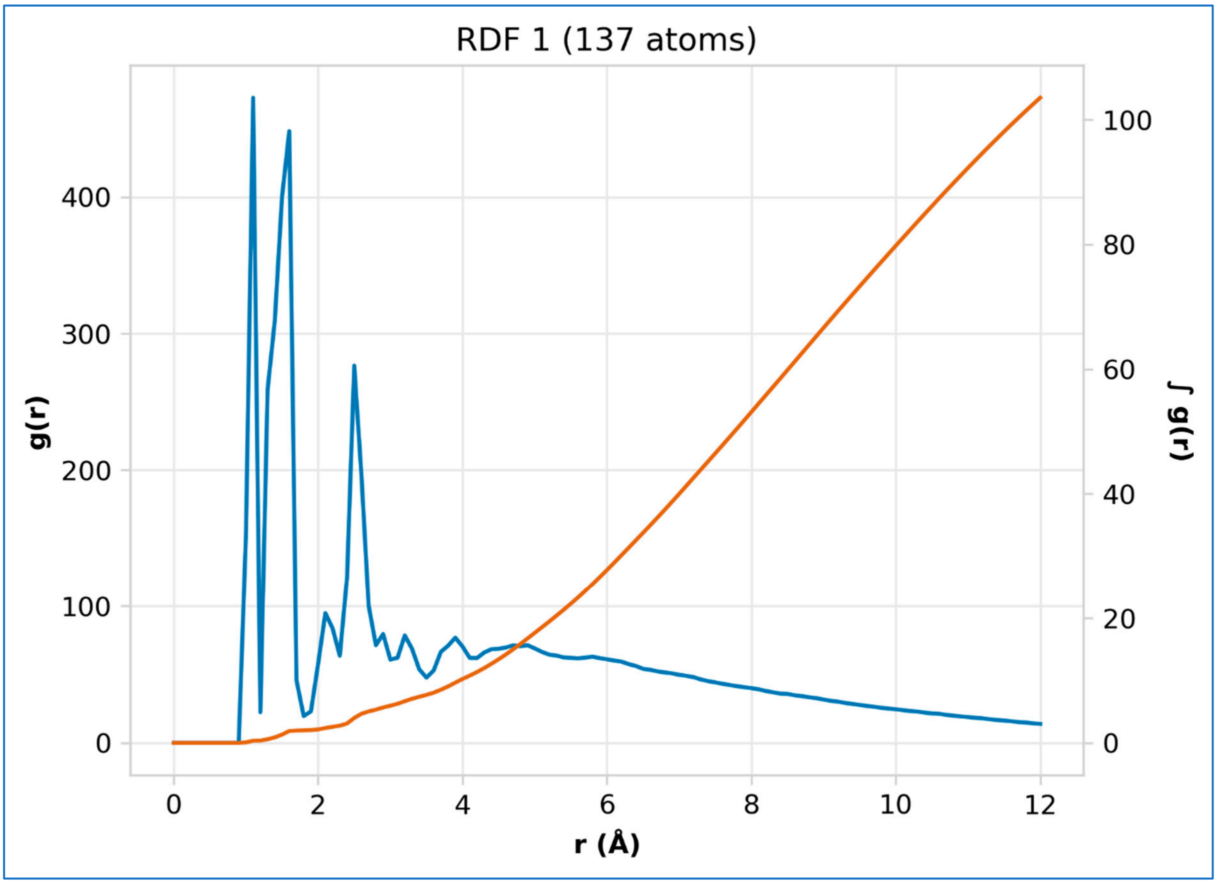

Solvent accessible surface area

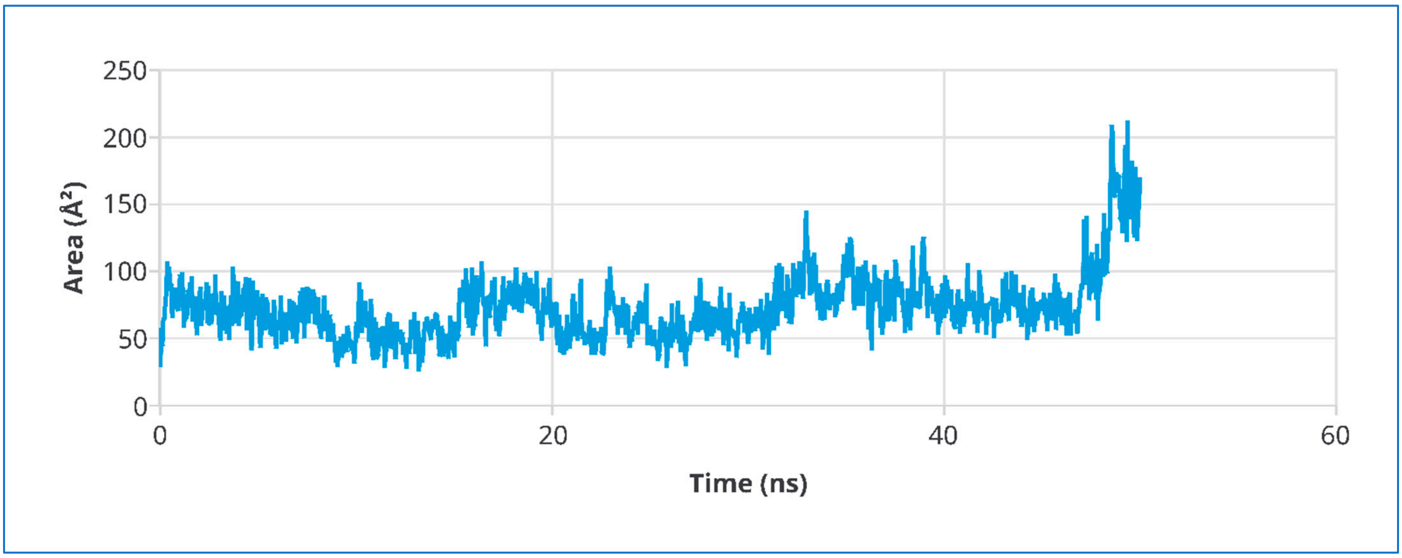

Results

Interaction Counts

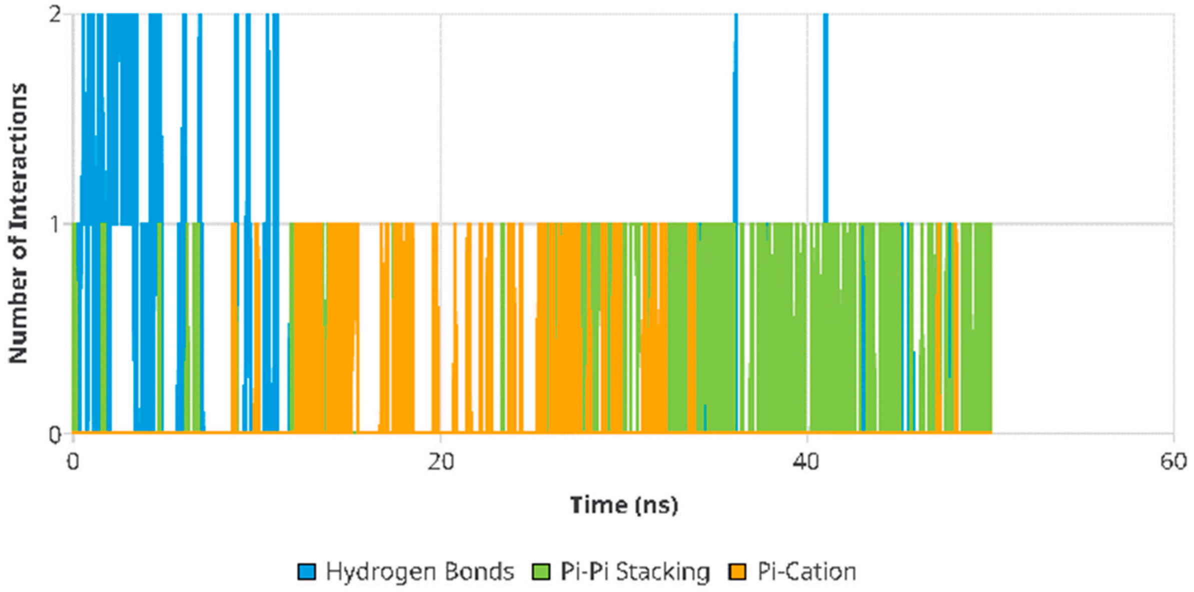

RMSD

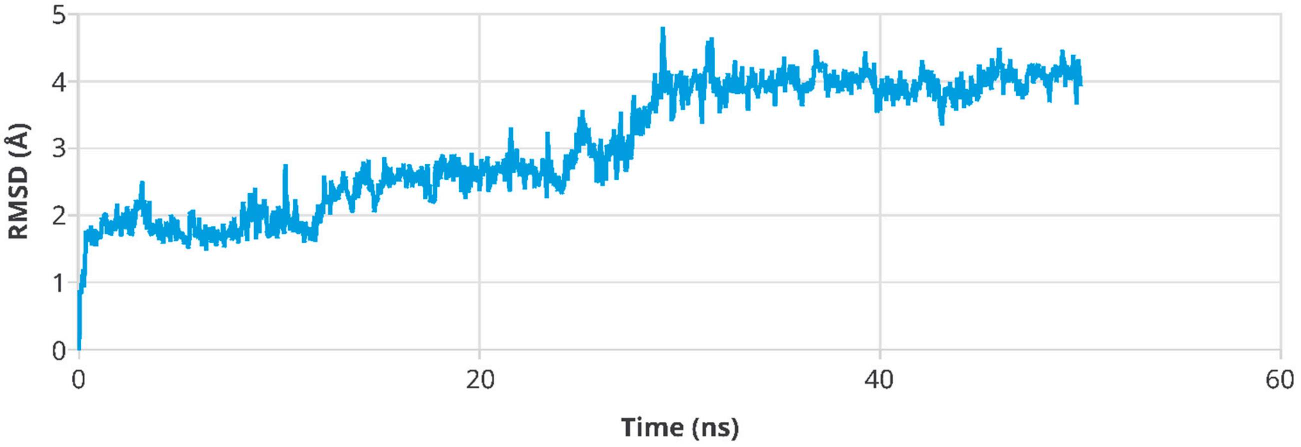

RMSF

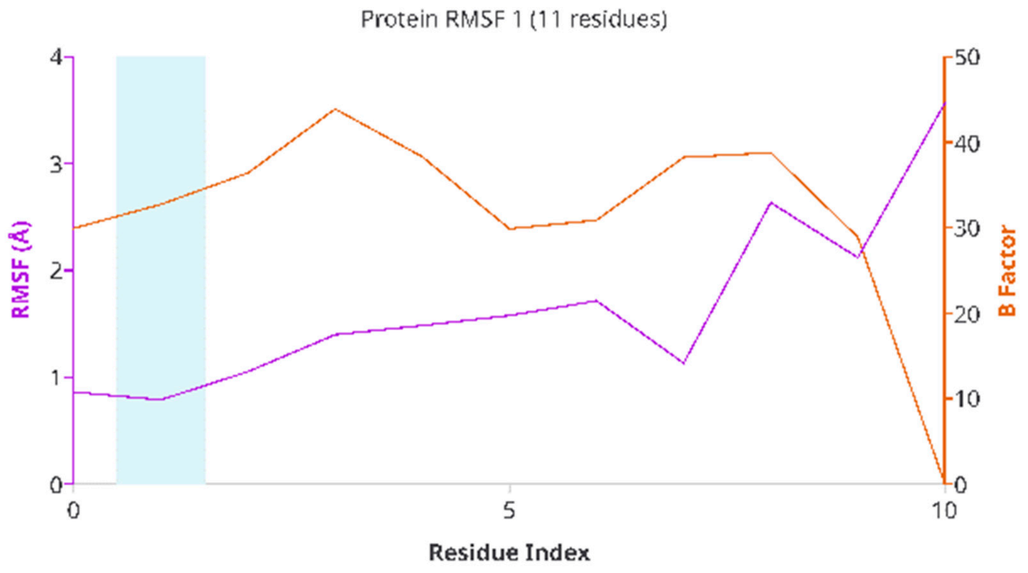

Radius of Gyration

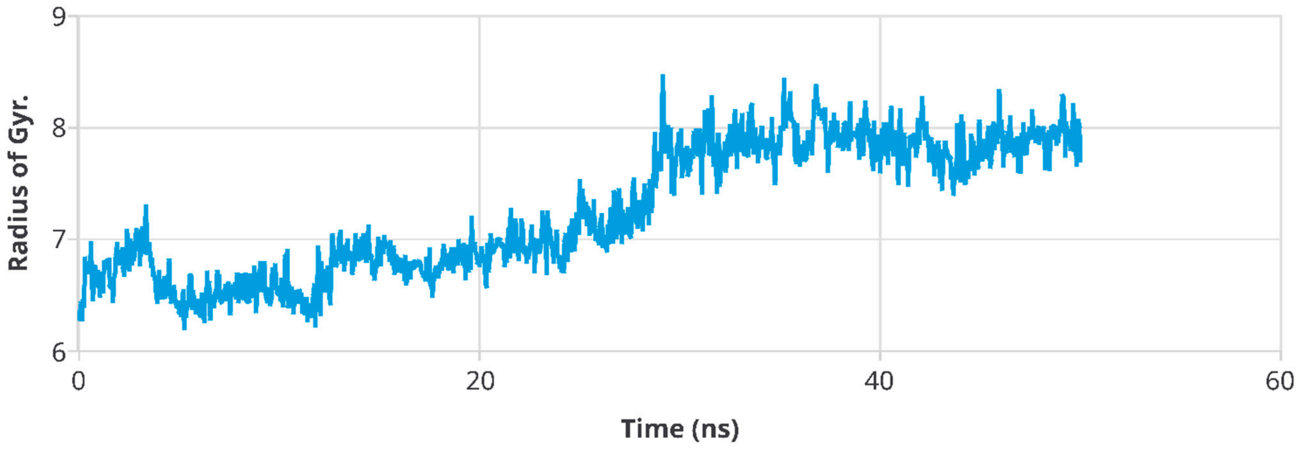

Molecular Surface Area

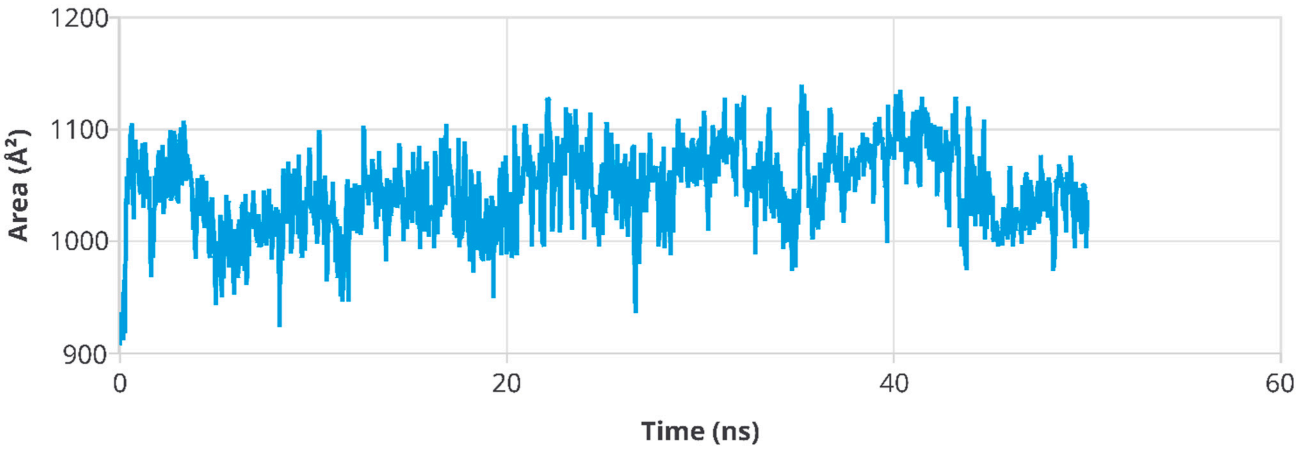

Polar Surface Area

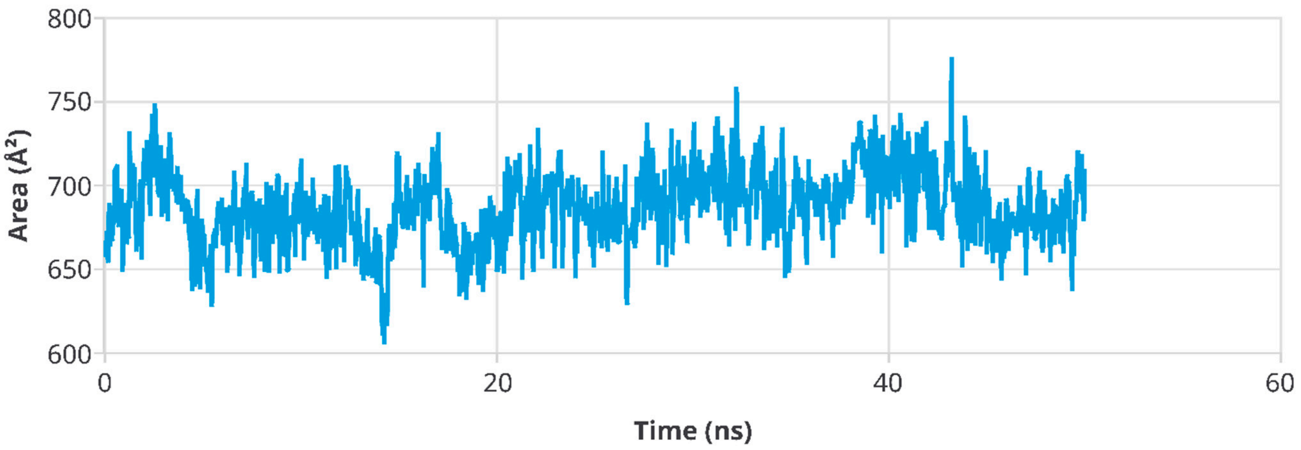

Radial Distribution Function (RDF)

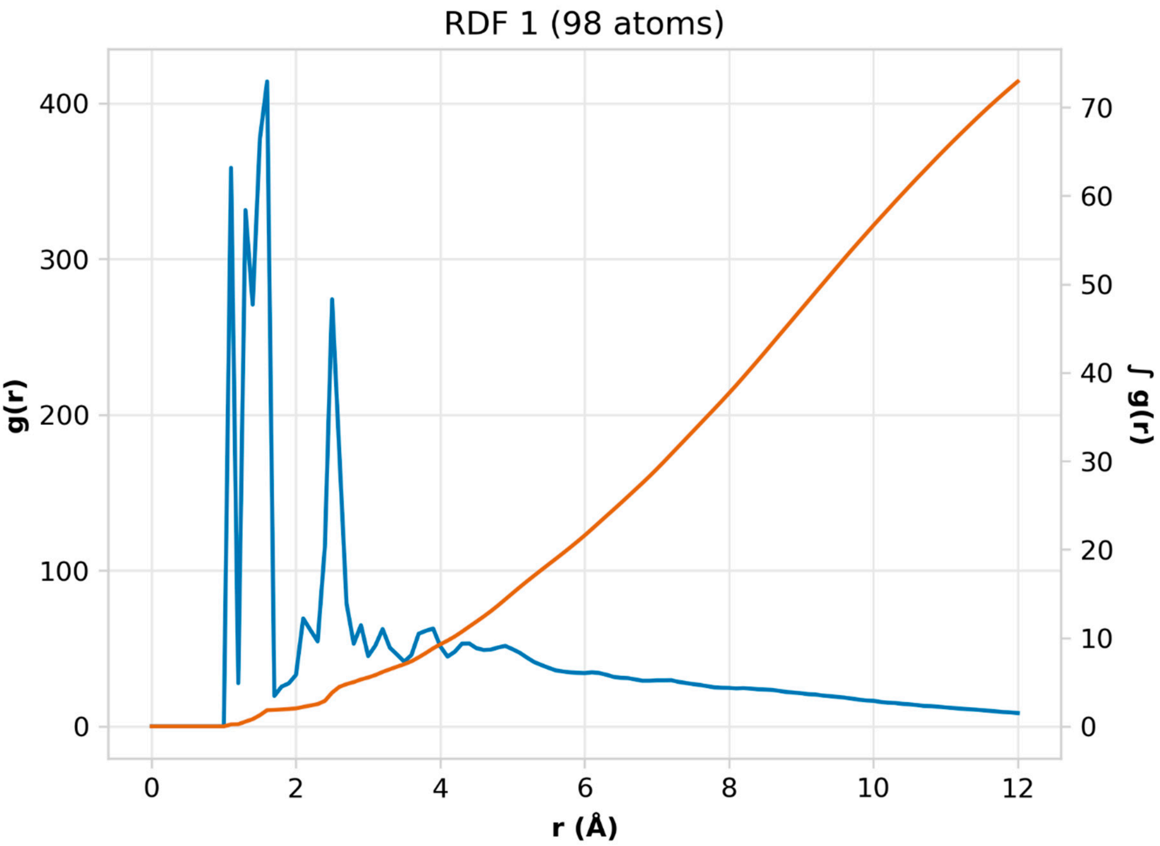

Solvent accessible surface area

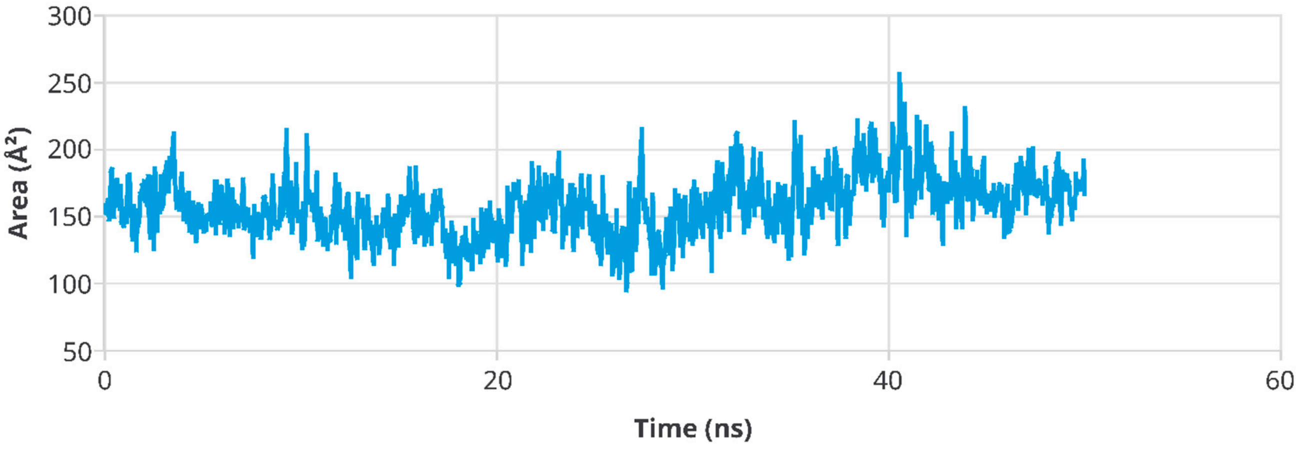

300
250
200
150
100
50
Area (Å²)
0
20
40
60
Time (ns)

Results

Interaction Counts

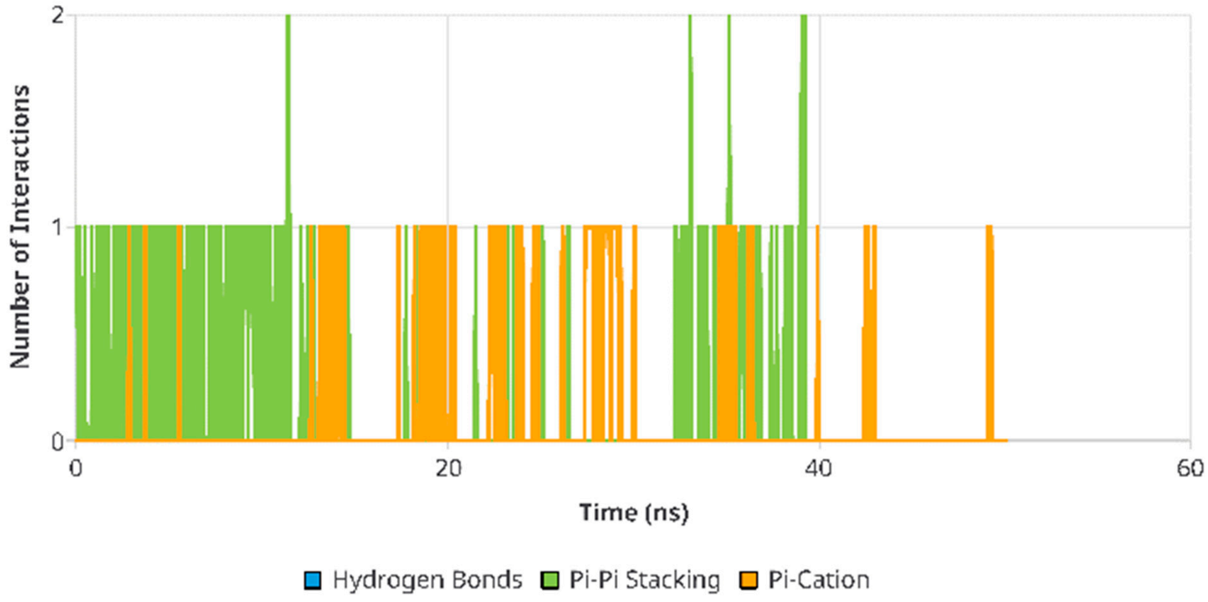

RMSD

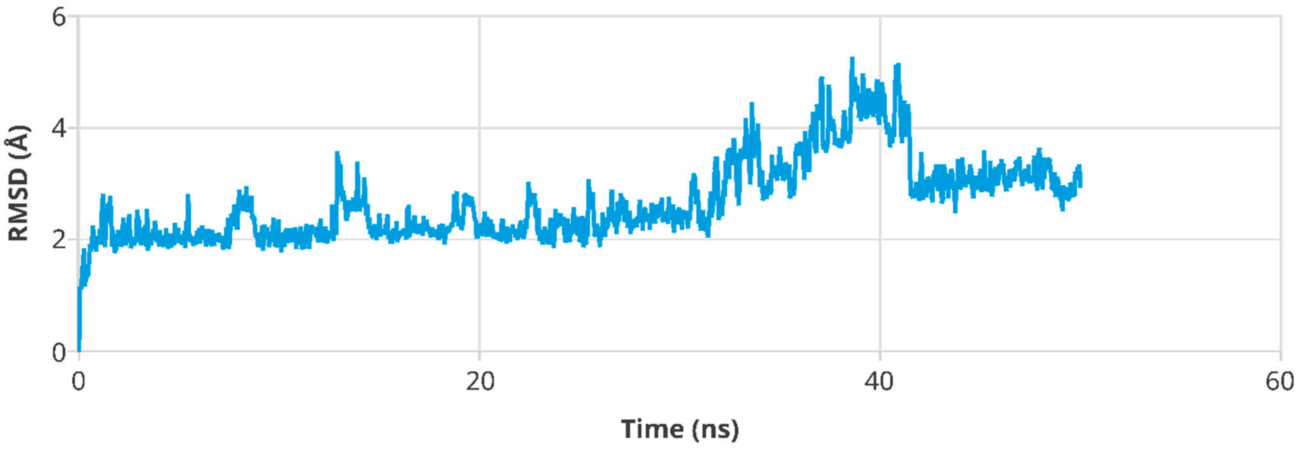

RMSF

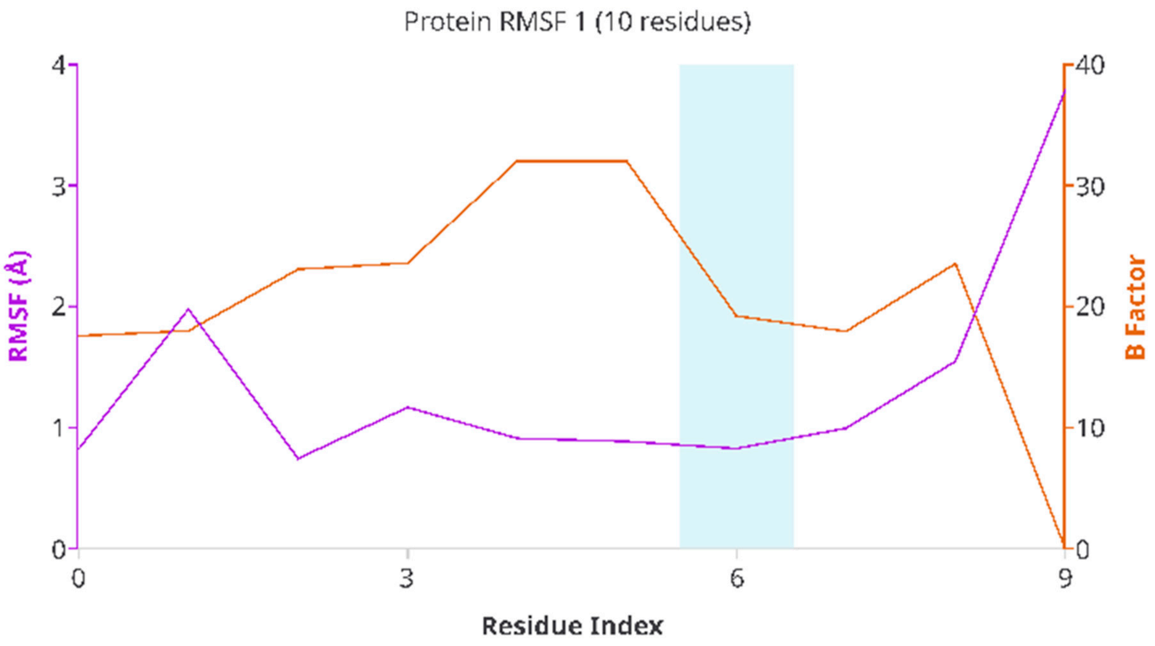

Radius of Gyration

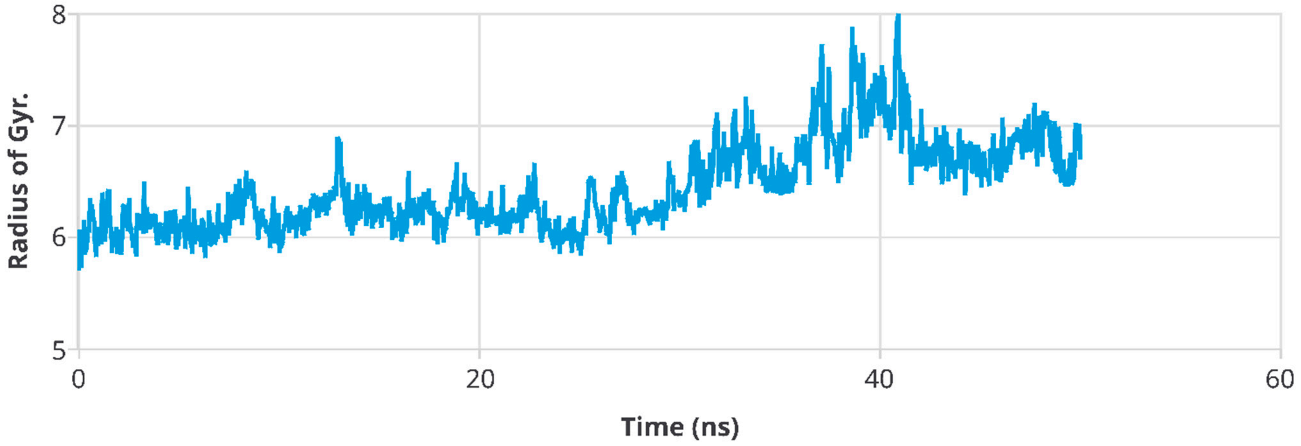

Molecular Surface Area

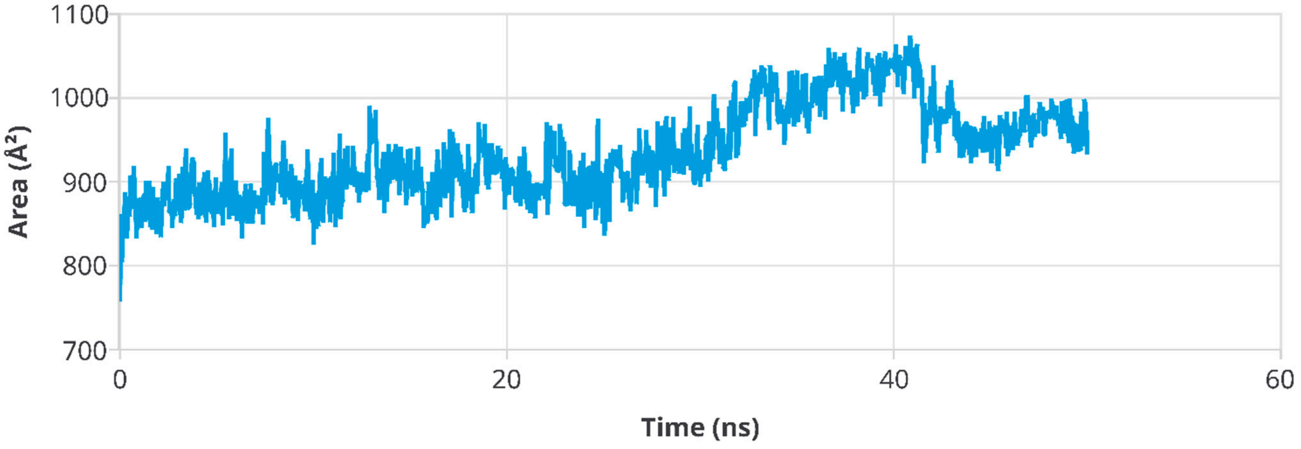

Polar Surface Area

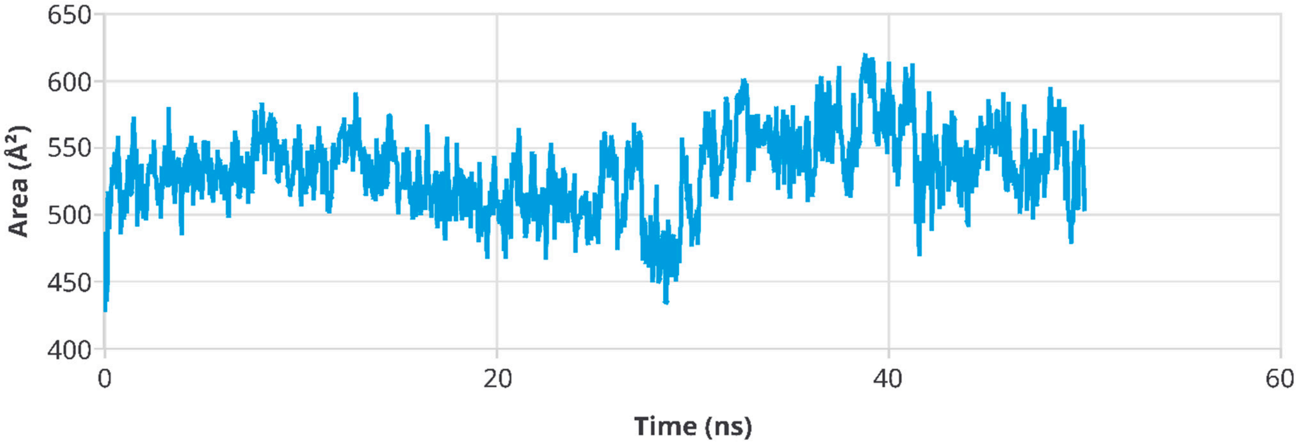

Radial Distribution Function (RDF)

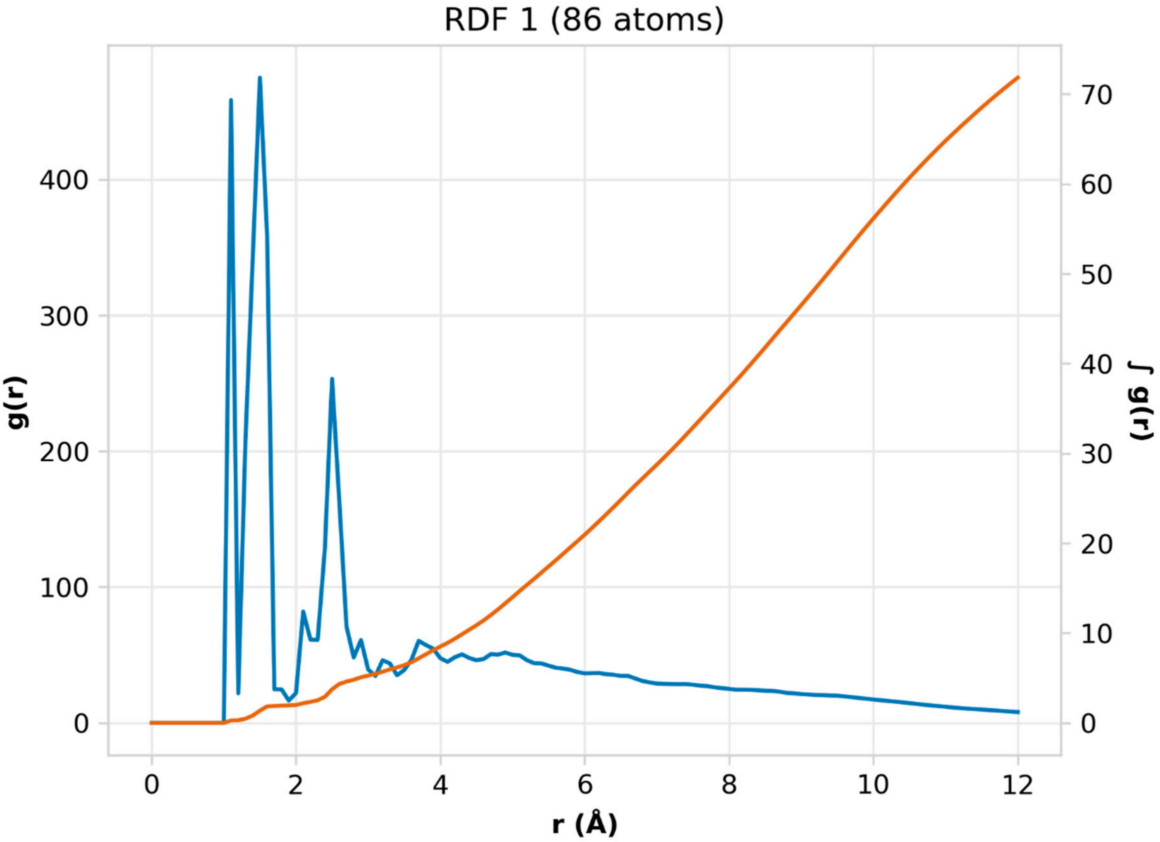

Solvent accessible surface area

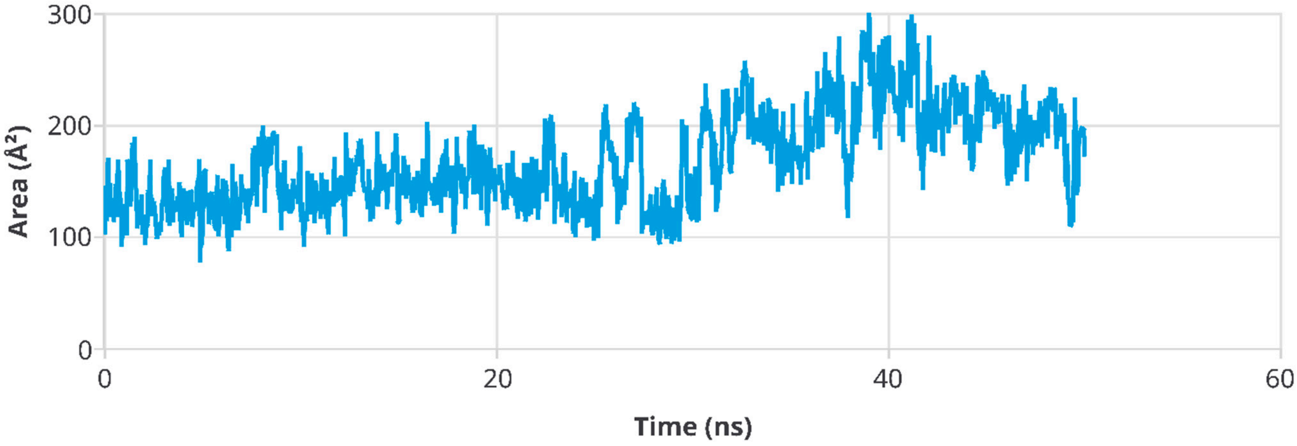

Molecular dynamics between methyl eugenol and target 7Z4S protein of SARS-CoV-2.

Results

Interaction Counts

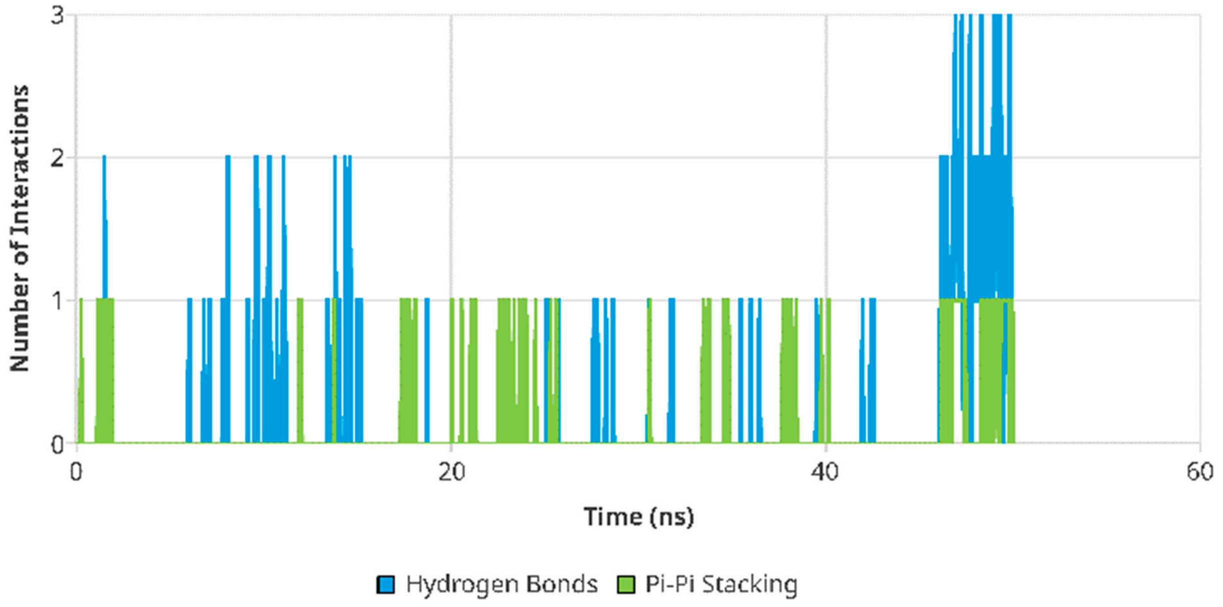

RMSD

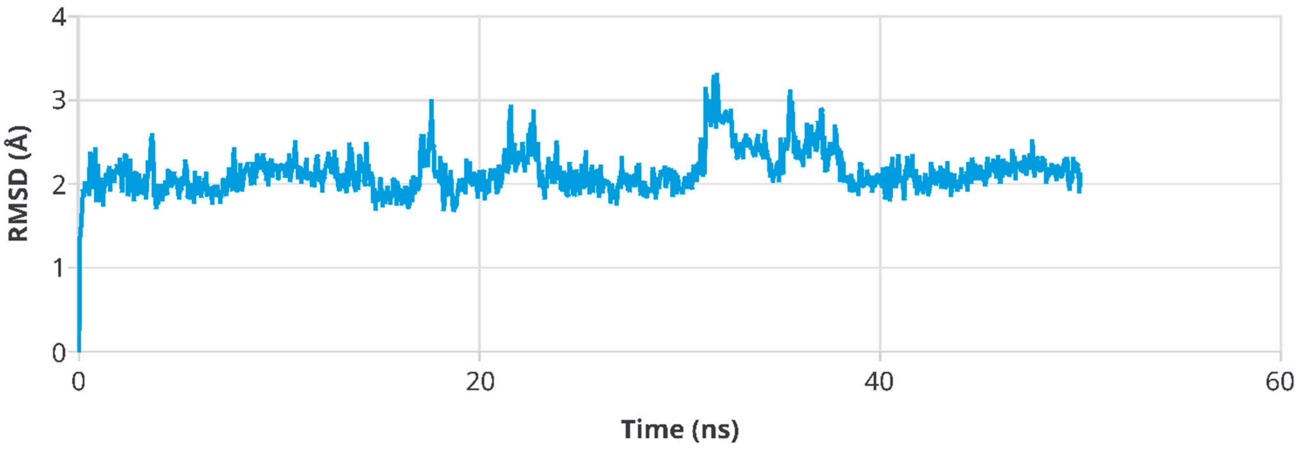

RMSF

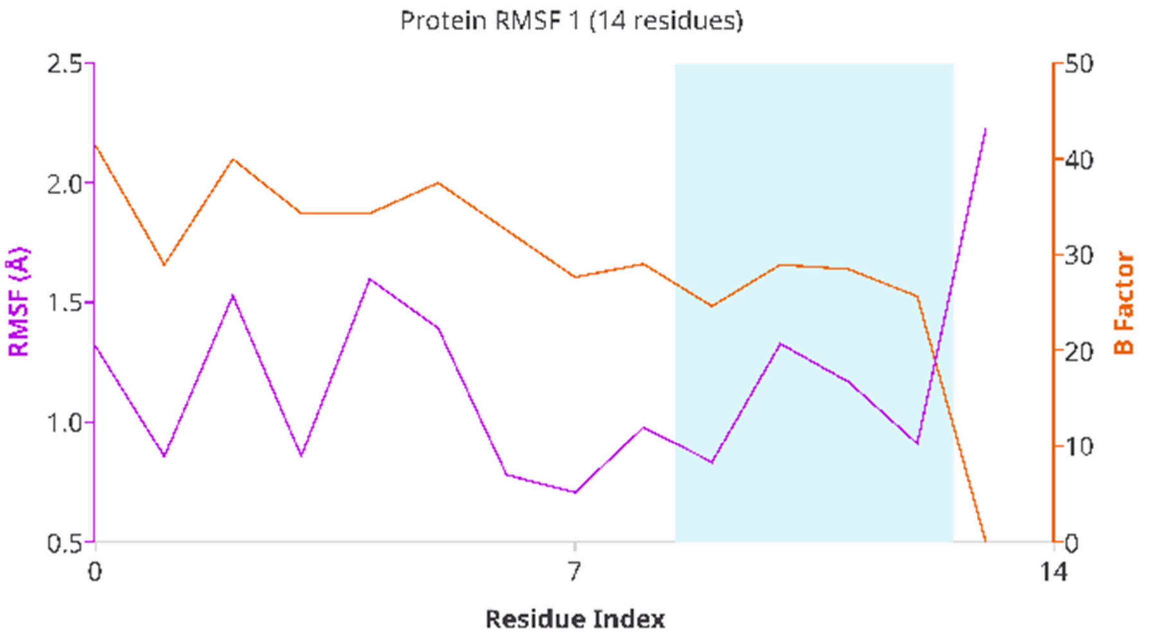

Radius of Gyration

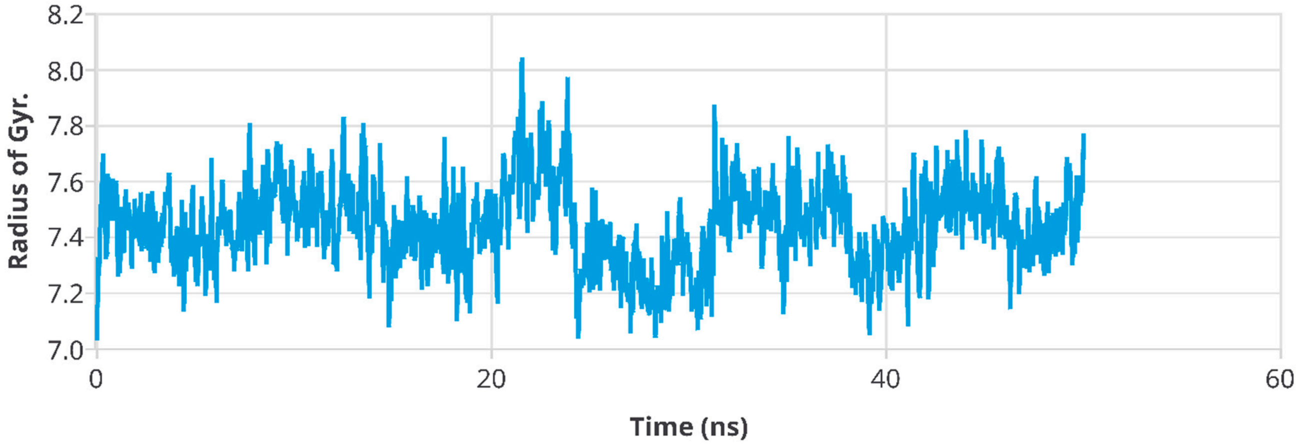

Molecular Surface Area

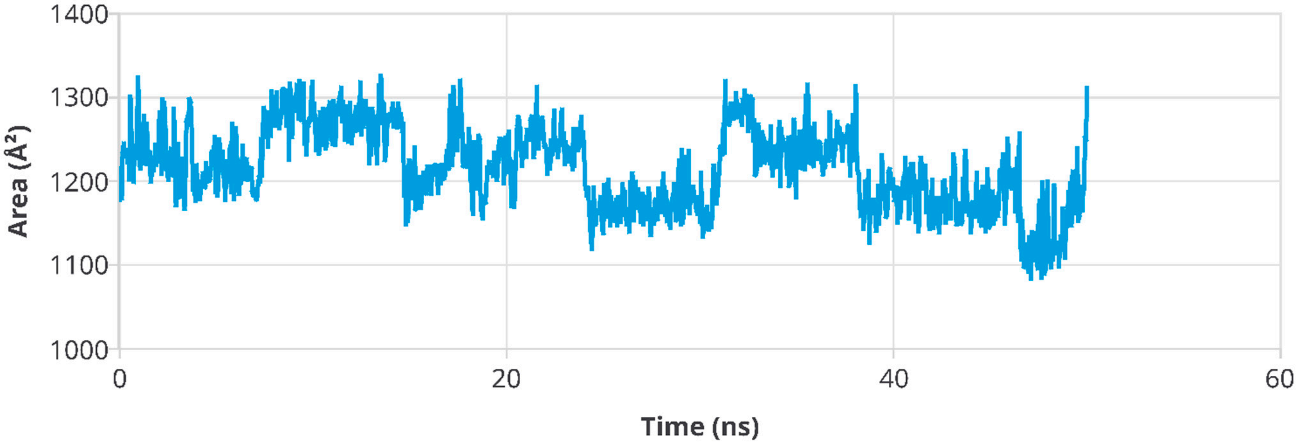

Polar Surface Area

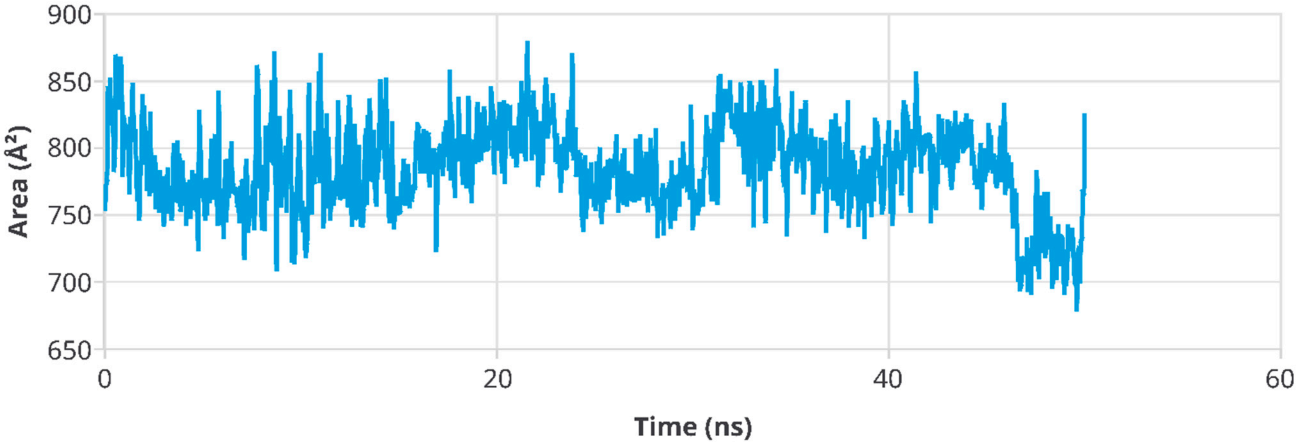

Radial Distribution Function (RDF)

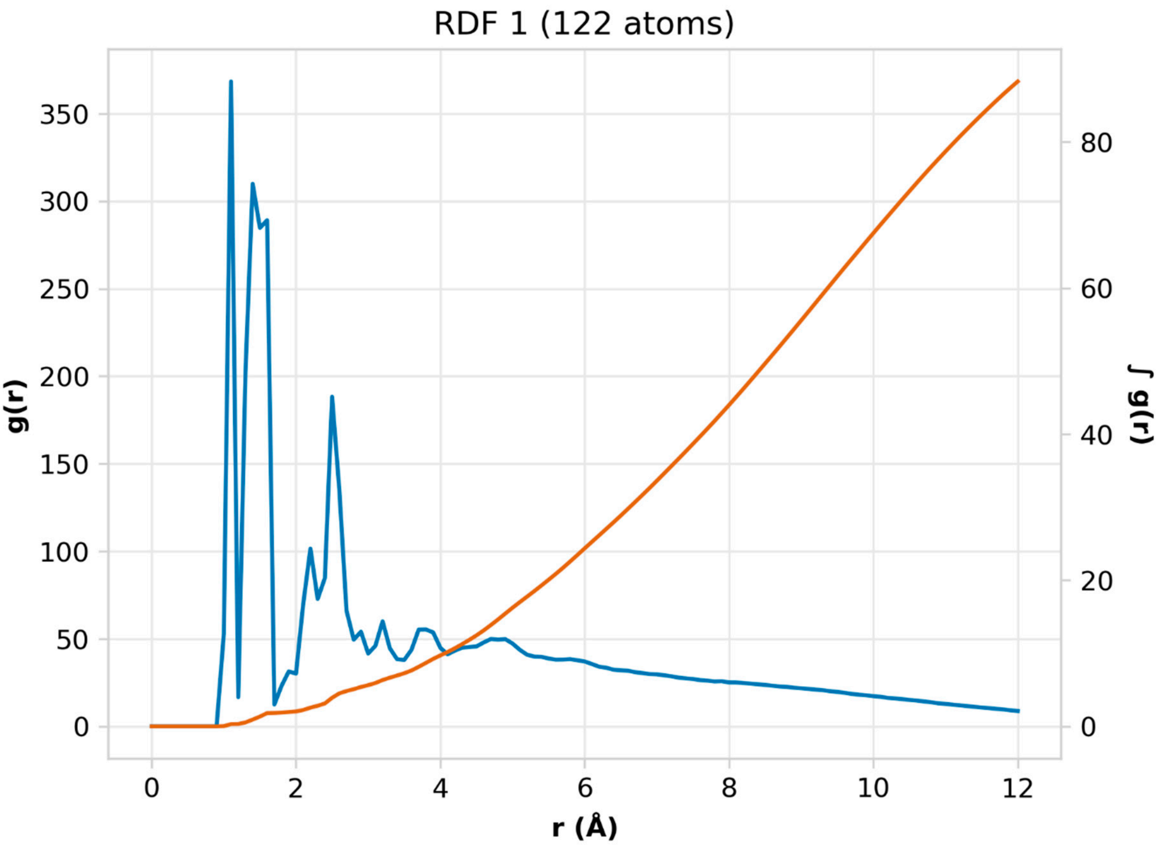

Solvent accessible surface area

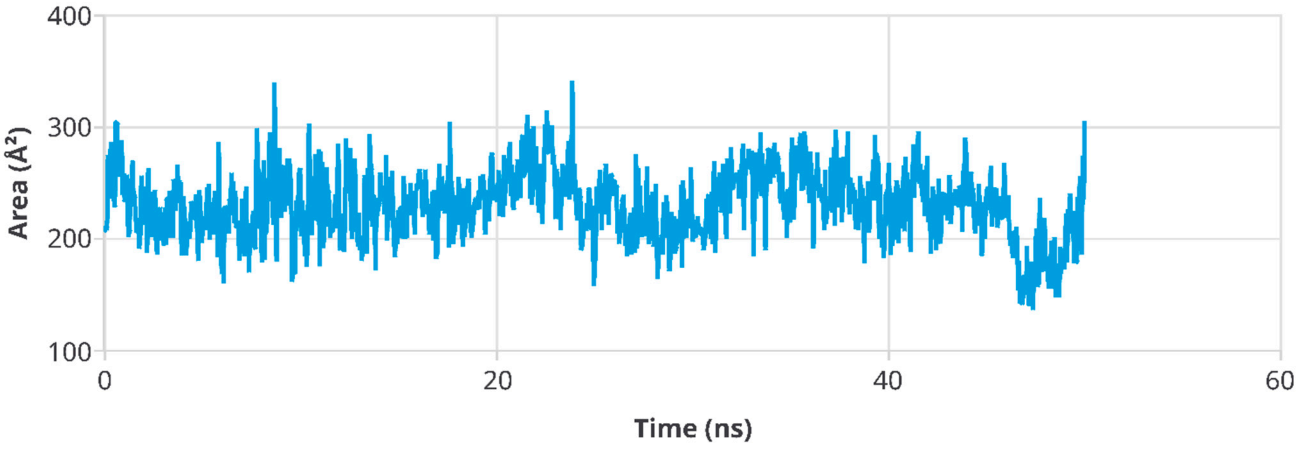
400
300
200
100
Area (Å²)
0
20
40
60
Time (ns)

Supplementary S2

Molecular dynamics between caryophyllene and target 7NIO protein of SARS-CoV-2.

Results

Interaction Counts

NOT FOUND

RMSD

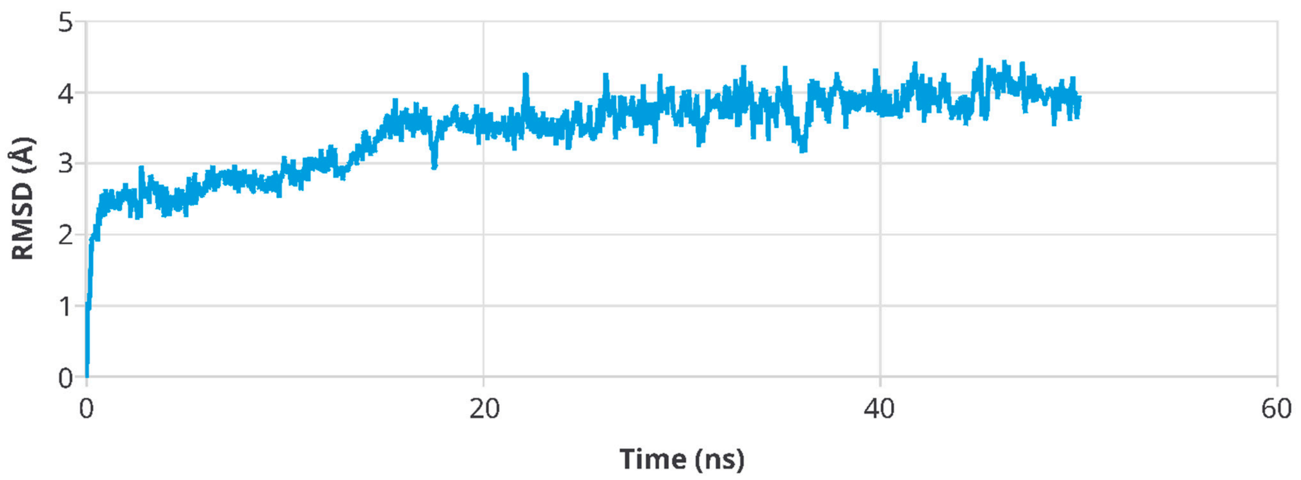

RMSF

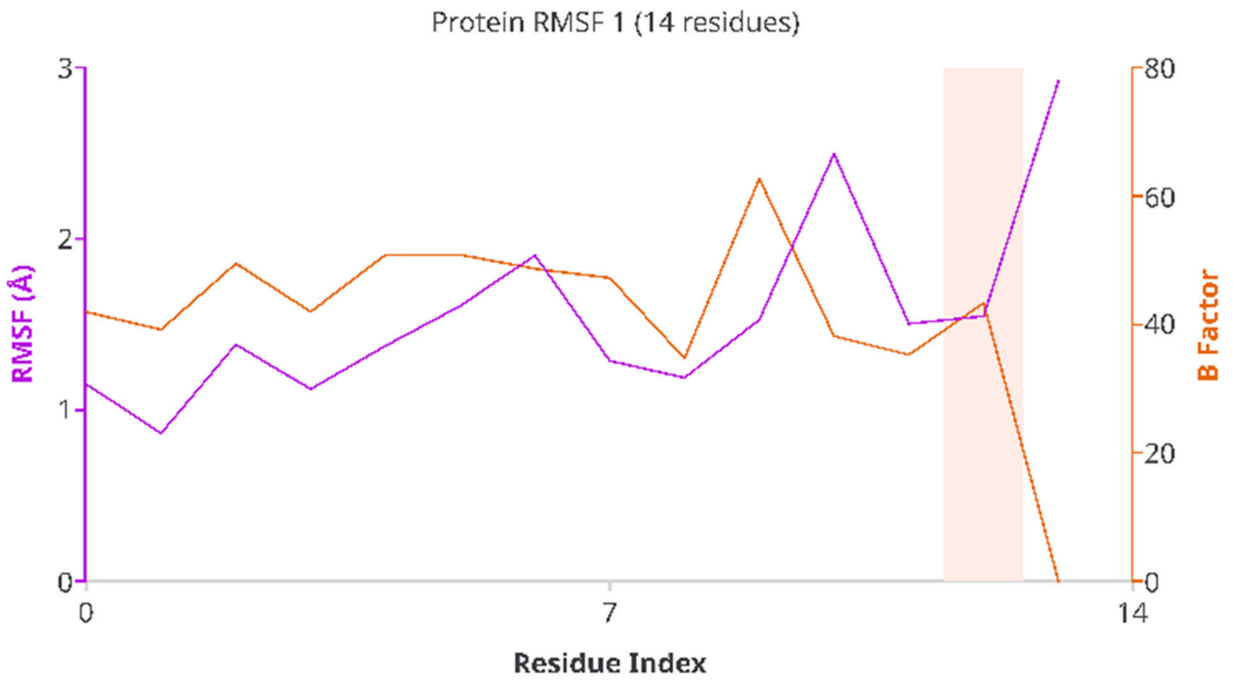

Radius of Gyration

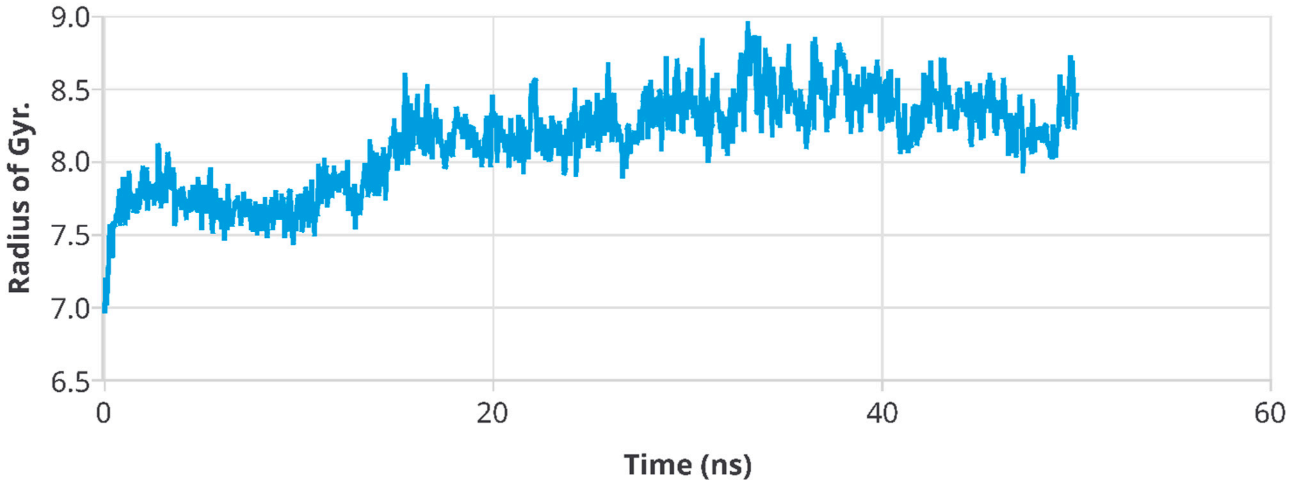

Molecular Surface Area

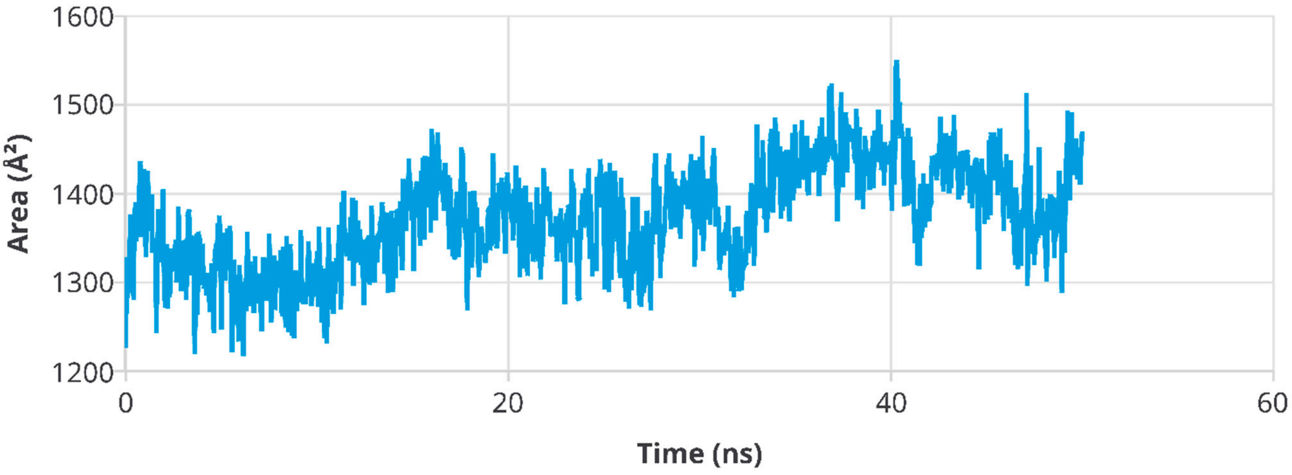

Polar Surface Area

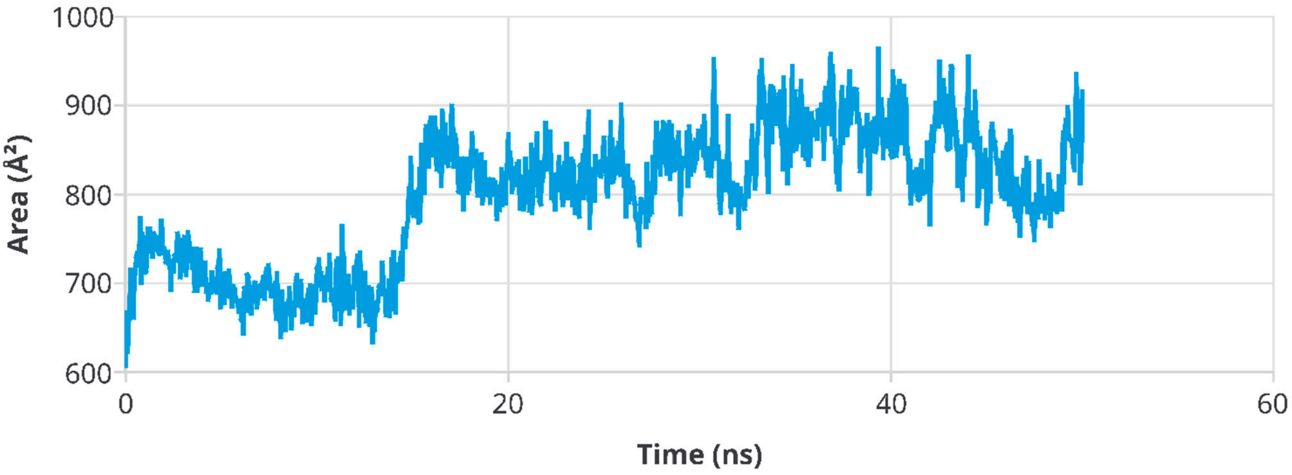

Radial Distribution Function (RDF)

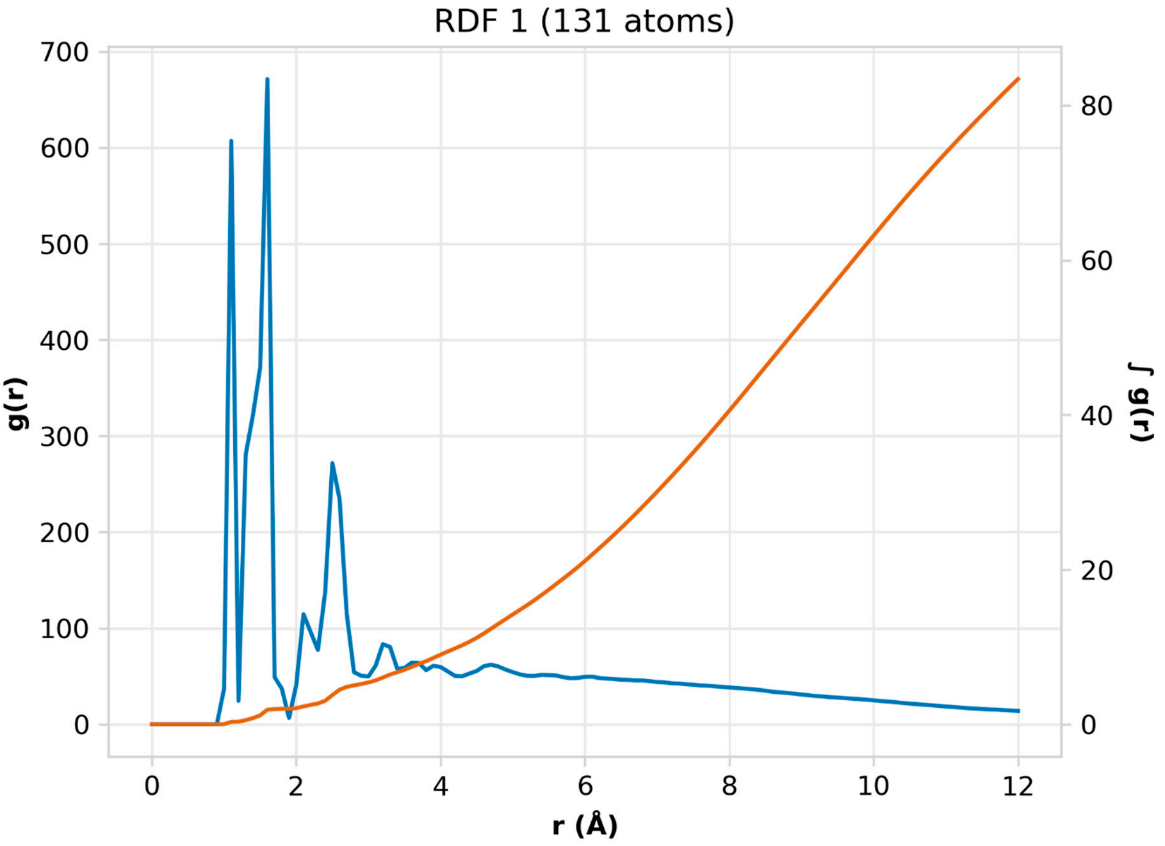

Solvent accessible surface area

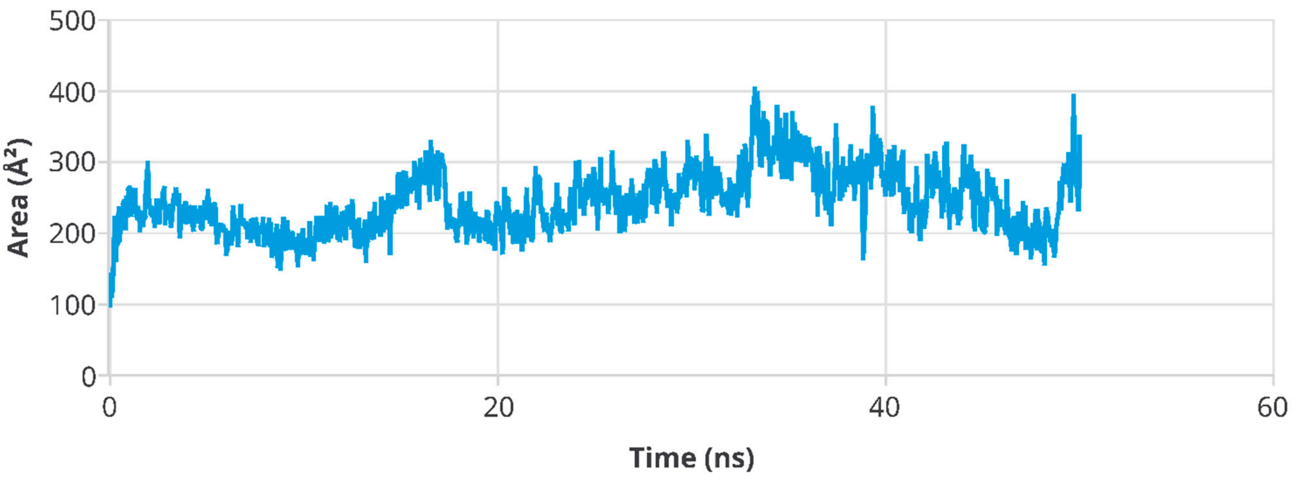

Molecular dynamics between caryophyllene and target 5X29 protein of SARS-CoV-2.

Results

Interaction Counts

NOT FOUND

RMSD

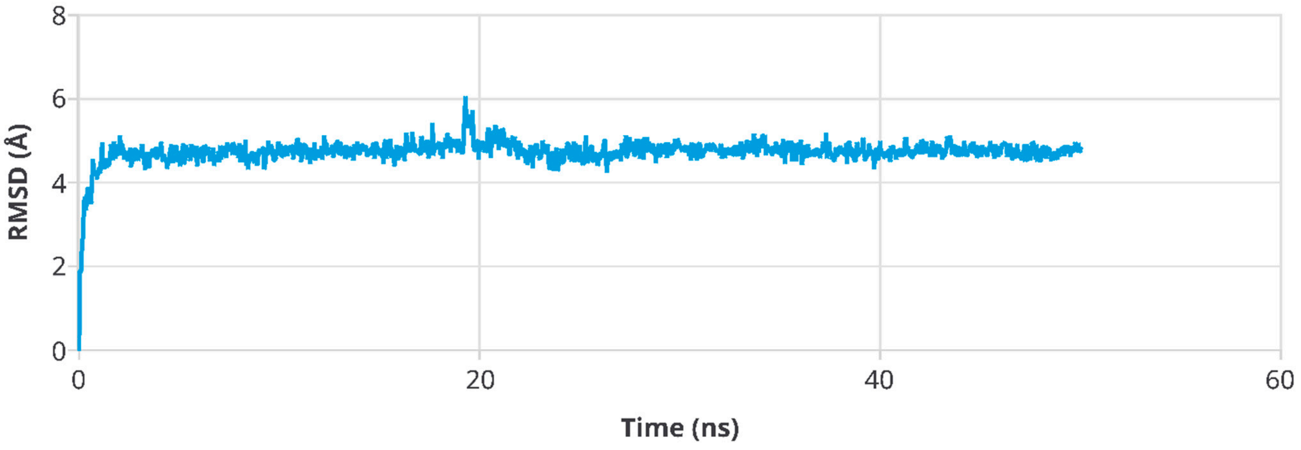

RMSF

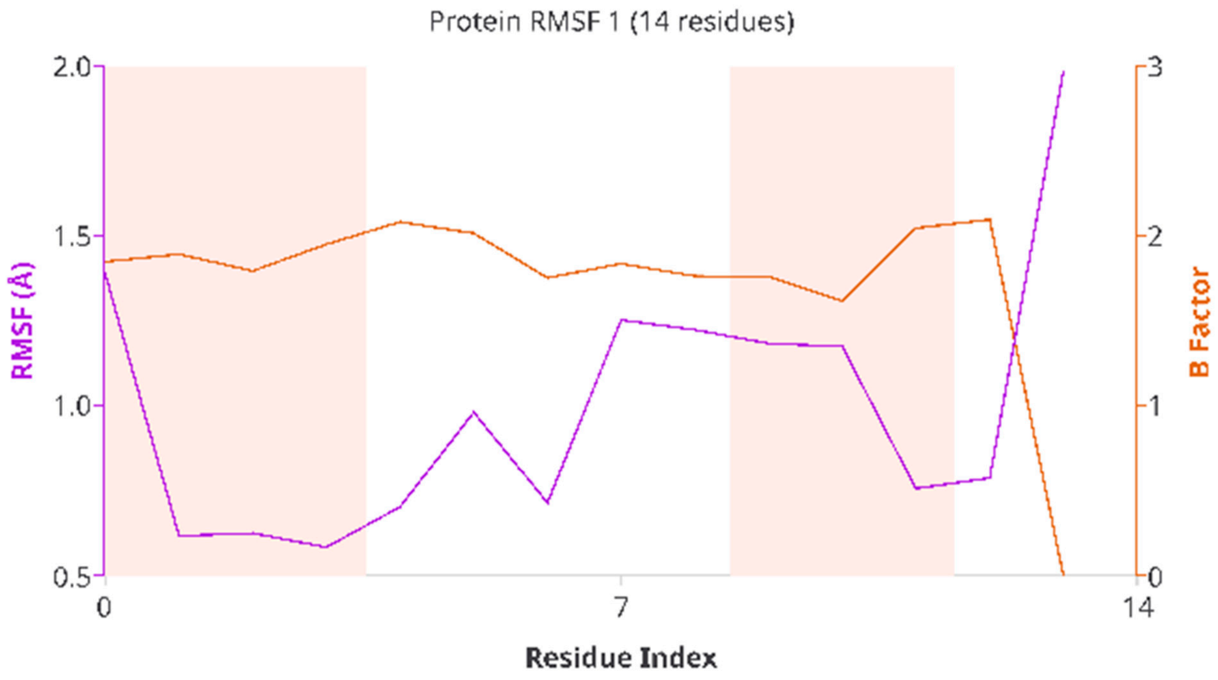

Radius of Gyration

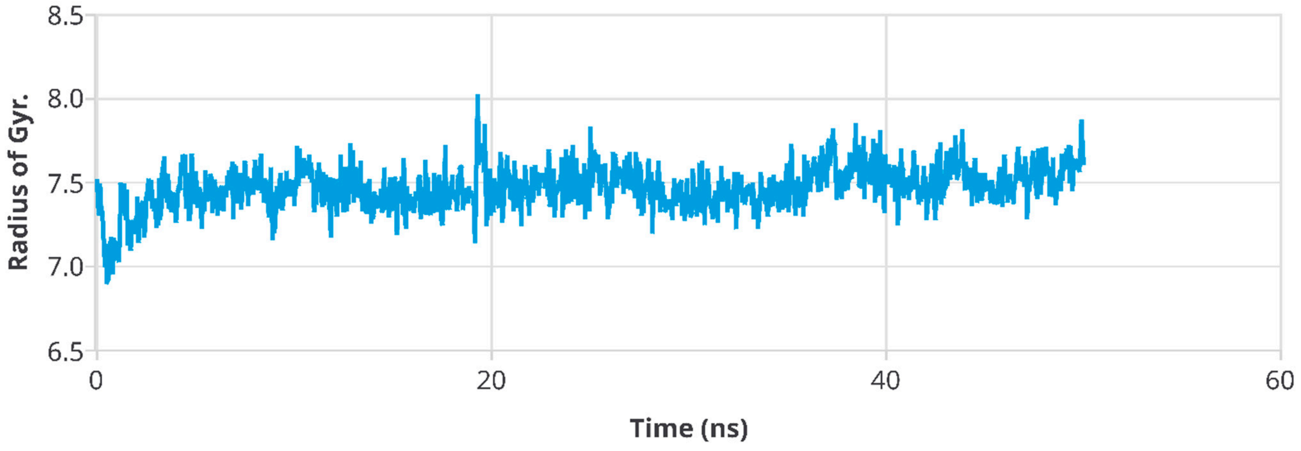

Molecular Surface Area

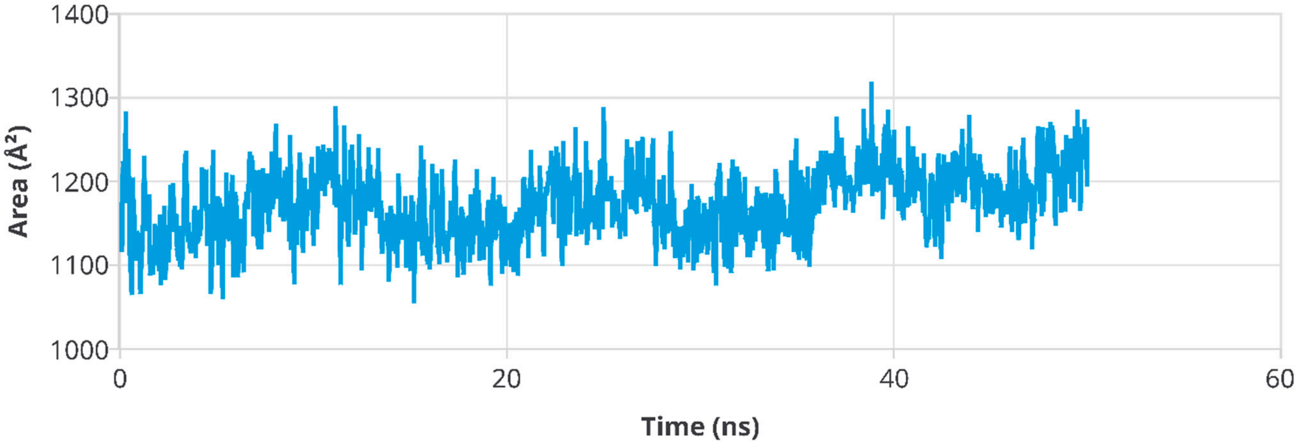

Polar Surface Area

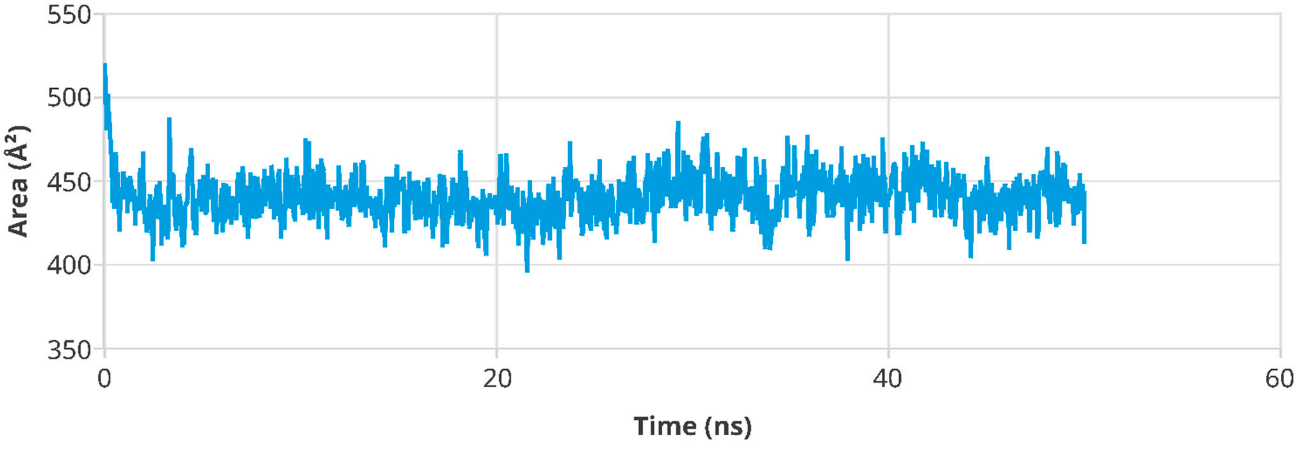

Radial Distribution Function (RDF)

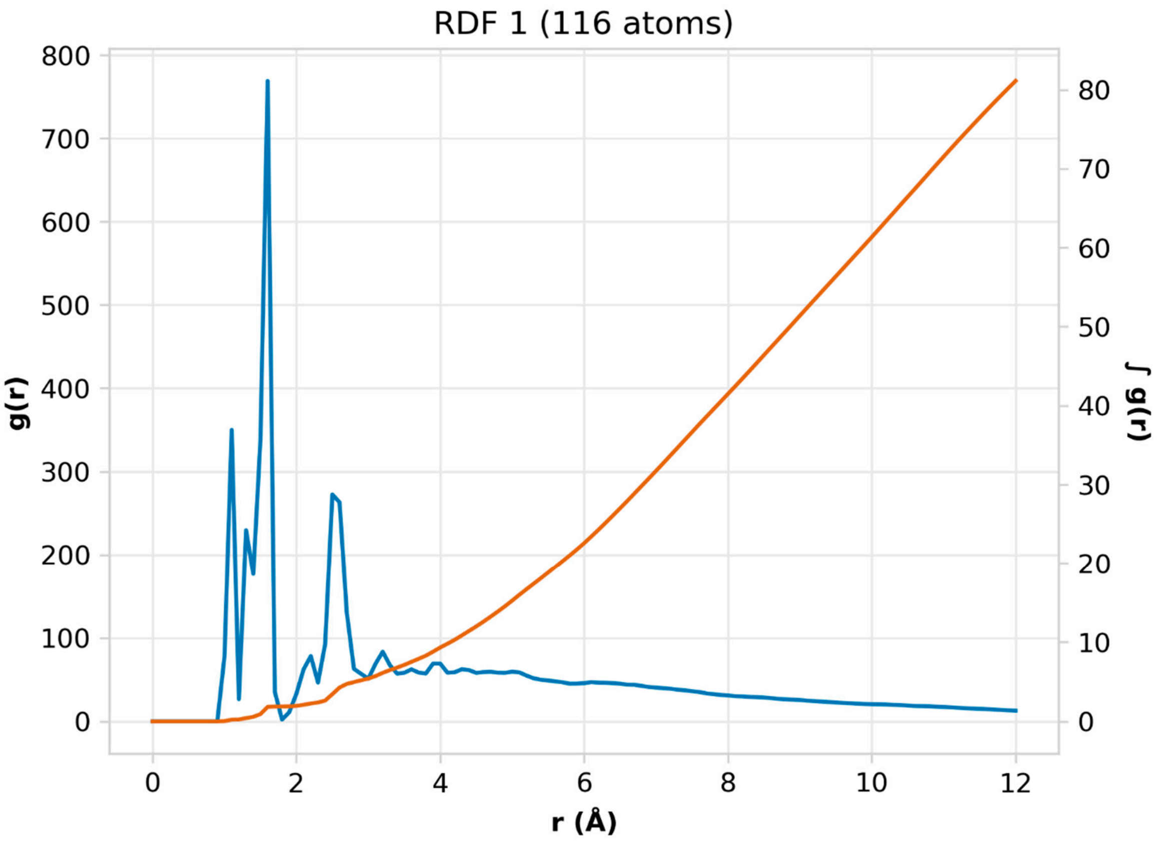

Solvent accessible surface area

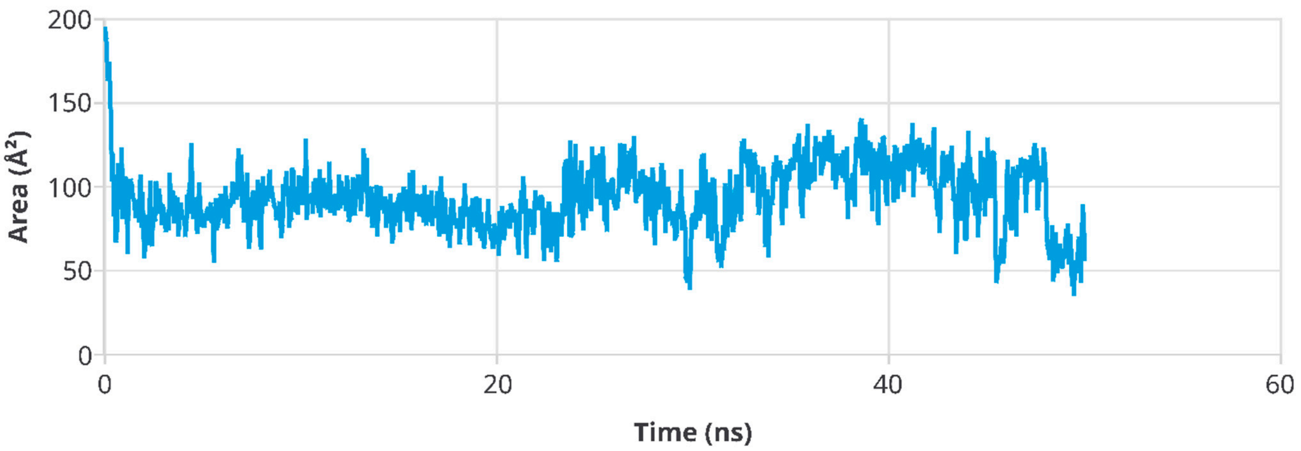

Molecular dynamics between caryophyllene and target 7B3O protein of SARS-CoV-2.

Results

Interaction Counts

NOT FOUND

RMSD

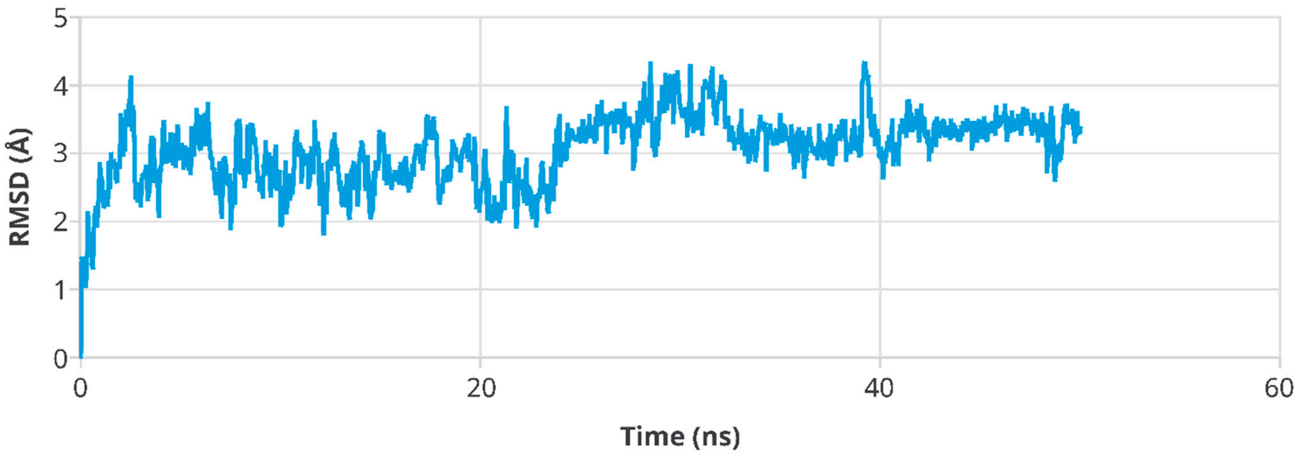

RMSF

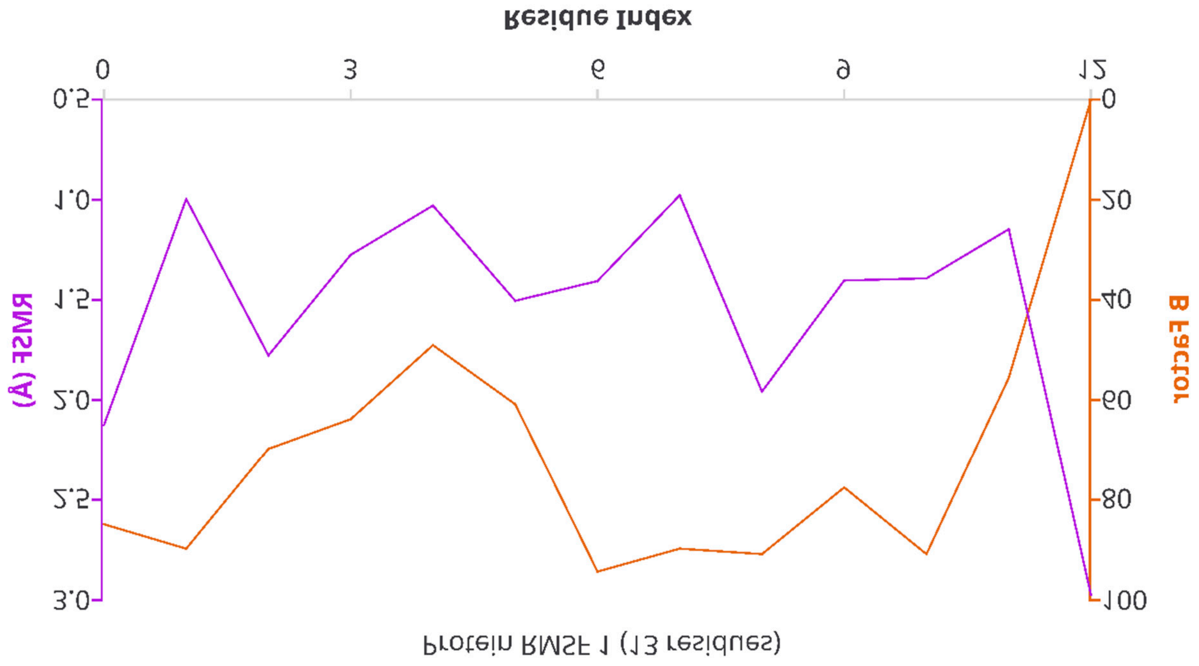

Radius of Gyration

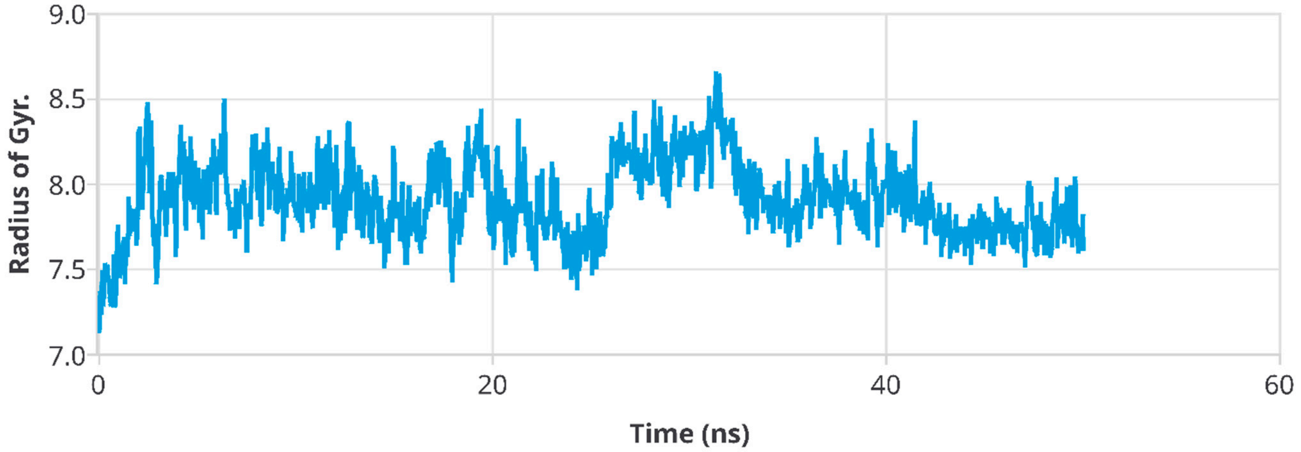

Molecular Surface Area

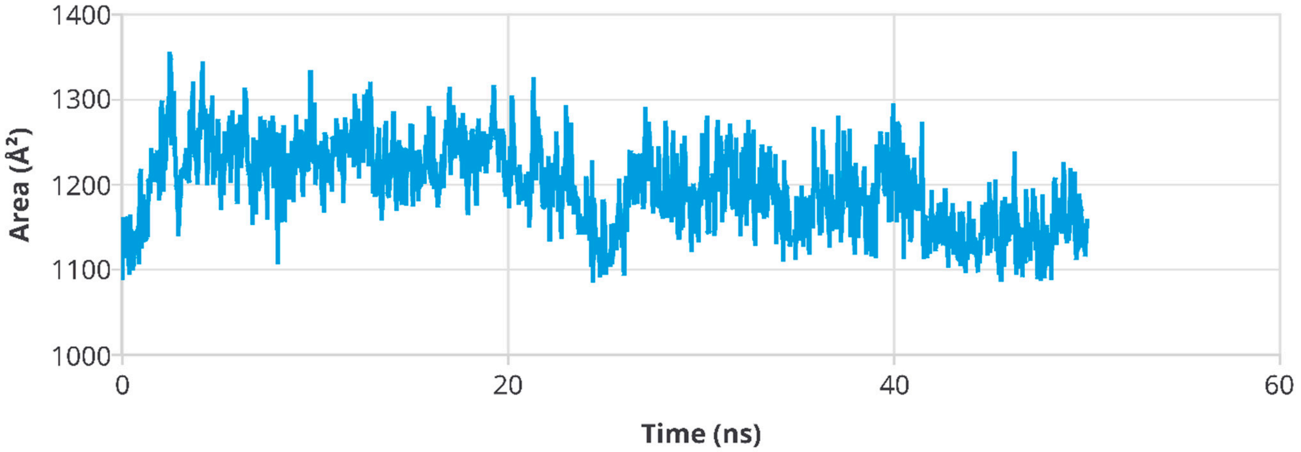

Polar Surface Area

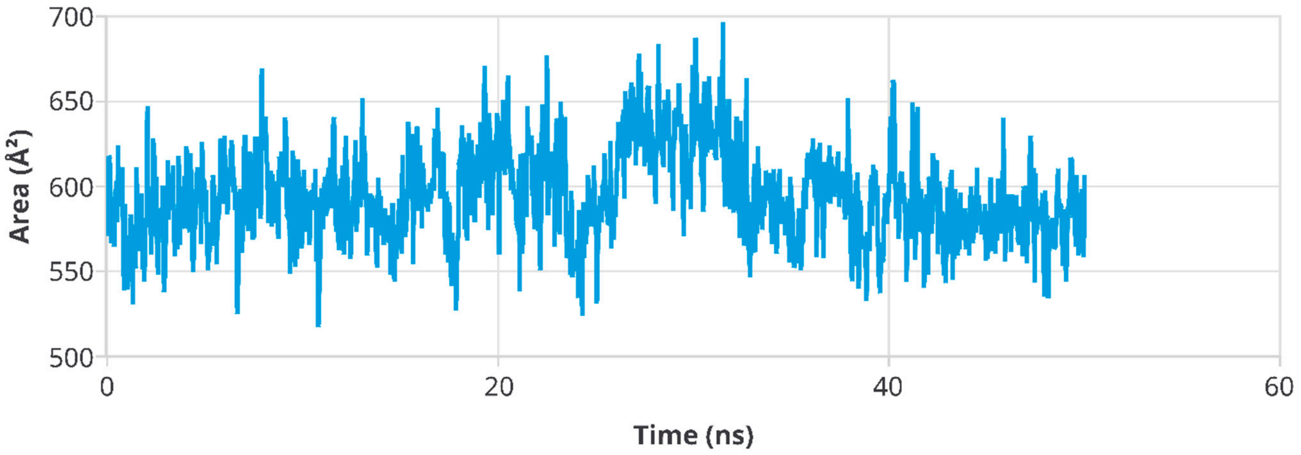

Radial Distribution Function (RDF)

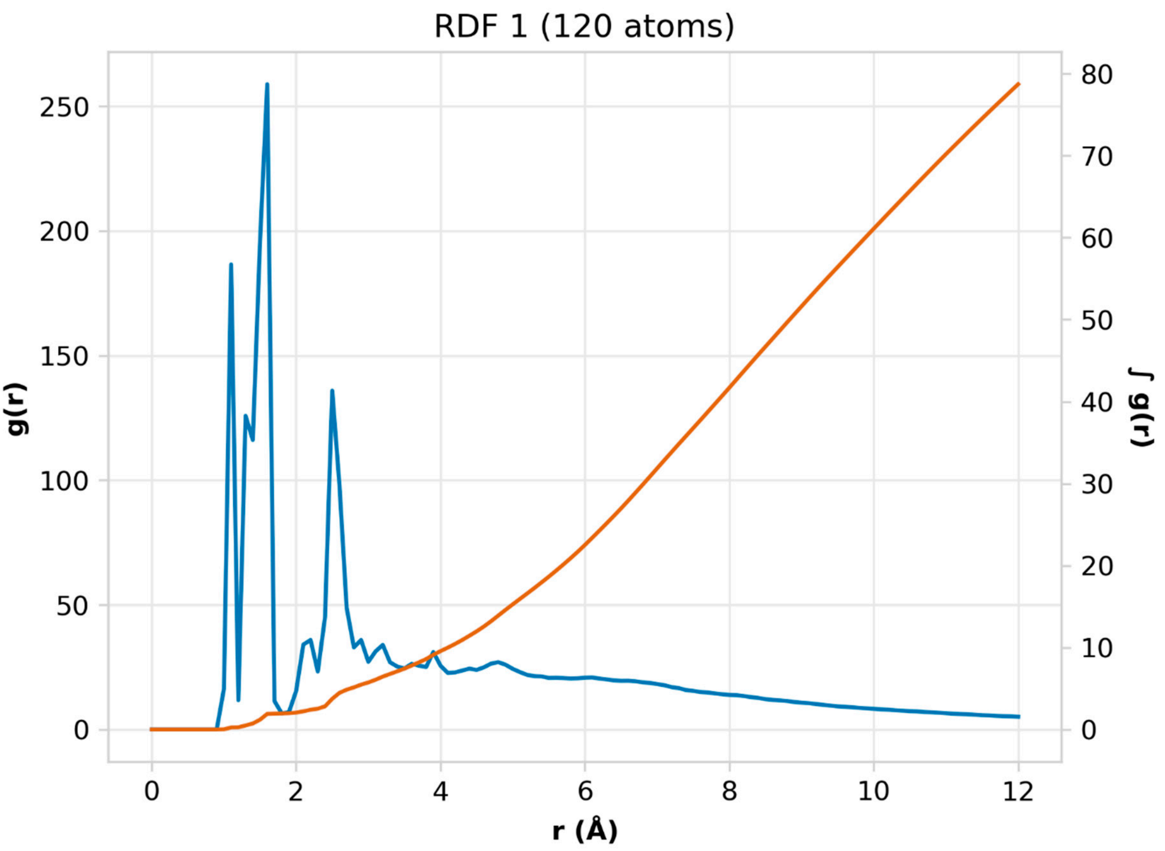

Solvent accessible surface area

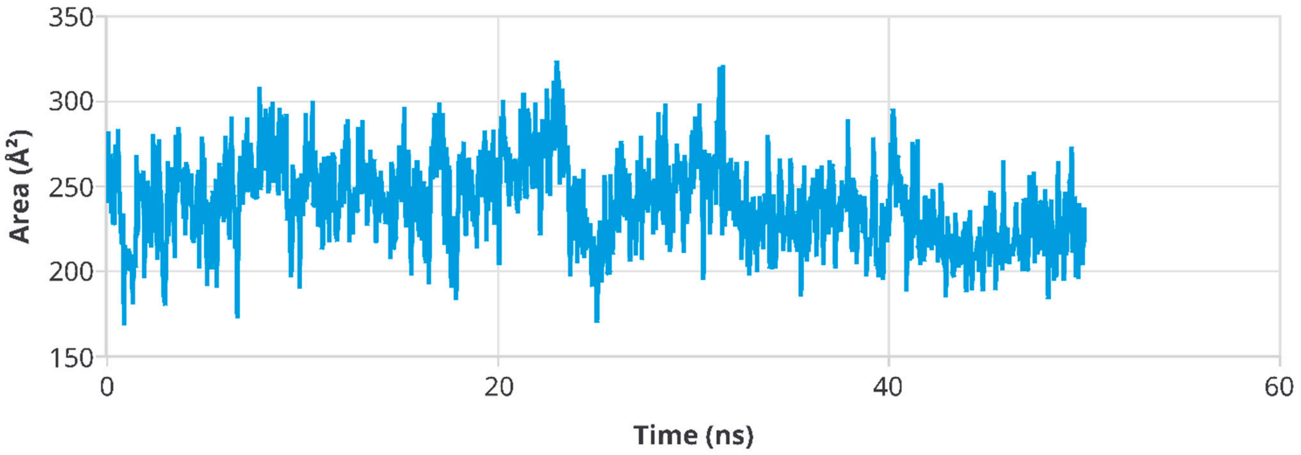

Molecular dynamics between caryophyllene and target 2GTH protein of SARS-CoV-2.

Results

Interaction Counts

NOT FOUND

RMSD

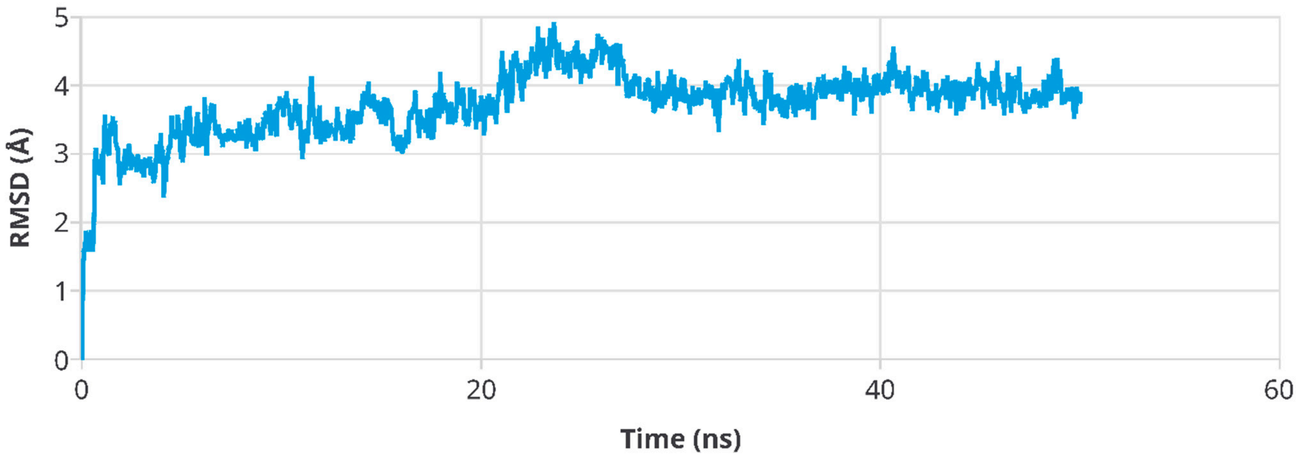

RMSF

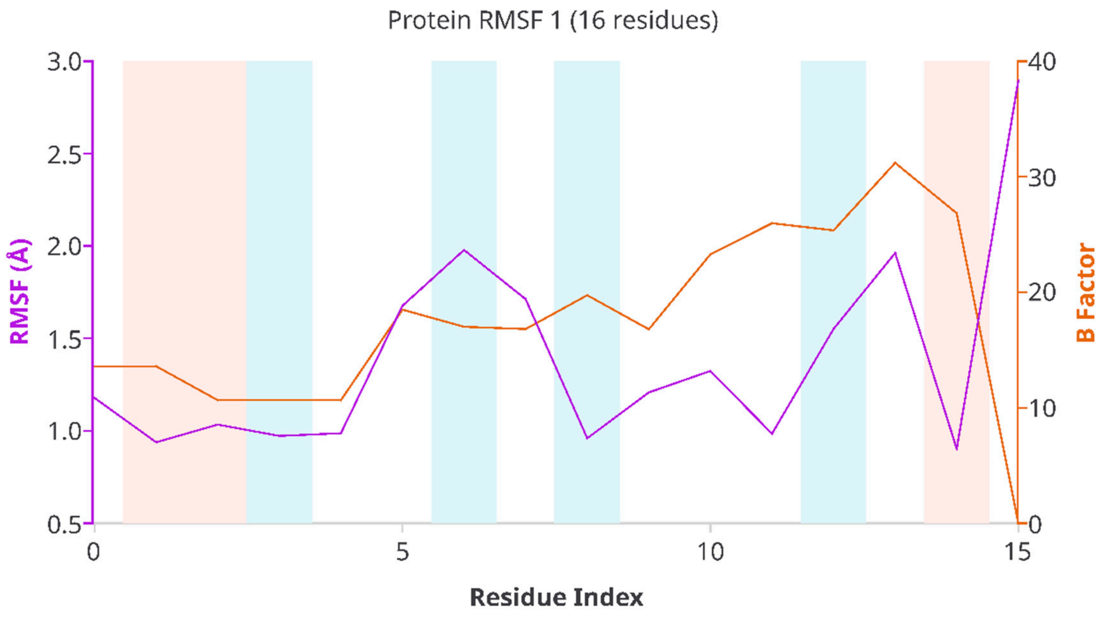

Radius of Gyration

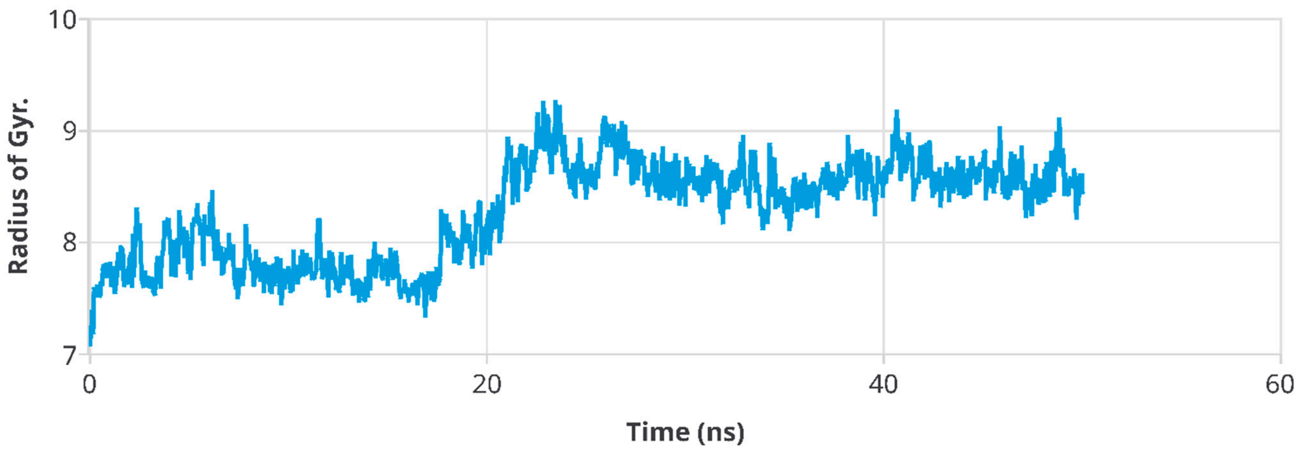

Molecular Surface Area

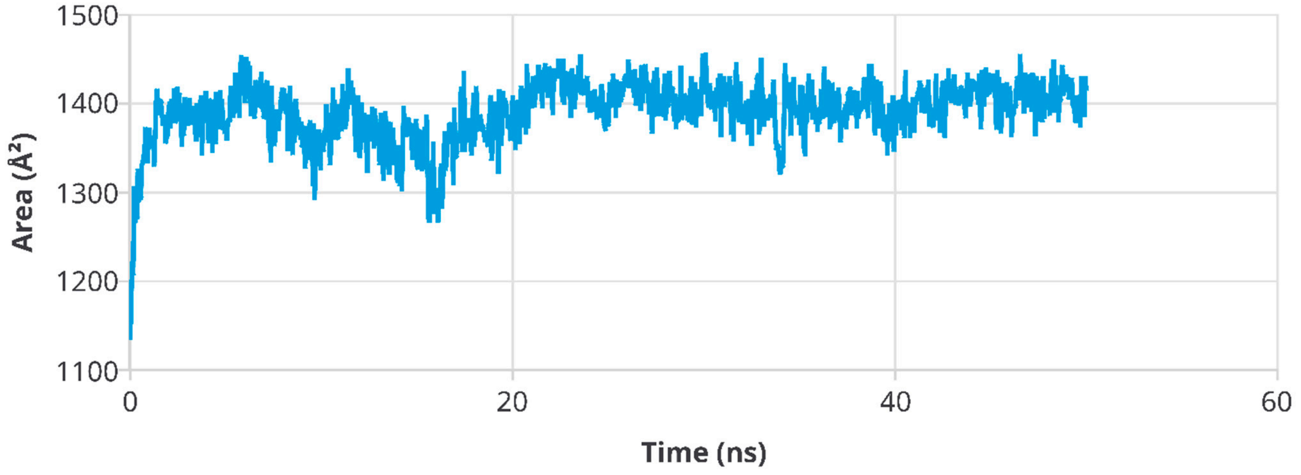

Polar Surface Area

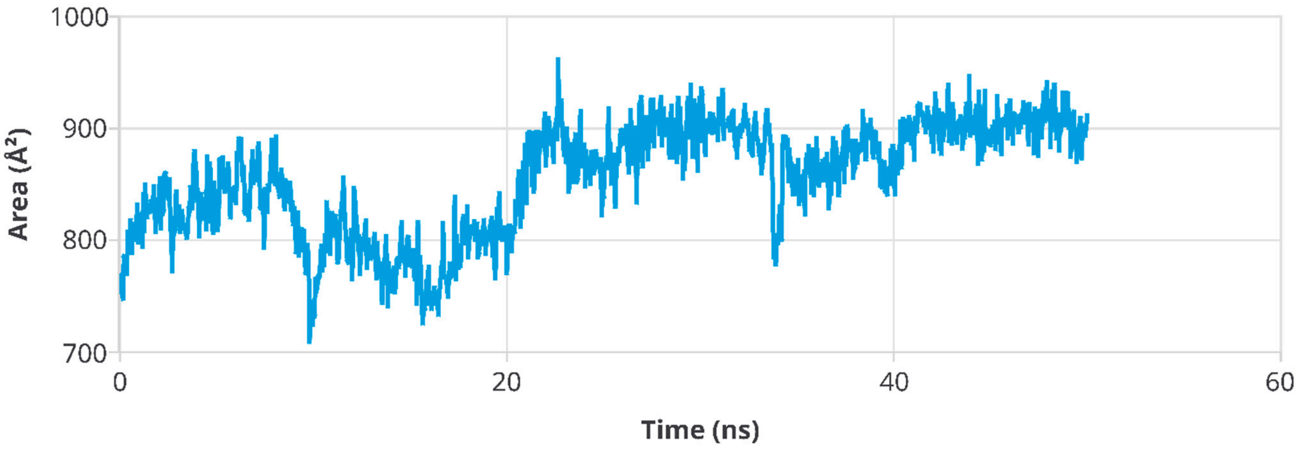

Radial Distribution Function (RDF)

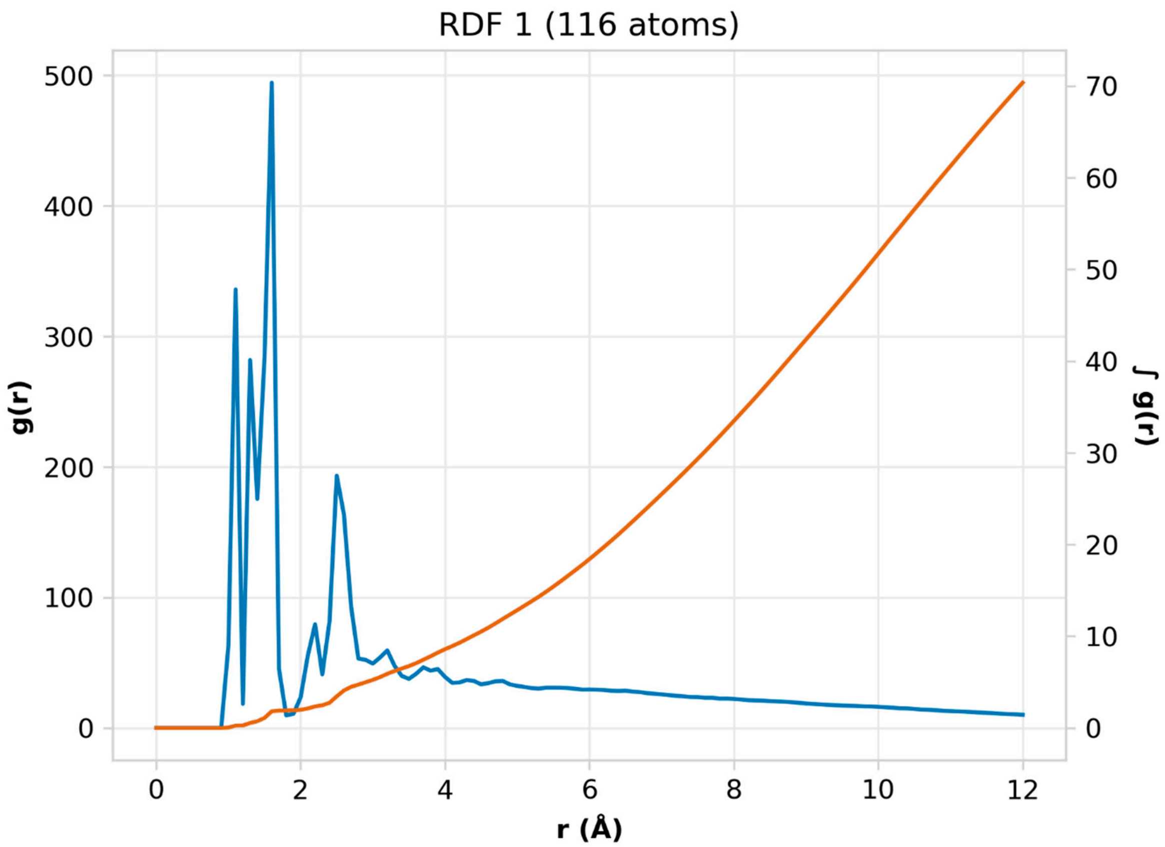

Solvent accessible surface area

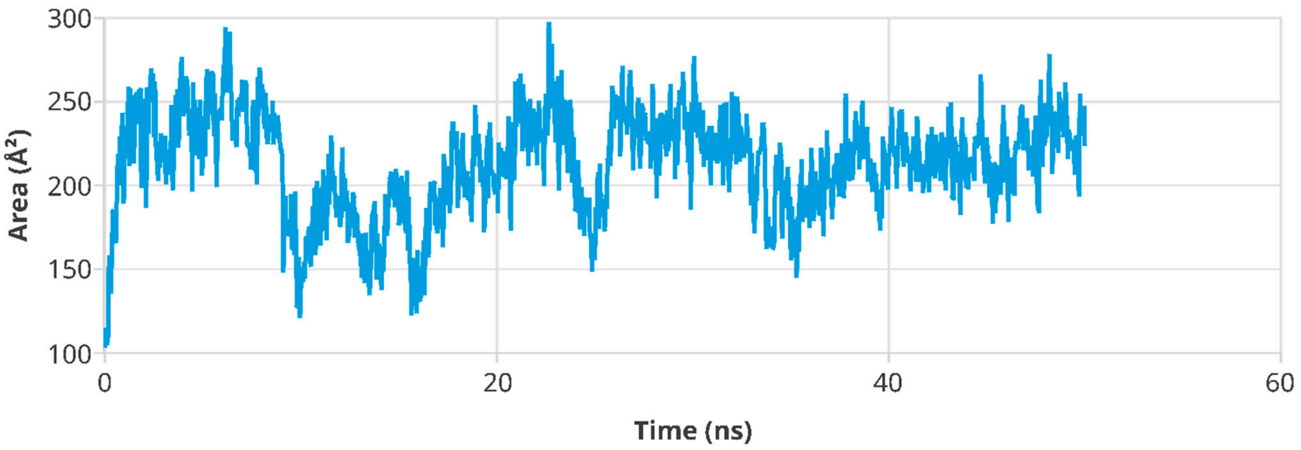

Molecular dynamics between caryophyllene and target 5YNB protein of SARS-CoV-2.

Results

Interaction Counts

NOT FOUND

RMSD

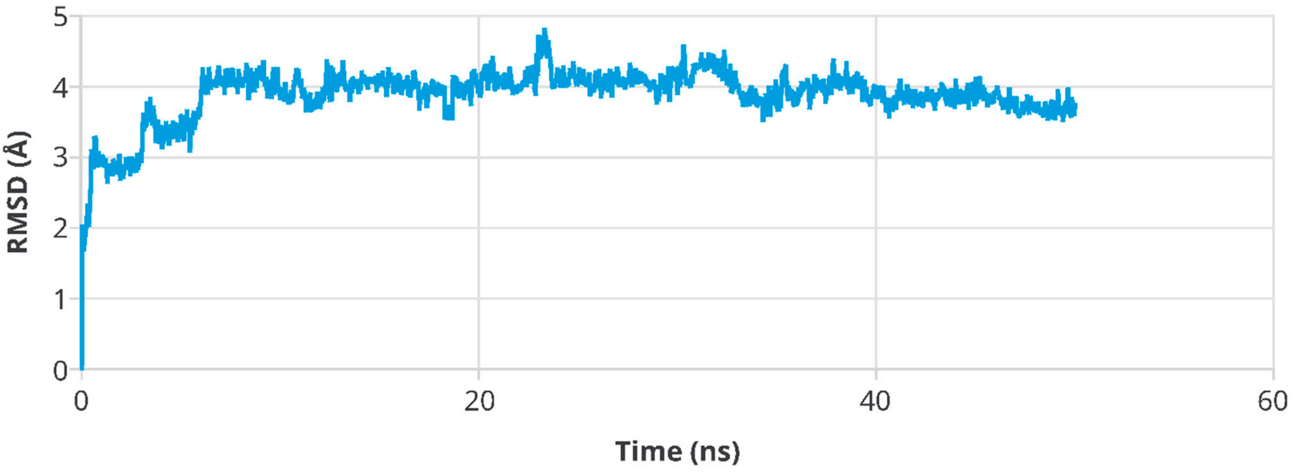

RMSF

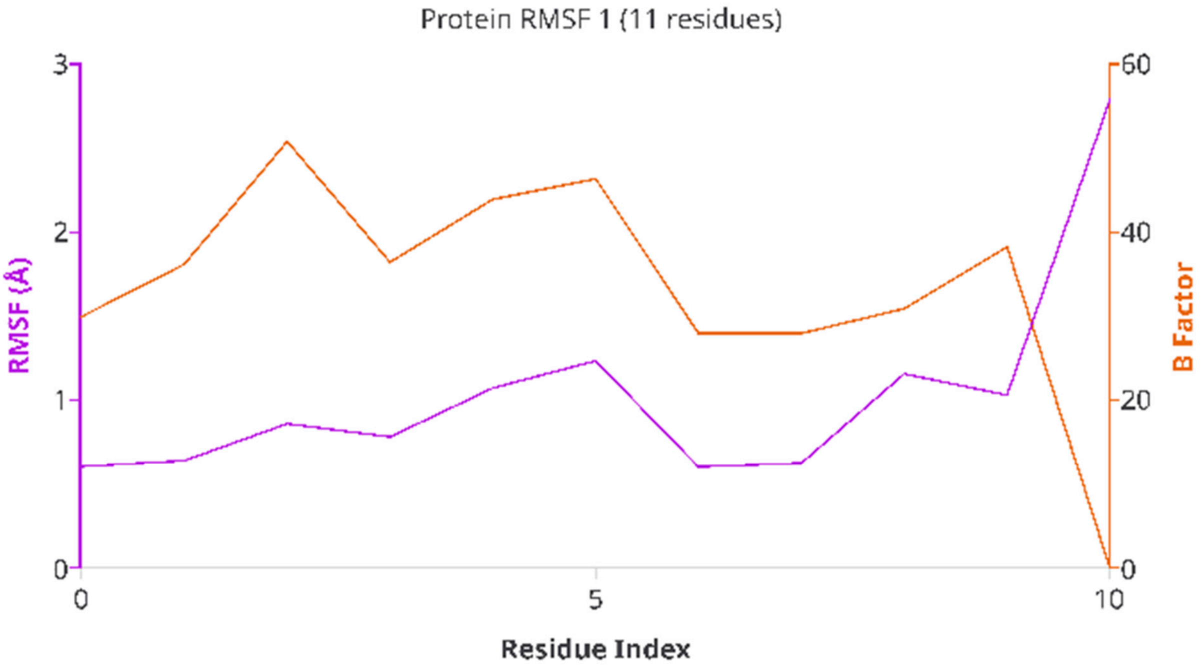

Radius of Gyration

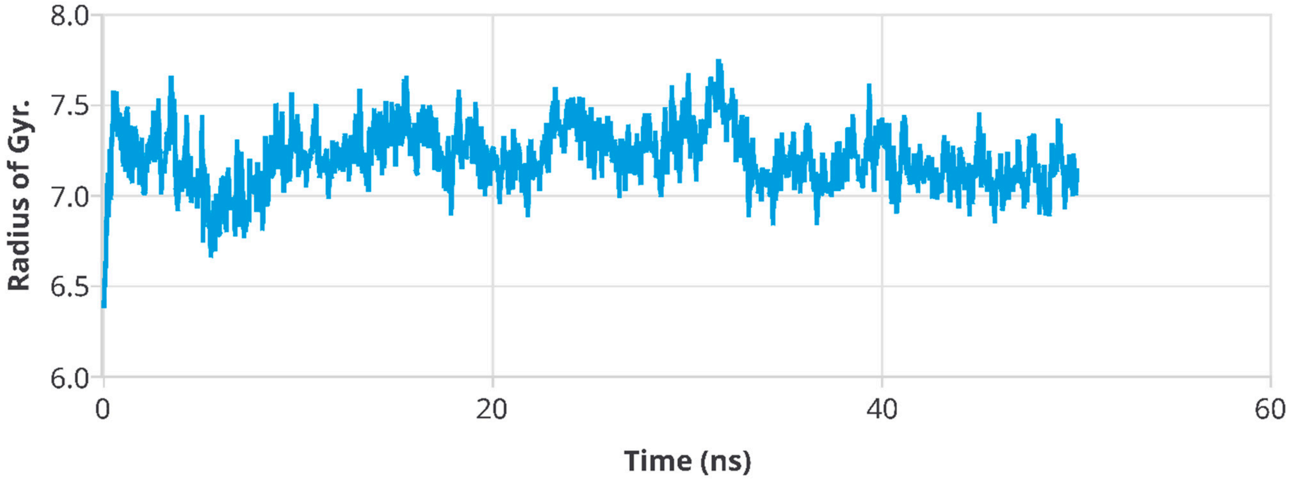

Molecular Surface Area

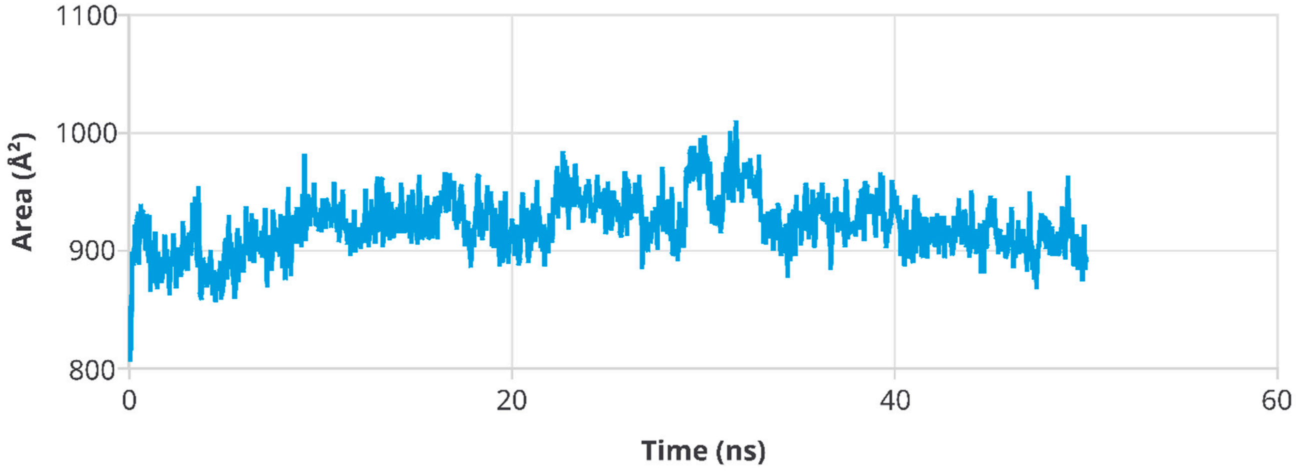

Polar Surface Area

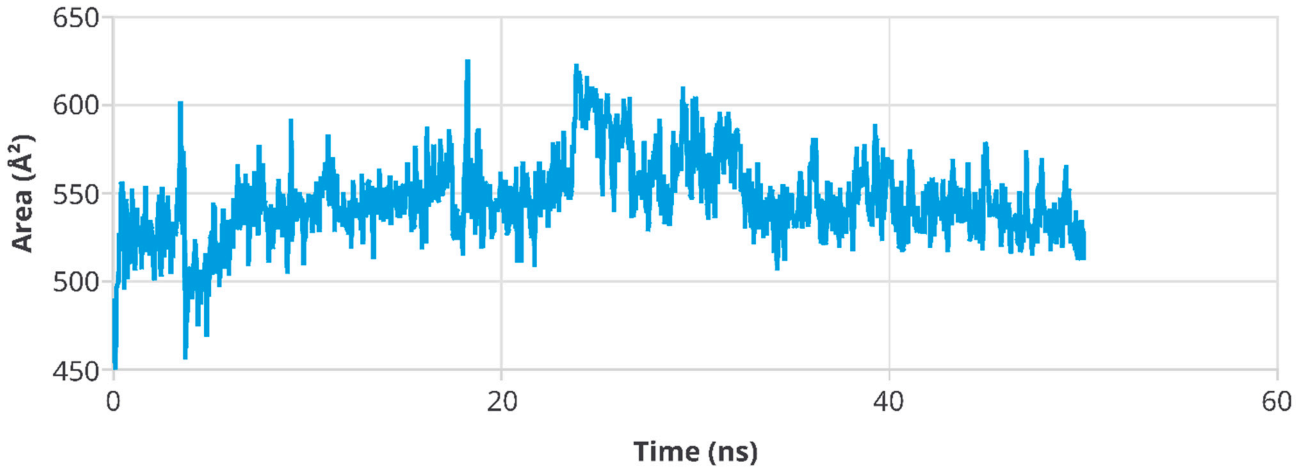

Radial Distribution Function (RDF)

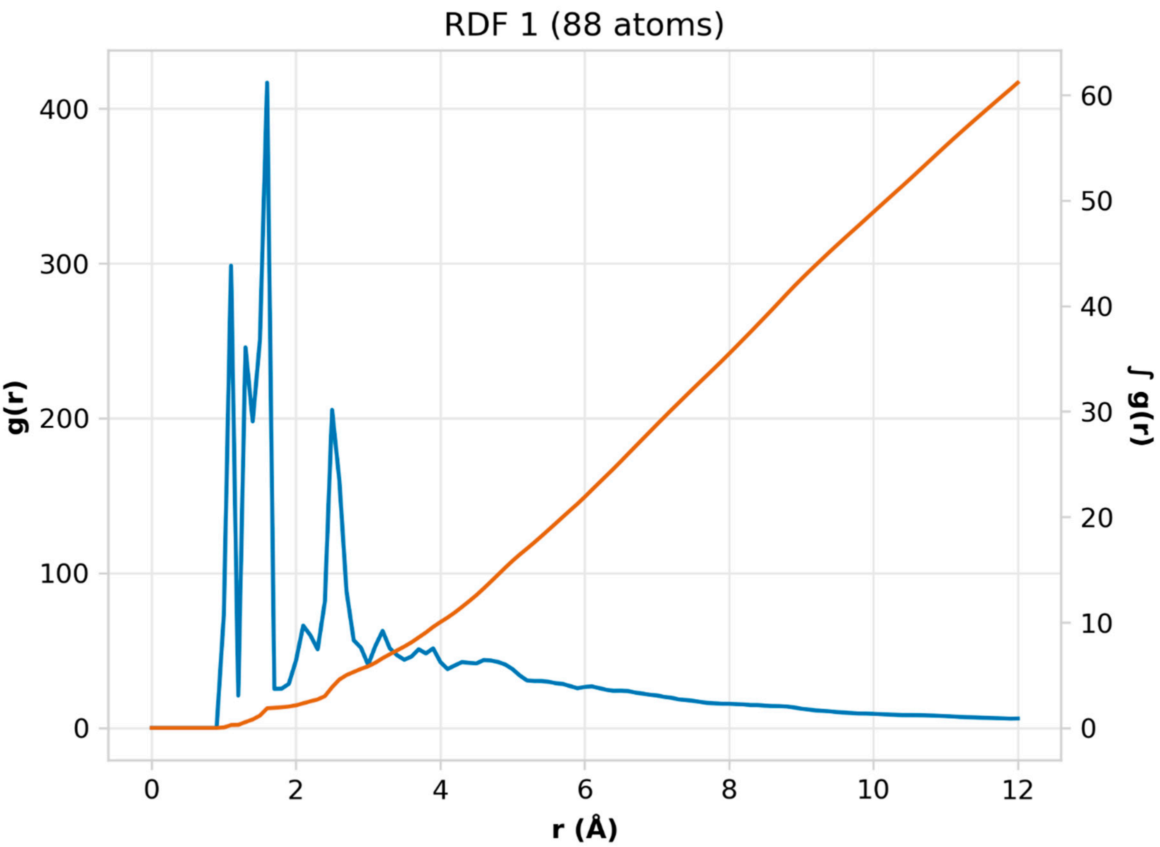

Solvent accessible surface area

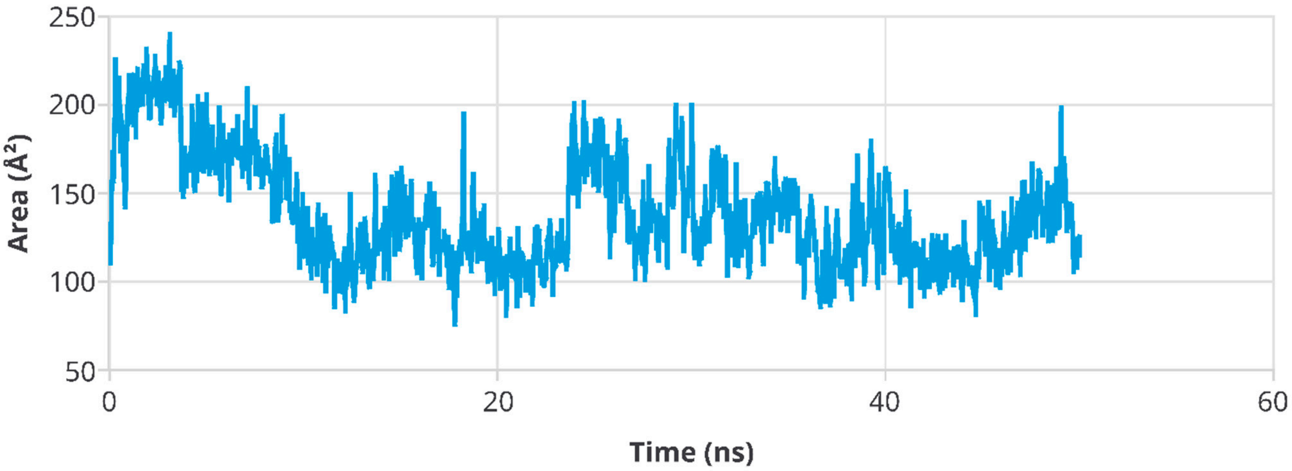

Molecular dynamics between caryophyllene and target 5CPO protein of SARS-CoV-2.

Results

Interaction Counts

NOT FOUND

RMSD

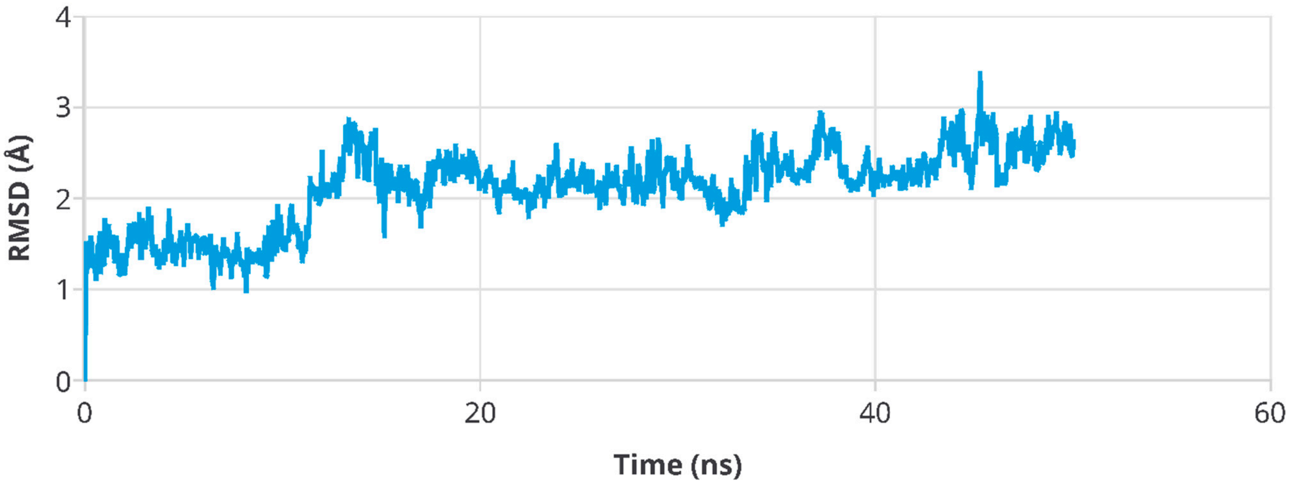

RMSF

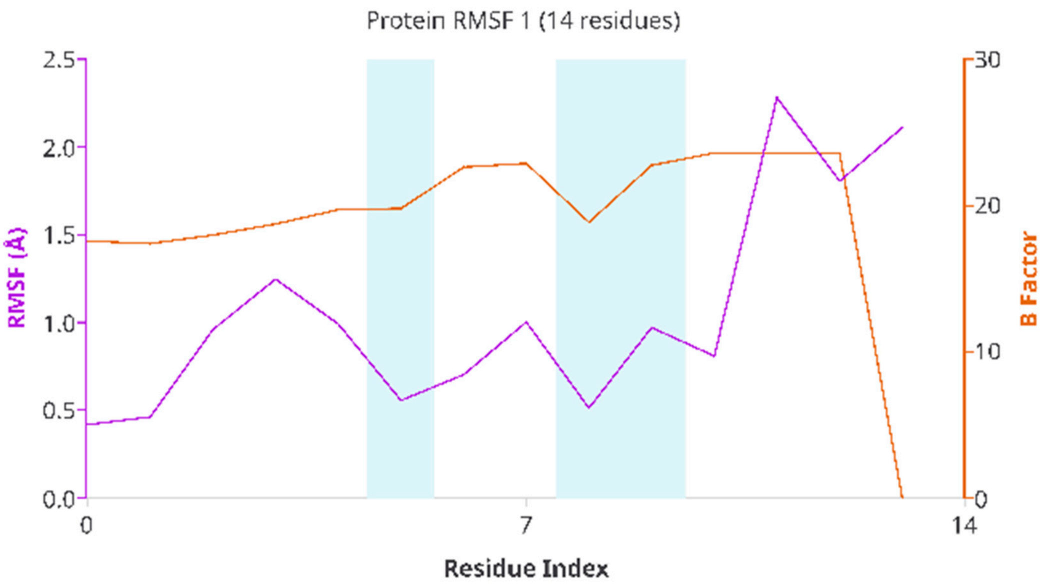

Radius of Gyration

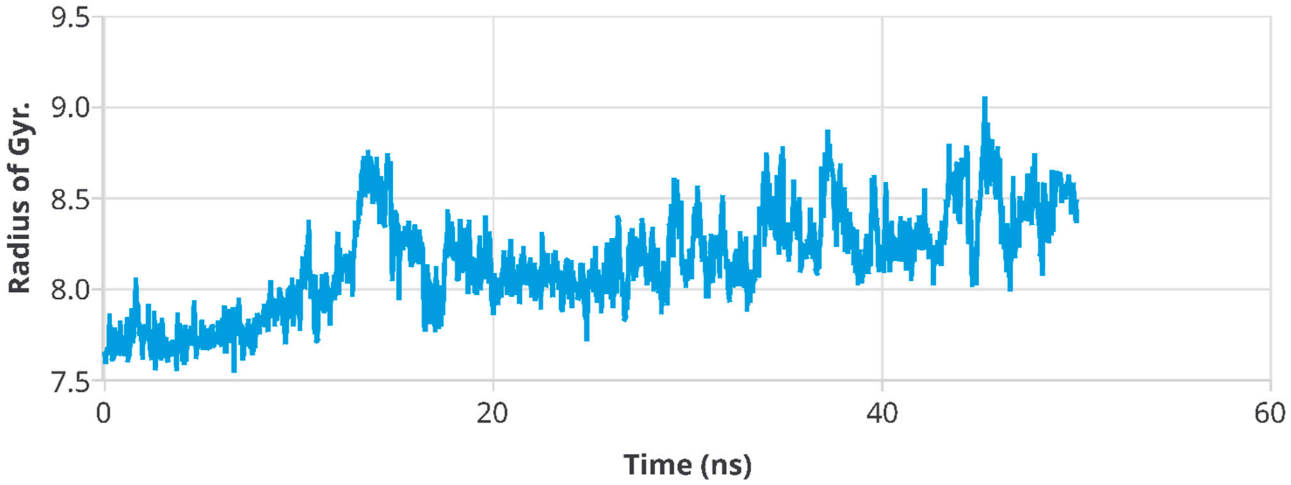

Molecular Surface Area

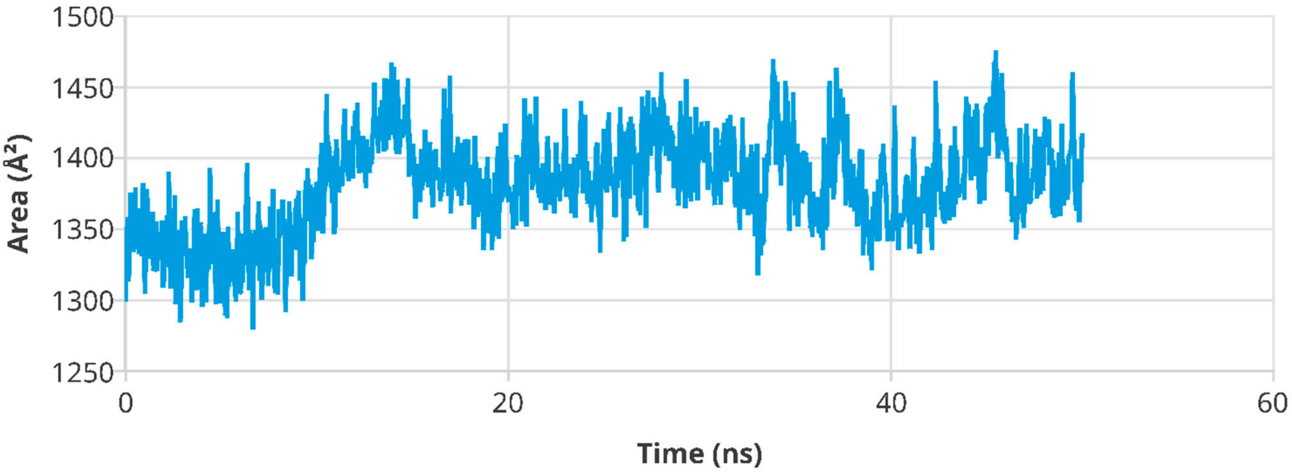

Polar Surface Area

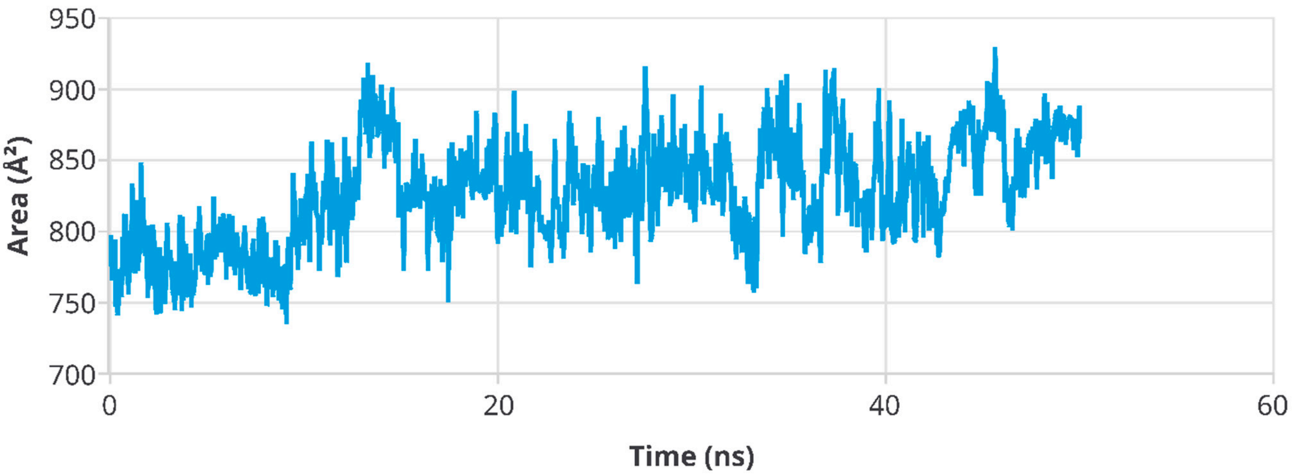

Radial Distribution Function (RDF)

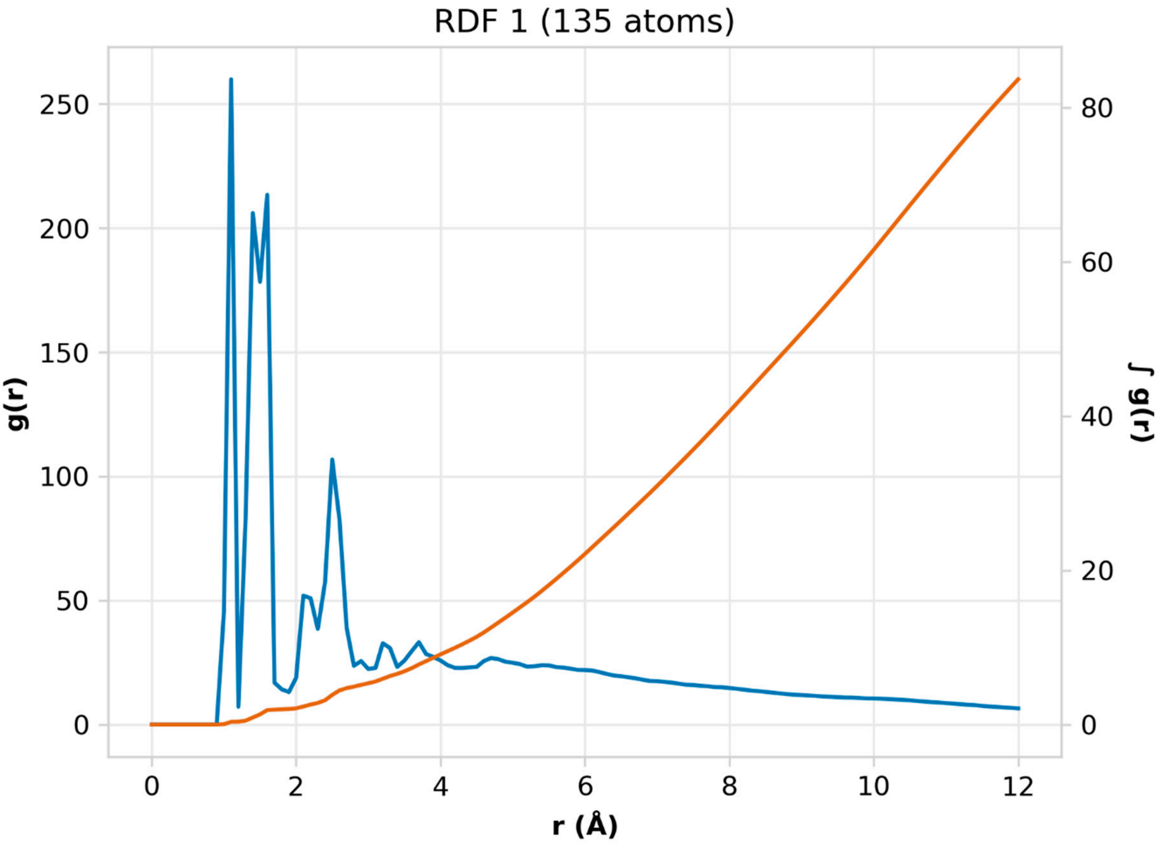

Solvent accessible surface area

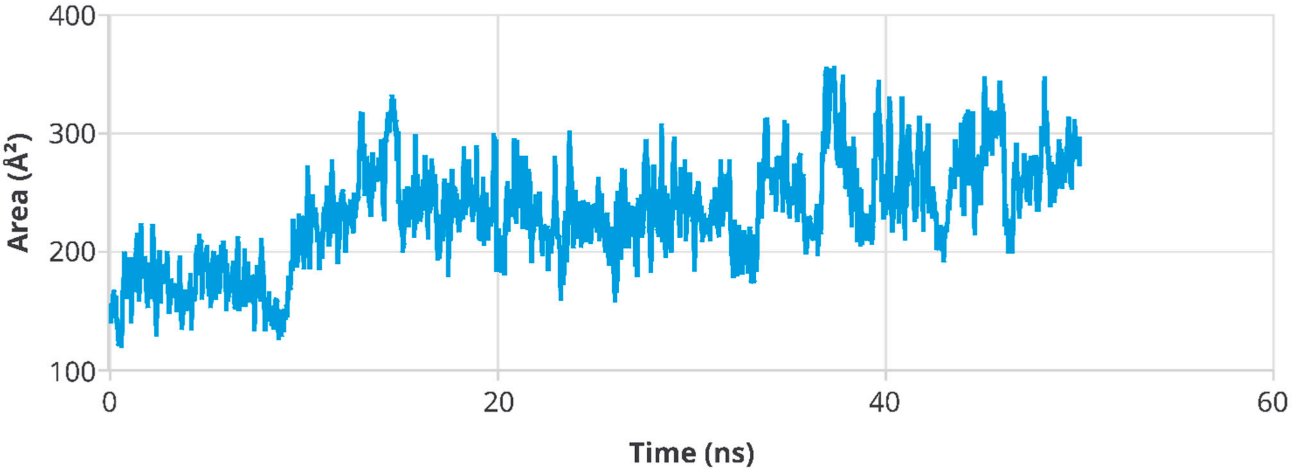

Molecular dynamics between caryophyllene and target 7Z4S protein of SARS-CoV-2.

Results

Interaction Counts

NOT FOUND

RMSD

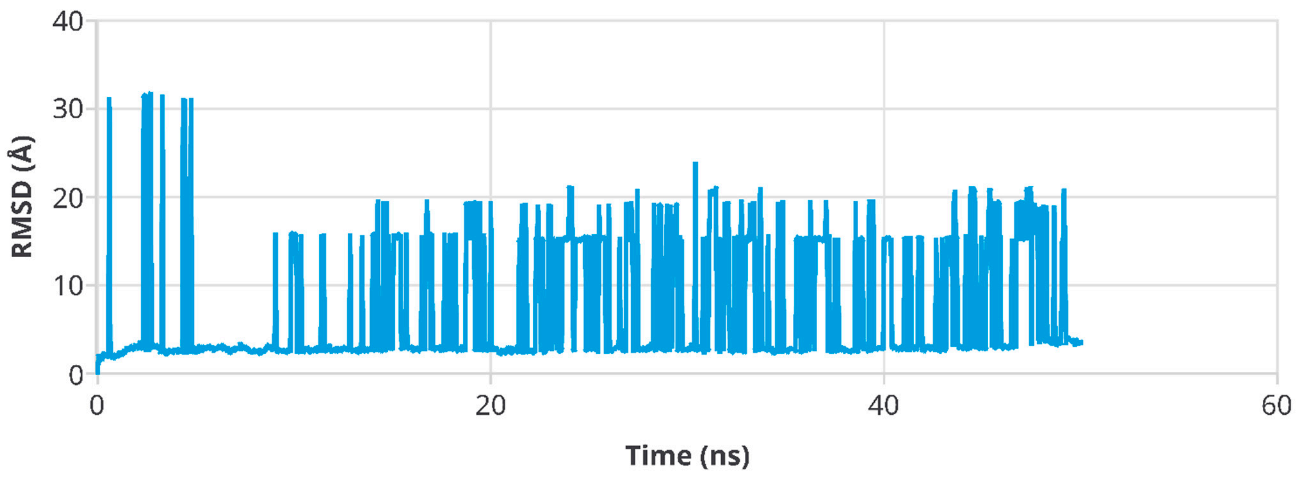

RMSF

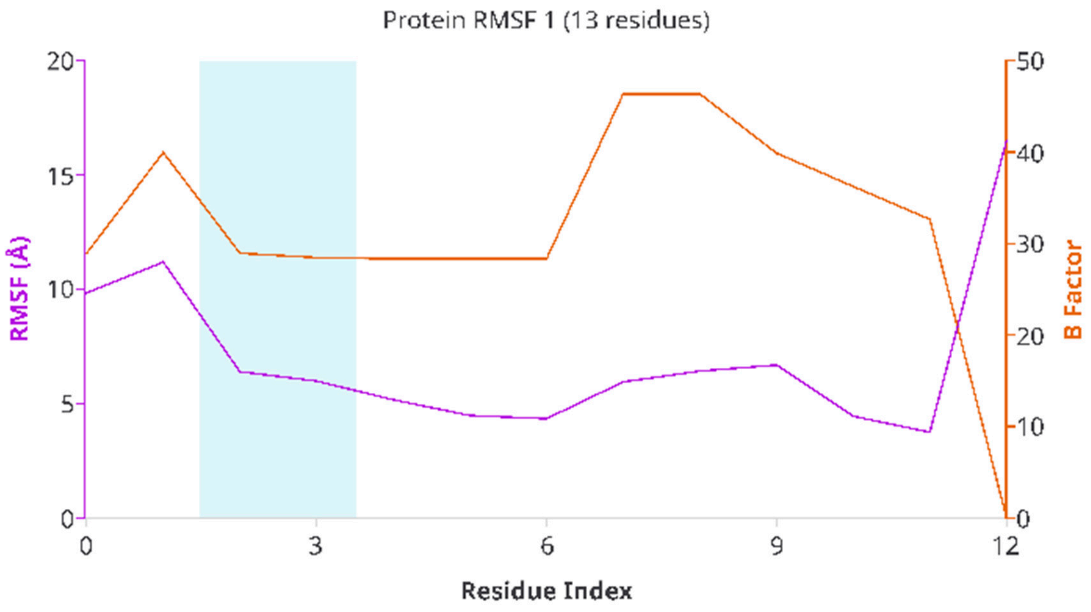

Radius of Gyration

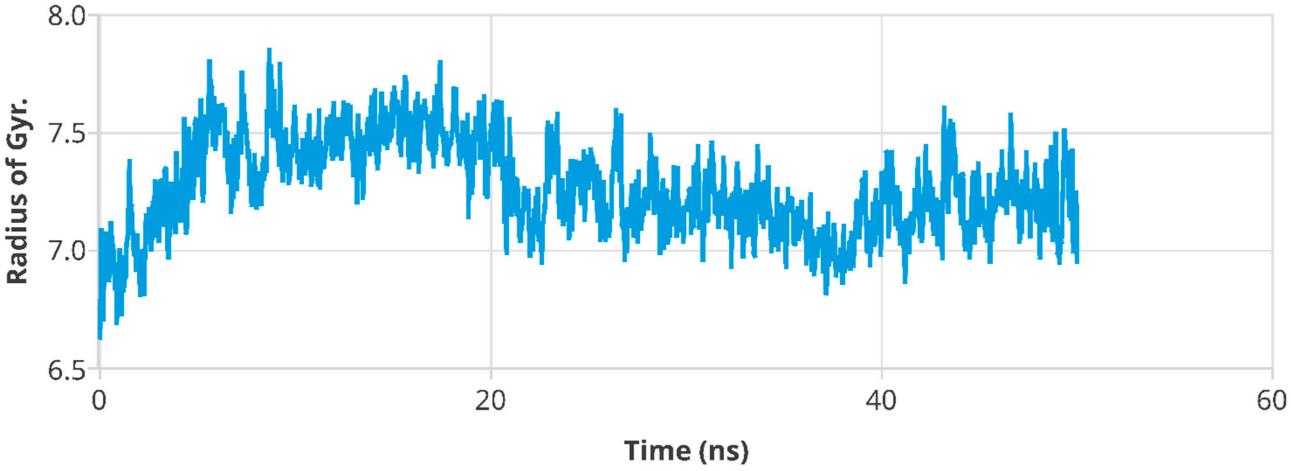

Molecular Surface Area

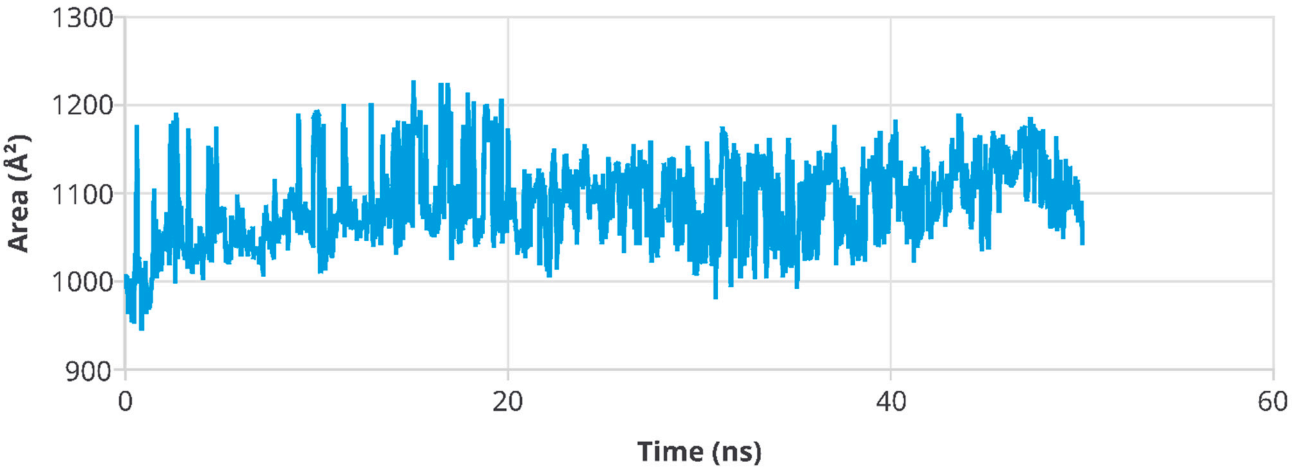

Polar Surface Area

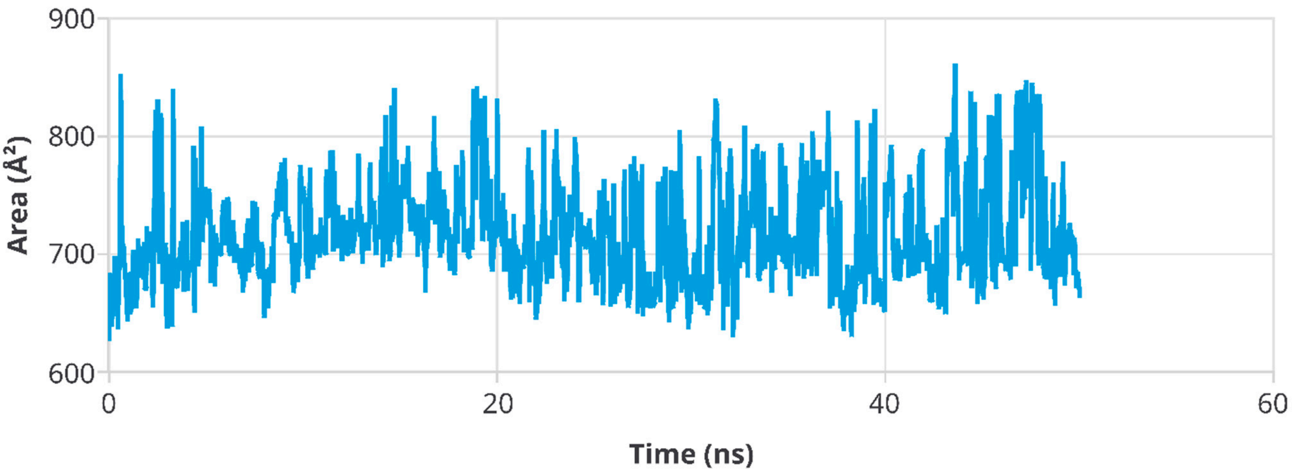

Radial Distribution Function (RDF)

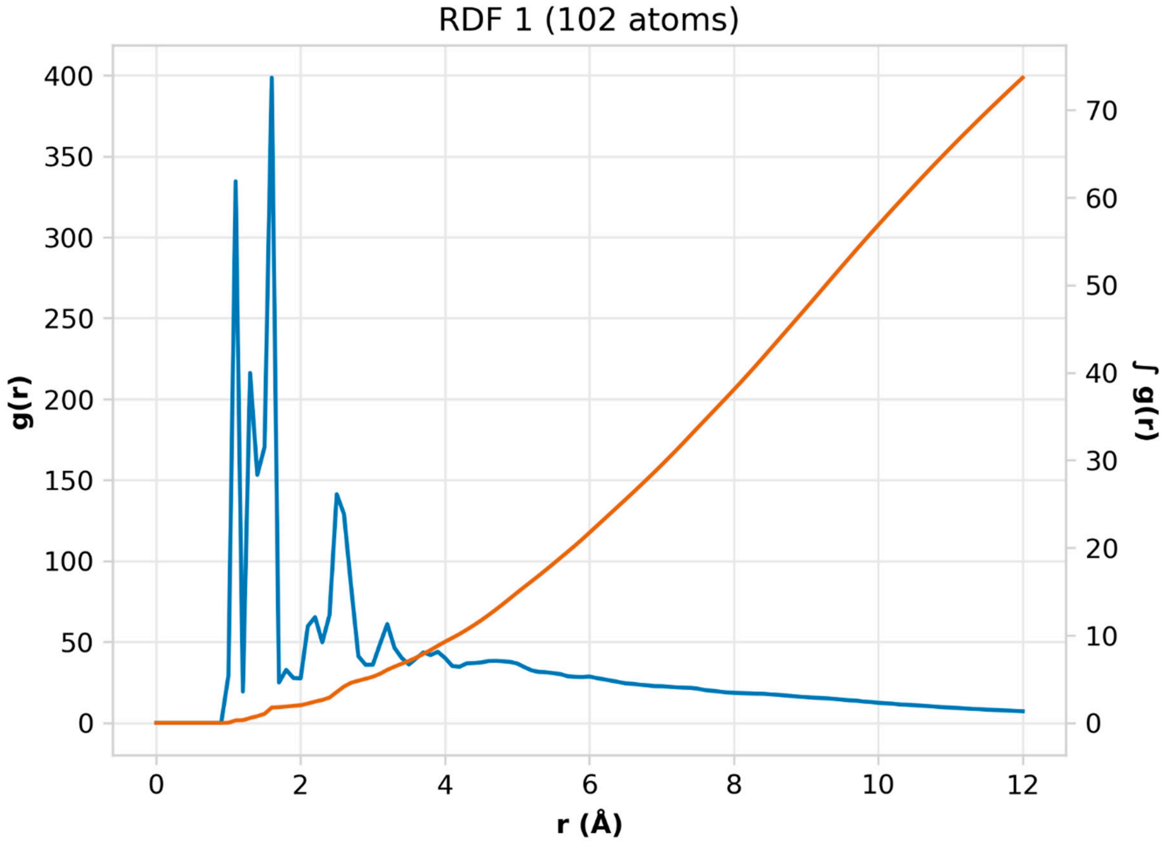

Solvent accessible surface area

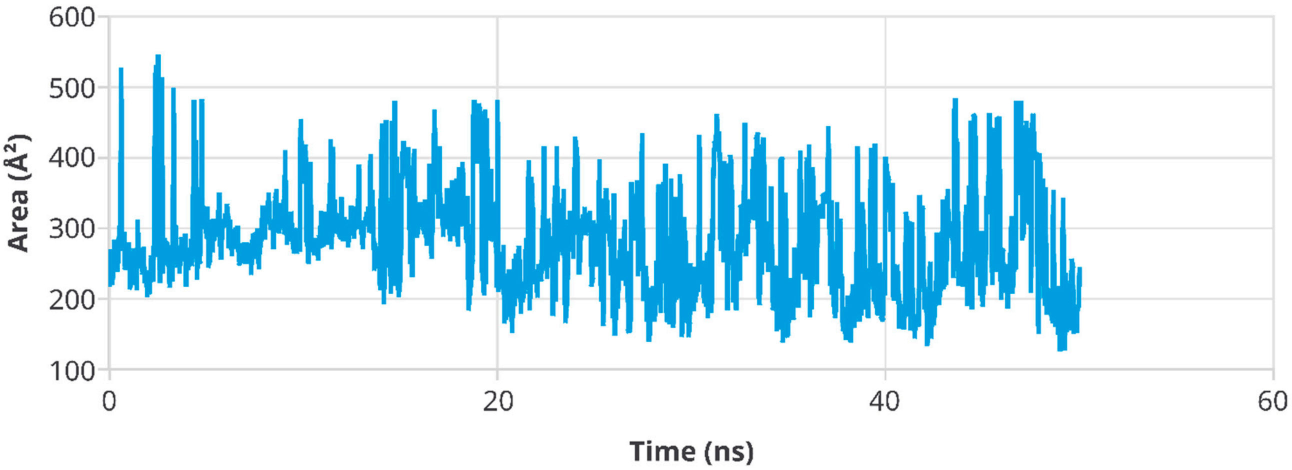

Supplement: Supplementary file 1 [file viruses-17-00951-s001.zip › viruses-3704435-supplementary.pdf]
